# Symmetry and fluctuation of cell movements in neural crest-derived facial mesenchyme

**DOI:** 10.1242/dev.193755

**Published:** 2021-05-07

**Authors:** Adrian Danescu, Elisabeth G. Rens, Jaspreet Rehki, Johnathan Woo, Takashi Akazawa, Katherine Fu, Leah Edelstein-Keshet, Joy M. Richman

**Affiliations:** 1 Life Sciences Institute, 2350 Health Sciences Mall, University of British Columbia, Vancouver, V6T 1Z3, Canada; 2 Department of Mathematics, University of British Columbia, 1986 Mathematics Road, Vancouver, V6T 1Z2, Canada

**Keywords:** Craniofacial, Avian embryo, Frontonasal, Fluctuating asymmetry, Mathematical analysis, Live cell imaging, Organ culture, Actomyosin

## Abstract

In the face, symmetry is established when bilateral streams of neural crest cells leave the neural tube at the same time, follow identical migration routes and then give rise to the facial prominences. However, developmental instability exists, particularly surrounding the steps of lip fusion. The causes of instability are unknown but inability to cope with developmental fluctuations are a likely cause of congenital malformations, such as non-syndromic orofacial clefts. Here, we tracked cell movements over time in the frontonasal mass, which forms the facial midline and participates in lip fusion, using live-cell imaging of chick embryos. Our mathematical examination of cell velocity vectors uncovered temporal fluctuations in several parameters, including order/disorder, symmetry/asymmetry and divergence/convergence. We found that treatment with a Rho GTPase inhibitor completely disrupted the temporal fluctuations in all measures and blocked morphogenesis. Thus, we discovered that genetic control of symmetry extends to mesenchymal cell movements and that these movements are of the type that could be perturbed in asymmetrical malformations, such as non-syndromic cleft lip.

This article has an associated ‘The people behind the papers’ interview.

## Abstract (precis)

**Highlighted Article:** Live imaging of the chick embryo face followed by mathematical analysis of mesenchymal cell tracks captures novel fluctuations between states of order/disorder as well as symmetry/asymmetry, revealing developmental instabilities that are part of normal morphogenesis.

## INTRODUCTION

Facial morphogenesis requires successful passage through multiple delicate steps that must be perfectly orchestrated to give rise to the bilaterally symmetrical adult form. There is a tolerance for a certain amount of variation or developmental instability in the early stages of embryogenesis so that in the majority of individuals, developmental abnormalities do not occur. However, more significant disturbances will lead to congenital malformations, such as cleft lip with or without cleft palate, the most common craniofacial abnormality in humans (Beames and Lipinski, 2020). The typical orofacial cleft is an isolated or non-syndromic condition known as non-syndromic cleft lip with or without cleft palate (NSCLP) (Beaty et al., 2016). The etiology of NSCLP is multifactorial, involving additive effects of multiple gene variants and interplay with environmental factors. The evidence from large studies on facial clefting shows that gene variants contribute to only 30% of the risk of NSCLP, the rest being the result of environmental stresses (Ludwig et al., 2017). The key role of environment is further highlighted by the disconcordance for cleft lip in 50-60% of monozygotic twin pairs (Grosen et al., 2011; Leslie et al., 2017).

The theory of developmental instability is that it is a continuum and is inherited to some degree (Van Dongen, 2007). The main way to measure developmental instability is via right-left symmetry comparisons (Ekrami et al., 2020, 2018). The greater the fluctuation or randomness of the asymmetry (no right or left predominance), the greater the developmental instability. These fluctuations in symmetry are different to the asymmetry caused by a specific insult, such as a unilateral injury or infection. In NSCLP, the prediction is that relatives of individuals with clefts should have more fluctuating asymmetry compared with control populations. This hypothesis has been tested by several groups. Surface scans of adult faces showed significant asymmetries in specific parts of the face in unaffected relatives compared with controls (Roosenboom et al., 2017; Weinberg et al., 2009; Zhang et al., 2018). There is also increased variation in skeletal morphology (Ceuninck and Starbuck, 2019) that correlated with increased risk of facial clefting (Richmond et al., 2018). These data reinforce the hypothesis that developmental instability exists in the embryo and that sometimes buffering is inadequate and a cleft will result (Thornhill and Møller, 1997). In this study, we sought to understand the sources of developmental instability in the upper face at the cellular level.

The origins of the face begin in the cranial neural crest cells that begin to migrate out from the neural plate at 3 weeks gestation (O'Rahilly and Müller, 2007) and move into the ventral side of the embryo where the face will form. The right and left streams of neural crest cells migrate and are patterned independently of each other. Although some preprogramming is present in premigratory neural crest cells, as shown by quail-chicken chimeras, most of the Hox-negative facial neural crest is interchangeable (Couly et al., 2002; Noden, 1978). Hox-negative cranial neural crest cells are the key repository of patterning information for the jaws (Couly et al., 1993; Noden, 1978; Schneider, 2018; Schneider and Helms, 2003). Hence, a decrease in survival or proliferation of neural crest cells will lead to a bilateral or unilateral reduction in the size of the jaws as observed in Treacher Collins syndrome, hemifacial macrosomia or after exposure to ethanol (Vega-Lopez et al., 2018). Thus, one reason for developmental instability is the variation in neural crest cell migration and proliferation on the right versus the left side of the face.

Once the neural crest-derived mesenchyme reaches the presumptive face, frontonasal, maxillary, lateral nasal and mandibular buds begin to grow out to surround the oral cavity. In order to make the upper lip, three of the facial prominences need to fuse together: the frontonasal mass (equivalent to mammalian medial nasal prominences), and maxillary and lateral nasal prominences. A series of developmental events need to occur: contact between the facial prominences, adhesion of epithelial surfaces, breakthrough of the mesenchyme to form a bridge; and proliferation of the mesenchyme to fill out residual grooves (Abramyan and Richman, 2015). Disruption at any stage will cause unilateral cleft lip (NSCL), the most common type of non-syndromic orofacial cleft (Gundlach and Maus, 2006; Leslie and Marazita, 2013). Developmental instability in the embryonic face at the cellular level may allow environmental factors to alter development on one side of the face, leading to a spectrum of facial abnormalities, from mild asymmetry to cleft lip with or without cleft palate (Richmond et al., 2018). Indeed, precise comparisons of lip fusion in several mouse strains (normal and cleft lip liable) found that the timing of when fusion was initiated was more variable in the cleft lip-liable strains (Wang et al., 1995). There is also asymmetry of the jaw skeleton, derived from cranial neural crest cells in the normal human fetus (Katsube et al., 2019).

Studies in mouse models have clearly shown that even when the genetics is precisely controlled, there is incomplete penetrance of cleft lip. In the majority of craniofacial studies, unilateral cleft lip is induced but only in a subset of embryos (Everson et al., 2017; Ferretti et al., 2011; Jin et al., 2012; Juriloff and Harris, 2008; Losa et al., 2018; Parsons et al., 2008; Song et al., 2009). All of these studies were performed using static observations at discrete time points (usually 12 h apart); therefore, the dynamic transition between stages was not observed. Closely timed observations need to be made as well as measurements on both sides of the face (Wang et al., 1995) as there may be an increase in fluctuating asymmetry at an early stage prior to lip fusion.

The hypothesis we explore here is that fluctuating asymmetry occurs at the cellular level and particularly in the mesenchyme. There is evidence from several types of static studies that a degree of cell movement is present in facial mesenchyme. Focal labeling studies carried out with ^3^H-thymidine (Patterson et al., 1984), dye spots (Lee et al., 2004; McGonnell et al., 1998), transfection with GFP plasmids (Geetha-Loganathan et al., 2014) or genetically labeled mouse embryos (Kaucka et al., 2016; Tao et al., 2019) report gradual spreading of the label. However, in these studies, cell spread is due to a mix of proliferation, cell migration and displacement. To date, only one live-imaging experiment has been conducted on the face in a mouse model (Tao et al., 2019) but the future lip was not examined.

In the present study, we used a chicken embryo model in which live imaging has been carried out in several different contexts (although not the face) (Bénazéraf et al., 2017; Gros et al., 2010; Kulesa et al., 2010; McColl et al., 2018; Voiculescu et al., 2007). Here, we imaged the frontonasal mass in organ culture because this prominence contained the facial midline and is the region affected in NSCLP. Using high-resolution live imaging, we captured the movements of mesenchymal cells across the entire prominence. We used unbiased mathematical approaches and detected fluctuations in cell behaviors over time. We also demonstrate that disruption of the actomyosin network causes a loss of temporal fluctuations, causing a loss of symmetry and uncoordinated cell movements. We suggest that a certain level of cell movement is normal but that there are times when these movements change. It is at these points of change when the face may be susceptible to an environmental insult.

## RESULTS

### Convergence-extension is a part of normal frontonasal morphogenesis

**Fig. 1.**
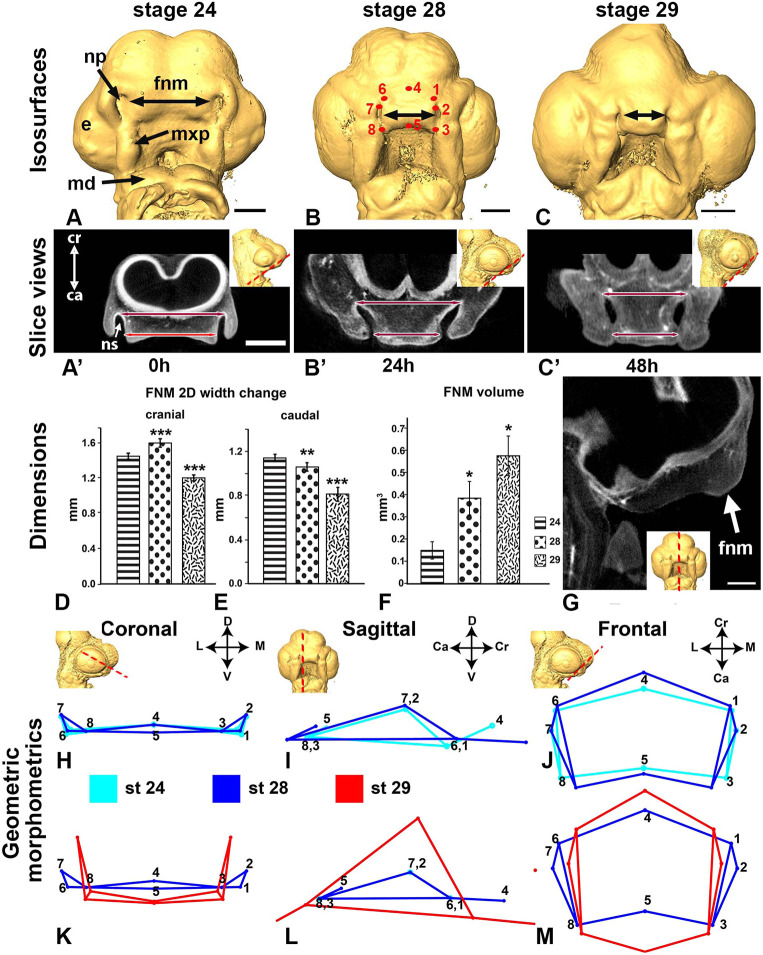
***In vivo* morphogenesis of the chicken embryo face.** Analysis using 2D and 3D morphometrics pinpoints the stages when major narrowing and outgrowth of upper beak takes place. (A-C) Between stage 24 and 29 the nasal slits are gradually relocated more medially in the frontonasal mass. (A′-C′) Slice views through the frontonasal mass (as shown by the dashed lines in insets) show maximum and minimum distance between the nasal slits (double-headed arrows). (D,E) Absolute distance significantly decreases with increasing stage. (F) Volumetric increases in the frontonasal mass. (G) Stage 28 embryo showing increased outgrowth in the dorsoventral axis. Dashed line in inset shows midsagittal plane of the slice view. (H-M) Wireframes showing mean displacement of eight homologous 3D landmarks for three stages of development (see B for landmark positions). (H-J) Stage 24-28 differences were mainly observed in the craniocaudal and mediolateral axes. (K-M) The displacement of landmarks was significant in all three axes. Scale bars: 500 µm (in A′ for A-C′);. 250 µm (G). Ca, caudal; Cr, cranial; D, dorsal; e, eye; fnm, frontonasal mass; L, lateral; M, medial; md, mandibular prominence; mxp, maxillary prominence; np, nasal pit; ns, nasal slit; V, ventral. *P<0.05, **P<0.01, ***P<0.001 (one-way ANOVA with Tukey's post-hoc test, *n*=8 for each stage in D-F,H-M).

We used the chicken embryo for our studies because the neural crest cell contributions to the face have been determined and because the midface appears to be more similar to that of human (Diewert and Lozanoff, 1993) than the mouse, which has a deep midline furrow (Hu and Marcucio, 2009b). We began by characterizing the key shape changes during frontonasal mass morphogenesis for live-imaging experiments. Scans of whole chicken heads covering stages 24-29 (Hamburger and Hamilton, 1951), equivalent to human morphogenesis at 6-7 weeks gestation (Richman and Vora, 2017), were reconstructed (Fig. 1A-C′). The linear 3D and 2D measurements of the frontonasal mass showed that there were complex changes in the distance between the nasal slits depending on embryonic stage and position (Fig. 1A′-C′,D,E; Fig. S1). The head increased in width by 38% whereas the absolute distance between the nasal slits decreased significantly (Fig. S1). The dorso-ventral thickness of the frontonasal mass mesenchyme also increased (Fig. 1G) as did the volume (3D segmentations; Fig. 1F; Fig. S2A). During the same period, proliferation is known to increase, particularly near the tip of the beak as a prelude to defining upper beak morphology (Wu et al., 2006).

To identify more specifically where shape changes were occurring, we carried out landmark-based 3D geometric morphometrics (Klingenberg, 2011) on segmented frontonasal masses (landmarks shown in Fig. 1B and Fig. S2A). Principal component analysis showed a separation of the shape by stage (Procrustes ANOVA, *P*<0.0001; Fig. S2B). Most of the variation (81.5%) was present in component 1 (Fig. S2C). Between stages 24 and 28, there was medial displacement of the lateral landmarks and extension of the cranial-caudal landmarks with minimal extension in the dorsoventral axis (Fig. 1H-J). By stage 29, there was significant extension in both the dorsoventral (Fig. 1K,L) and craniocaudal (Fig. 1M) axes with convergence in the medial axis (Fig. 1M). We also compared average centroid size and found that there were significant shape changes only between stage 29 and stage 24 (Fig. S2D).

**Fig. 2.**
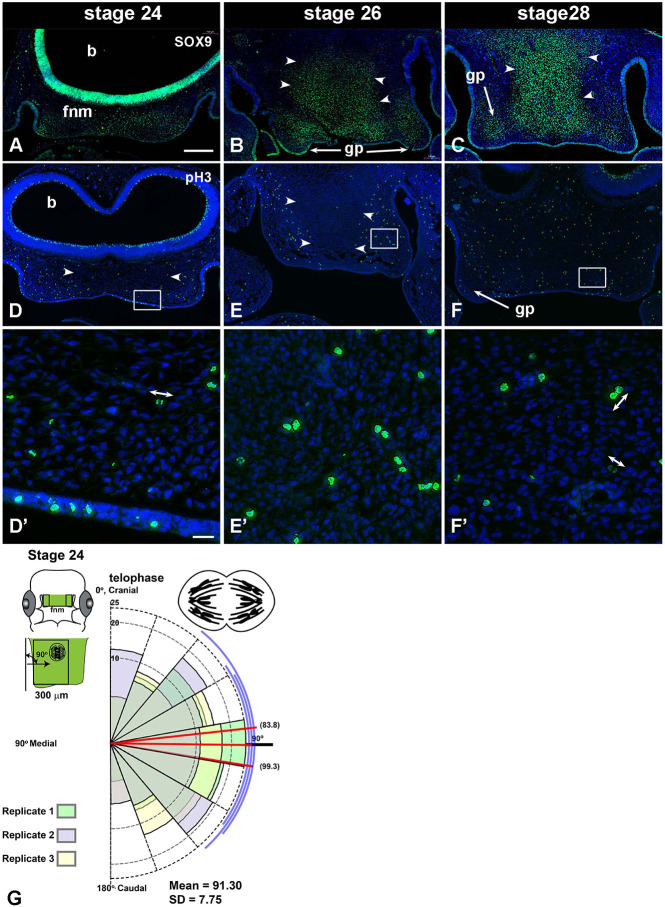
**Mapping chondrogenesis and proliferation in frontonasal mass mesenchyme.** (A-C) SOX9 staining marks cells that are fated to become chondrocytes. Staining is increased in non-proliferating regions in the center of the frontonasal mass at stages 26 and 28 (white arrowheads). (D-F′) There are fewer pH3-stained cells in the center (white arrowheads in D,E). (D′,F′). In higher magnification images of the boxed areas in D-F, cells in mitosis or just after cytokinesis are visible (double-headed arrows). (G) The angle of cell division was measured by comparing the axis of cell division to the long axis of the nasal slit (green in schematic). The polar plot shows the angular data from three biological replicates (see coloured fills for replicates 1,2,3). The number of observations are plotted in bins of 20° between 0 and 180°. The means for each biological replicate are shown by red lines. The mean angle of cytokinesis is 91.3° to the cranio-caudal axis. A total of 185 cells were measured across three replicates (approximately 60 nuclei per replicate). Scale bars: 200 µm (A-F); 20 µm (D′-F′). b, brain; fnm, frontonasal mass; gp, globular process.

The shape changes, particularly at stage 29, could have been driven by the differentiation of the prenasal cartilage, a derivative of the frontonasal mass (Richman and Tickle, 1989). We mapped SOX9 expression, one of the earliest markers of chondroprogenitor cells (Celá et al., 2016) as well as COL2A1, the main matrix component of cartilage. There was diffuse expression of SOX9 at stage 24 (Fig. 2A) but by stage 26 there was a central expression domain overlapping the future region of the prenasal cartilage condensation (Fig. 2B), which became more defined at stage 28 (Fig. 2C). The SOX9 expression suggested early commitment of midline cells to a chondrogenic fate. However, there was a delay before COL2A1 was expressed (Fig. S3A,B). No signal was detected at stage 29; therefore, secretion of matrix was not a main driver of shape changes.

### Orientation of cell division does not explain extension of the frontonasal mass

One of the mechanisms of morphogenesis is oriented cell division, whereby progeny are preferentially produced in one axis. We predicted that there would be many cells that would divide in the cranio-caudal plane, where the most extension was observed (Fig. 1J). However, unexpectedly, mesenchymal cells were observed dividing in the perpendicular, mediolateral plane (Fig. 2D′-G). There did not seem to be a right or left preference for cells dividing at specific angles (Fig. S4). Not all cells could be scored because some were in prophase or perhaps were dividing in the perpendicular plane. We therefore sectioned other embryos in the coronal plane and found that there were significantly fewer nuclei with visible division planes compared with the frontal sections [frontal, *n*=6, 18.4±3.0% mitotic cells in MATC (metaphase, anaphase, telophase and cytokinesis); coronal *n*=6, 7.3%±1.84 mitotic cells in MATC; two sample equal variance, two-tailed *t*-test, *P*<0.0001]. This decrease in scoreable cells in the coronal plane may mean that we missed cells that were dividing in the craniocaudal axis. New cells are added preferentially to the lateral edges of the frontonasal mass, likely under the stimulation of signals from the nasal slit (Szabo-Rogers et al., 2008). As oriented cell division does not contribute to midfacial extension, we investigated whether growth of the surrounding structures, such as the eyes and brain, indirectly caused the frontonasal mass to narrow and extend.

### *In vitro* cultures of the face demonstrate that the frontonasal mass undergoes morphogenesis independently of the eyes and brain

**Fig. 3.**
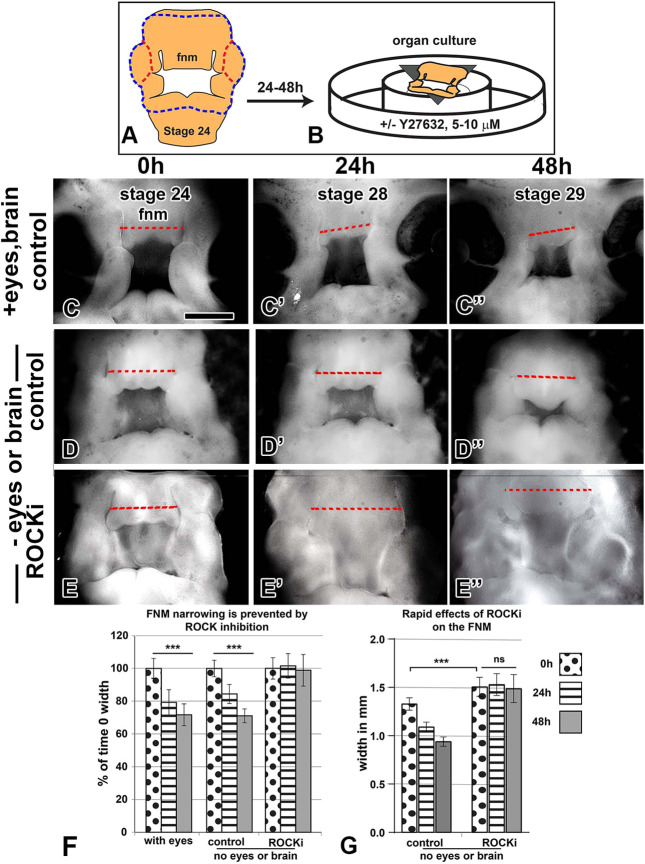
***In vitro* organ culture of the facial prominences supports morphogenesis.** (A,B) Schematic of the organ culture. (A) The blue line outlines the full face plus eyes. The red outline shows the cuts used to remove the eyes. (B) The epithelium is uppermost at the air-liquid interface. (C-E″) Serial images of organ cultures. Facial prominences reached approximately stage 29 after 48 h of culture. (C-D″) Narrowing at the midpoint of the nasal slits (dashed line) occurs with or without the eyes and brain. (E-E″) Cultures treated with either 5 or 10 μM Y27632, have a wider frontonasal mass than controls. (F) Measurements taken across the frontonasal mass (red dashed lines) show that normal cultures decreased in width whereas ROCKi-treated cultures did not. Graph shows width relative to that at time 0 for each condition. (G) Absolute measurements of images collected about 2-4 h after adding ROCKi (time 0 data) show that there is a rapid increase in width of the frontonasal mass. The size of the frontonasal mass does not change between time 0 and 48 h of culture once the ROCKi has been added. ***P<0.001 determined with repeated measures ANOVA and Tukey's post-hoc test (*n*=12). ns. not significant. Fnm, frontonasal mass. Scale bar: 1 mm.

How much of frontonasal mass morphogenesis is intrinsically regulated? In the chicken embryo, there are well-documented signals that come from the brain to set up the face at early stages. *SHH* is expressed in the floorplate of the diencephalon and this signal is required to induce the frontonasal mass epithelial growth zone that controls morphogenesis (Hu and Marcucio, 2009a; Young et al., 2010). The epithelial growth zone itself is capable of inducing a branched upper beak (Hu et al., 2003). Initially, we dissected the frontonasal mass with the brain and eyes, always including the frontonasal epithelial zone, and placed it into culture (Fig. 3A,B). The readout for morphogenesis was the decrease in internasal distance. As seen *in vivo*, the frontonasal mass narrowed *in vitro* (*n*=12; a decrease of 30% by 48 h compared with 0 h; Fig. 3C-C″,F). Surprisingly, the narrowing rate was not changed significantly when the eyes and brain were removed (*n*=12; Fig. 3D-D″,F). We also removed the maxillary and mandibular prominences, leaving just the nasal slits, frontonasal mass and lateral nasal prominences. Again, narrowing still took place (Fig. S5A-H). Thus, the frontonasal mass grown with the epithelium was able to undergo morphogenesis independently of signals from the brain or the eyes. The width of the frontonasal mass in the organ cultures tracked very closely to the *in vivo* dimensions of stage 24, 28 and 29 embryos (Fig. 3G; Fig. S1).

We next investigated whether the cells themselves were providing shape information. We treated cultures with Y27632, which interferes with Rho and Rac small GTPases (Wei et al., 2001). The Rho family small GTPases, such as Rho, Rac and Cdc42, regulate actomyosin remodeling (Denk-Lobnig and Martin, 2019). We found that the ROCK inhibitor (ROCKi) significantly widened the frontonasal mass by the time the first images were taken (approximately 1 h after inhibitor addition). Then, as the experiment progressed, there was no significant change in width (*n*=12; Fig. 3E-G). These data suggest that ROCK regulation of the cytoskeleton is essential for normal frontonasal morphogenesis.

**Fig. 4.**
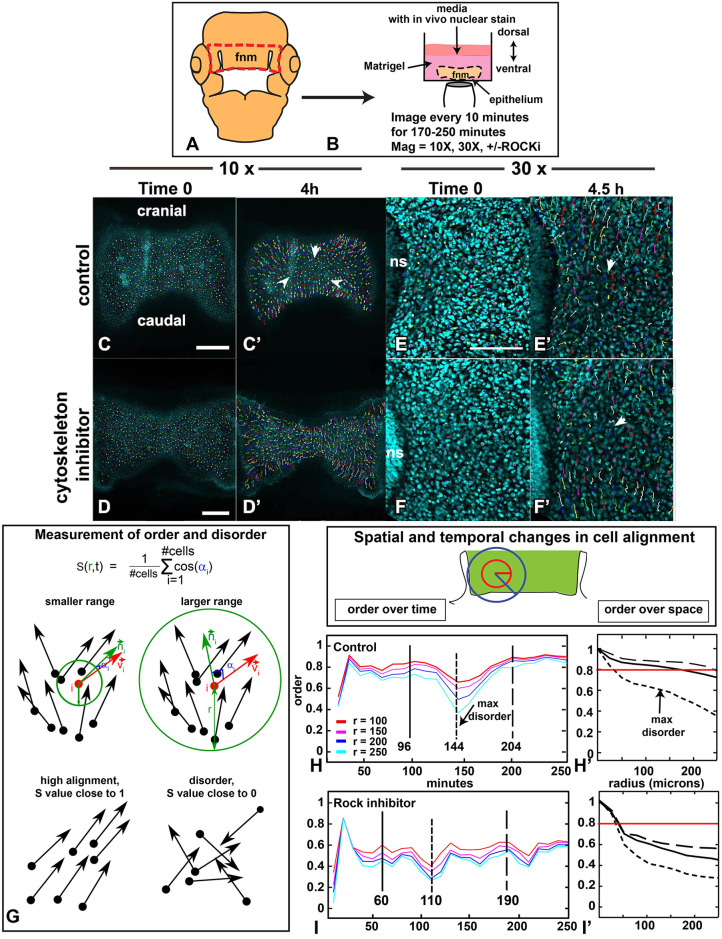
**Manual tracking of cells in the frontonasal mass and calculation of directional order.** (A,B) Schematic showing the set-up for live imaging. The red line outlines the area dissected for imaging. The epithelium side is facing down, covered with Matrigel and then media layered on top. (C,C′) The dot is the end of the track and the tail shows all movement of that cell. The tracks in the center of the frontonasal mass appear shorter (white arrowheads, C′). (D,D′) ROCKi was added and the culture spread out relative to controls. After 4 h, the ROCKi culture had not changed in width (D′). (E,E′) The nasal slit is on the left of the images. Tracks appear to be polarized with cranial and caudal tracks moving away from each other. Tracks in the center appear shorter (white arrowhead, E′). (F,F′) The ROCKi culture (10 µM) has shorter tracks (white arrowhead). (G) Directional order, *S*(*r,t*), calculated over radius *r*. Green circles, circular neighborhoods; red arrows, cell velocity (*v_i_*); green arrows, mean direction within neighborhood of radius *r* (*n_i_*); blue, angle between vectors (α*_i_*). *S*(*r,t*)**=**average of cos(α*_i_*) over the tissue at time *t*. *S*(*r,t*) is expected to decrease with increased *r*. Cells moving in the same direction will have *S*∼1. Random movements, *S*∼0. (H-I′) Vector direction at fixed times was used to determine *S* as a function of time *t*. In H′,I′, the solid line is the first peak of order, the short dashed line is the point of maximum disorder, the longer dashed line is the second peak of order. Note that alignment drops for *r>*200 µm (H′), particularly in ROCKi treatment (I′). Red horizontal line indicates directional order of 0.8. Fnm, frontonasal mass; ns, nasal slit. Red circle is 100 µm in radius and blue circle is 200 µm radius. Scale bars: 250 µm (C-D′);100 µm (in E for E-F′).

We next customized a live-imaging system that would capture the motility of individual frontonasal mesenchymal cells at high resolution. Cultures were grown in Matrigel inside the imaging chamber of an inverted confocal microscope (Fig. 4A,B). Comparisons were made between control and ROCKi-treated cultures after 4.5 h of growth. There was no significant difference in proliferation (Figs S6A, S7A,B) or density (Figs S6B, S7A′,B′). There were also no qualitative differences in the shape of cells (Fig. S7C,D). The ROCKi prevented facial narrowing such that cultures were significantly wider than controls (Fig. S6C). The ROCKi caused no observable increases in apoptosis (Fig. S7E,F); therefore, overall health of the tissue was maintained.

### Dynamic cell movements occur in the frontonasal mass mesenchyme and these are regionally coordinated

Having optimized the conditions, a series of live-imaging experiments were carried out (Table 1). A nuclear stain was used to reduce interference from overlapping cell membranes (Fig. 4C-F′; Fig. S7C,D). We tracked hundreds of nuclei across the frontonasal mass at relatively low magnification, thus permitting us to compare cell movement on each side of the face over time (Table 1, Fig. 4C-D′; Movies 1,2). We also collected higher-magnification images next to the nasal slit, a signaling region in the frontonasal mass (Szabo-Rogers et al., 2008) (Fig. 4E-F′, Movies 3,4).

**Table 1.**
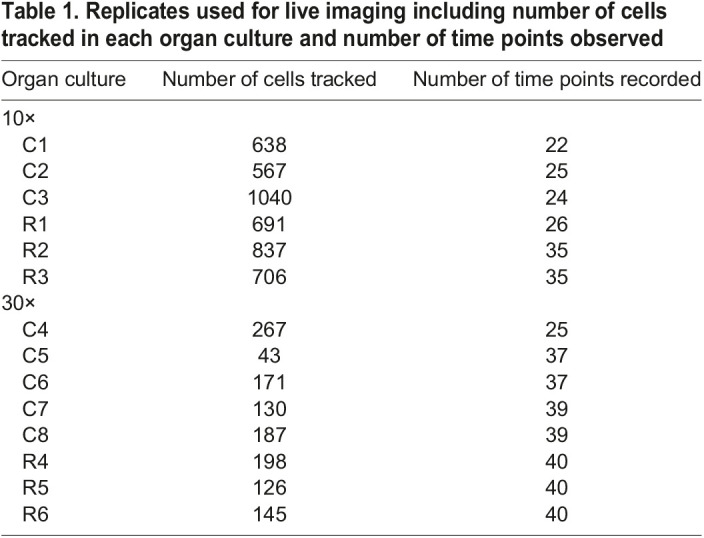
Replicates used for live imaging including number of cells tracked in each organ culture and number of time points observed

### Mathematical analysis of cell tracking data over time and space

To assess all the tracks throughout the experiments, we exported the *xy* positions at each timepoint from ImageJ and used these coordinates for mathematical analyses. First, we quantified the alignment of cell movement directions, *S* (*r,t*), across time (Petitjean et al., 2010; Slater et al., 2013). For each time point, *t*, and radius, *r*, we found the angle (α_i_) between each cell's velocity vector (***v_i_***) and the local mean velocity vector (*n**_i_***) (averaged over a neighborhood of radius *r*). The neighborhoods were repeated across the frontonasal mass, to cover all the tracks. We then averaged cosine (α_i_) over the tissue to obtain *S* (*r,t*), and repeated for each *r* and *t* (Fig. 4G; see supplementary Materials and Methods).

The degree of alignment of cell movement, *S* (‘order’ in Fig. 4H,I; Fig. S8), depends on the direction the cells take relative to their neighbors and the radii of the circular neighborhoods tested. Tissues in which cells move in a similar direction as their neighbors will have *S* values close to 1, whereas disorganized movement will have *S* values close to 0 (Fig. 4G). An *S*∼1 is expected for small radii, because cells that are close together have a greater probability of moving in similar directions (Fig. 4G). However, as cell-cell communication decreases over distance, *S*∼0 or disorder is expected for bigger radii (Fig. 4G).

We plotted the order *S* as a function of time. We found surprising oscillating periods of order and disorder in the control cultures (Fig. 4H-I′; Fig. S8B-D′). During the first 20 min, cultures achieved peak order at *S*=0.8-0.9 (Fig. 4H,I; Fig. S8B-D). A second peak of order occurred after the first 100 min, followed by a trough 40-50 min later and a recovery in the last hour of the culture period (Fig. 4H; Fig. S8B-D). Surprisingly, the value for *S* remained at 0.8 or higher for the two periods of order in the control cultures (Fig. 4H′; Fig. S8B′-D′). These fluctuations in order and disorder may be present during normal development of the frontonasal mass. The order (*S*) decreases as a function of *r* for three representative time points (Fig. 4H′,I′). In controls, alignment (‘ordered movement’) can be maintained up to *r* ∼ 150/250 µm. Note that a radius of 200 µm is approximately one-quarter of the frontonasal mass (Fig. 4H, blue line), indicating long-range spatial coordinated cell movement in the controls.

In contrast to the controls, ROCKi-treated samples lacked clear periods of order and disorder throughout the culture period (Fig. 4I; Fig. S8E-G′). In addition, there was a loss of order in radii larger than 50 μm (Fig. 4I′).

### K-means clustering analysis reveals regional coordination across the frontonasal mass

In order to visualize the regions of aligned cell movement across the frontonasal mass, we used unbiased k-means clustering. This statistical approach clusters data by similarity of a measurement, such as gene expression levels or, in this case, vector direction. We first clustered vectors based on cell position in the frontonasal mass and vector directions (Method 1, testing effects of changing the weight of position, *p*, or vector, *q*); see supplementary Materials and Methods). We selected three time points that represent the fluctuation in order and disorder customized to each culture – the first was a time when order was above 0.8, the second when disorder was seen (trough), and the third when order was again above 0.8 (red line in Fig. 4H',I').

**Fig. 5.**
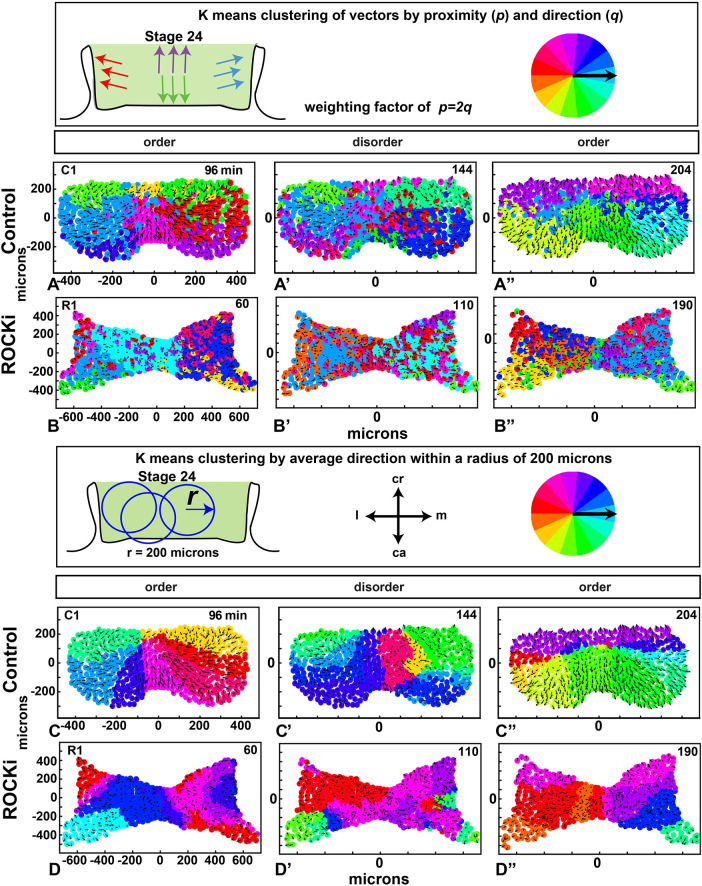
**K-means clustering of vectors.** (A-B″) K-means clustering by proximity and direction (Method 1) with relative weights *p*=2, *q*=1 at the three time points of high order, disorder and order (as in Fig. 4H,I) for one control (A-A″) and one ROCKi (B-B″) treated culture. (For all data, see Figs S8 and S9.) The black arrows are cell velocity vectors. Color wheel indicates the average direction of each cluster. (C-D″) K-means clustering by average direction within a radius of 200 µm (Method 2). See Movies 5-8. C1, control culture replicate 1; ca, caudal; cr, cranial; l, lateral; m, medial; R1, ROCKi-treated culture replicate 1.

We noted that the weight, *p* (position), *q* (vector angle), affects the clustering result. With *p=*0, *q*=1, clusters appeared spatially diffuse in controls and even more so after ROCKi treatment (Fig. S9A-B″). At the other extreme, *p*=1, *q*=0, the tissue was clustered into nine equally shaped rectangular blocks, each one with mixed cell directionality (Fig. S9C-D″). Eventually, we settled on *p*=2*q*, for the relative weights as this led to spatially connected clusters of aligned cells (Fig. 5A-A″; Movie 5). The clusters were less coherent in the ROCKi-treated group (Fig. 5B-B″; Movie 6). In the controls, by the end of the experiments, we observed more distinct clustering across the frontonasal mass (Fig. 5A-A″; Fig. S10), suggesting stabilization of coordinated movement. Cell cluster assignment changed over time, as cell directions relative to their neighbors changed over the developmental period (Movie 5). The edges of the clusters of cell vectors (areas where the average direction of the cells differs from neighboring cells) may not form as consistently in the presence of environmental stresses. For example, any stress that affects the cytoskeleton could prevent regional clustering, as we observed with the ROCKi group (Fig. 5B-B″; Movie 6; S10D-F″).

Next, we used local mean directions (***n_i_***) over a 200-µm radius neighborhood, which tends to smooth the data and reduce noise from local fluctuations (Method 2, see supplementary Materials and Methods). We chose a neighborhood radius *r*=200 µm because previous bead implant experiments showed that growth factors or small molecules affect gene expression and proliferation within a range of roughly 200-300 µm away from the bead (Higashihori et al., 2010; Szabo-Rogers et al., 2008), suggesting that cells within *r*=200 µm could be acting coherently based on the spatial range of tissue signals. Although this motivated our choice of *r*, lower values of *r* (e.g. 50, 100 µm) produced similar results using Method 2 (Fig. S11A-D″).

We found that clusters are spatially connected using Method 2 (despite the fact that spatial position is not considered in the clustering) (Fig. 5C-C″; Fig. S12A-C″; Movie 7). There are fewer, more well-defined clustered regions compared with Method (1) weighted on either position or vector direction (roughly six compared with nine). Clustered regions fluctuated more near the beginning and ended with a more stable pattern of clusters towards the end of the cultured time period. Clustering in the disordered state (Fig. 5,C′) is less meaningful, because, based on previous results, we know that cell alignment drops at a radius scale of 50 µm (Fig. 4H′,I′, dashed curve; Fig. S11A-A″). Interestingly, in the controls, cells from the cranial and caudal edges of the frontonasal mass moved in opposite directions (Fig. 5C-C″; Fig. S12C-C″; Movies 5 and 7), correlating with extension in this axis seen *in vivo* (Fig. 1J,M).

ROCKi-treated cultures had more diffuse, disorganized clusters at all time points (Fig. 5D-D″; Fig. S12D-F″; Movie 8). This lack of cell coordination could have been caused by an inability of mesenchymal cells to respond properly to local signaling (including moving or reorienting themselves towards or away from a signal). In addition, the ROCKi treatment disrupted individual cell behaviors.

Our clustering results, especially those for which a radius of 200 μm was imposed, quantify coherent directions of motion in the frontonasal mass.

### Right-left symmetry in cell movement is present in the frontonasal mass

**Fig. 6.**
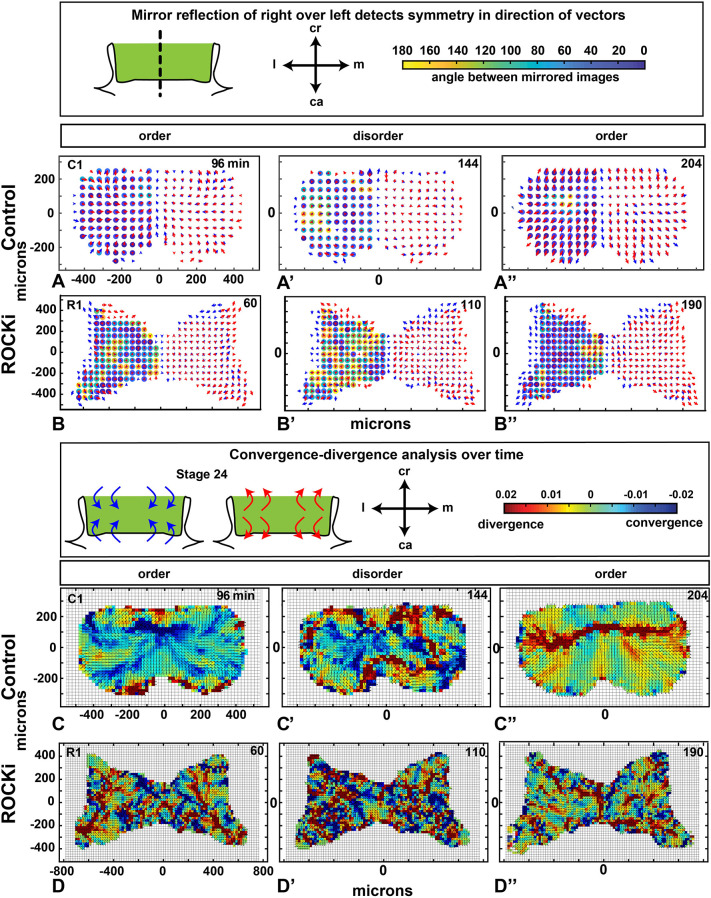
**Right-left symmetry and convergence/divergence are disrupted by ROCKi.** Interpolated data (neighborhood averaging of 50 µm) at the three representative times (Fig. 4H,I). (A-B″). (A-A″) High left-right symmetry in ordered states for the control (blue). (B-B″) Clear disruption of symmetry is caused by ROCKi (more yellow). (C-D″) Divergence and convergence (see color scale at the top) across the frontonasal mass. (C-C″) In controls, the first time point had regions of convergence (C). In the disordered state, there were areas of convergence spread across the frontonasal mass (C′). At the end of the cultures, a major band of divergence crossing the frontonasal mass mediolaterally was observed (C″). (D-D″) ROCKi treatment completely disrupted the patterns of convergences and divergence. See Movies 9 and 10. C1, control replicate 1; ca, caudal; cr, cranial; l, lateral; m, medial; R1, ROCKi replicate 1.

The degree of asymmetry is thought to be a readout of developmental instability. For this reason, we measured fluctuating asymmetry over time of individual cell vectors in our samples. The left-side data also serves as a technical replicate of the right side. We first averaged vectors in a 50-µm radius, allowing us to compare the differences at the equivalent grid location (Fig. S13A-D″). There was a high degree of symmetry in the control cultures, particularly in the periods of high order (Fig. 6A,A″). Cells in the left-right portions of the mass appeared to be intermittently coordinating their behavior to maintain symmetry. In states of disorder, there were more asymmetric movements observed (Fig. 6A′,B′; Fig. S14A-C″). This pattern was consistently disrupted in the ROCKi-treated samples in which asymmetry predominated (Fig. 6B-B″; Fig. S14D-F″). Coordination over the extent of this range of 400-500 µm (radii of 200-250 µm) suggests a combination of long-range signaling, cell preprogramming and/or genetic control.

### Mapping divergence and convergence within the frontonasal mass

**Fig. 8.**
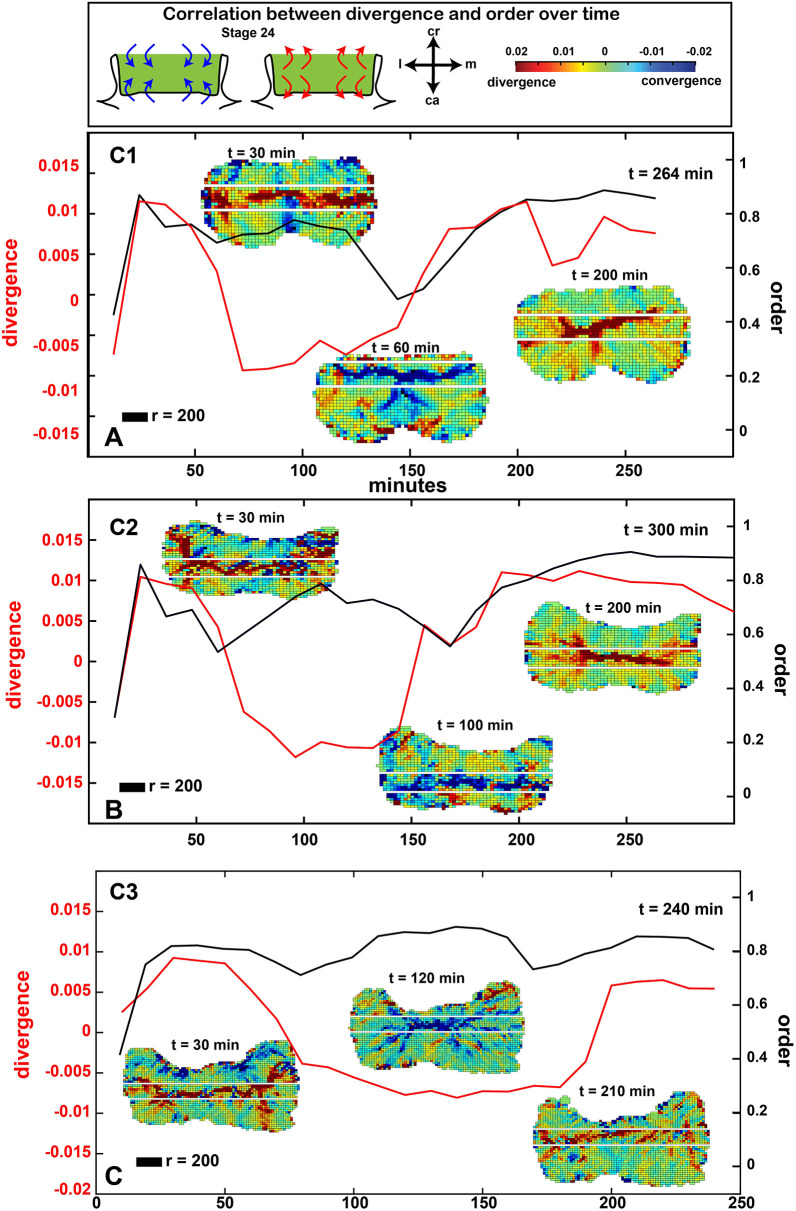
**Oscillations of divergence and order are correlated over time.** (A-C) Plots of the fluctuations in divergence and convergence over time in control cultures. The black line reproduces changes in order and disorder (for *r*=200 µm). The red line quantifies the average divergence/convergence computed in the mediolateral band (area between the white lines) covering the grid areas with greatest number of pixels in either red or blue (see supplementary Materials and Methods). All cultures start out with divergence for the first 60-80 min. Then a period of convergence lasts about 100 min before returning to divergence for the end of the culture. The switches between patterns of convergence and divergence are rapid (10-20 min each). See Movie 9. C1-3, control replicates 1-3; ca, caudal; cr, cranial; l, lateral; m, medial.

We hypothesized that in order for the frontonasal mass to narrow, there needs to be mediolateral convergence of the cell tracks. In addition, the divergence of cell tracks would be present cranially and caudally based on the geometric morphometrics. We quantified areas of divergence or convergence where vectors were averaged over a 50 μm radius. Grid points were placed every 20 μm in order to make the patterns clearer but averaging was still done at 50 μm. We used a common descriptor of vector-field properties, divergence [div(***v***)]. This scalar quantity measures the local tendency of a vector field to point towards or away from each point in space. Given the refined interpolated set of cell velocity vectors (normalized to be unit vectors), we compute the divergence at every point in our grid, and visualize the magnitude as a spatially distributed color field (Fig. 6C-D″; Fig. S15; see supplementary Materials and Methods). Indeed, during high order, vectors generally converged towards the center (Fig. 6C; Fig. S15A-C). During disorder, there were no clear patterns visible (Fig. 6C′; Fig. S15A′-C′), but at the end of the culture, tracks diverged towards the cranial caudal axis (Fig. 6C″; Fig. S15A″-C″; Movie 9). The culture initially exhibited a mediolateral band of divergence between 10-40 min before switching to convergence and then back to divergence at the end. All the control cultures displayed the same pattern (Fig. 8). As predicted from the clustering and symmetry data, ROCKi completely abolished the bands of convergence and divergence (Fig. 6D-D″; Fig. S15D-F″; Movie 10). There were also unpredicted results from the analysis. Based on the tendency for the frontonasal mass to narrow, we expected that cells would converge towards a single vertical line in the cranio-caudal axis. Instead, the cells were gathered towards the center of the frontonasal mass from all corners of the prominence.

**Fig. 7.**
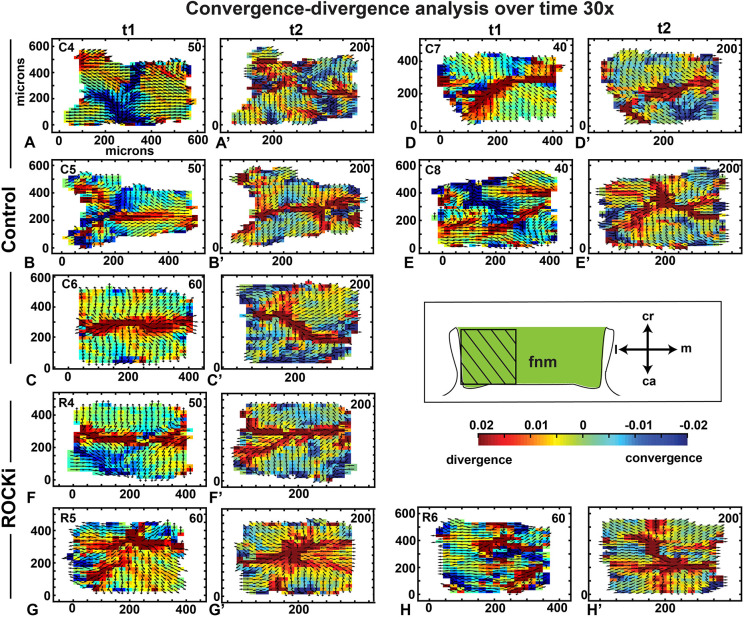
**Convergence-divergence analysis over time at 30× magnification** (A-H′) A smaller area of the lateral frontonasal mass was imaged (boxed area in schematic). Nasal slit is to the left. Data were interpolated over a grid of 50 µm. (A-E′) At the first time point, there were cultures with vertical bands of convergence (A,B,E) whereas the other two cultures primarily had divergence patterns (C,D). Bands of divergence were seen in all five control cultures at the later time point (A-E′). (F-H′) The ROCKi cultures showed lines of divergence but with less clear organization. See Movies 11 and 12. C4-8, control replicates 4-8; ca, caudal; cr, cranial; fnm, frontonasal mass; l, lateral; m, medial; R4-6, ROCKi replicates 4-6.

To examine local cell behavior further, we carried out analysis on a region close to the nasal slit, a primary source of FGFs at higher magnification. First, we checked whether there were periods of order and disorder; however, there were no clear patterns (Fig. S16). Early in the cultures, areas of convergence were sometimes seen close to the nasal slit (Fig. 7A,B,E). The remaining cultures were diverging (Movie 11) between 20 and 50 min (Fig. 7C,D). After 1 h, cultures had mainly a mediolateral band of divergence with several branches similar to those observed at the edges of the 10× data (compare Fig. 6C-C″ imaged at 10× to Fig. 7A′-E′ imaged at 30× magnification). The ROCKi-treated cultures imaged at 30× displayed divergent vectors for the entire culture period with extensive branching (Fig. 7F-H′; Movie 12).

We then used the 30× magnification data to test whether directed cell migration was taking place in the frontonasal mass. The higher resolution imaging was needed to test this hypothesis. The ROCKi caused a decrease in the overall instantaneous speed (Fig. S17A, *P*=0.009), which may reduce directed cell migration. Indeed, we discovered regional differences in cell behavior, somewhat analogous to the unbiased mathematical clustering (Fig. 5; Figs S9-S12). First, there was relatively less displacement in the center than at the cranial and caudal edges of the frontonasal mass (Fig. S17B; compare regions 4 and 6 with region 5 as shown in the schematic in Fig. S17D). We measured straightness of the cell tracks using the autocorrelation function. Here, cells were more highly directional in the cranial and caudal regions (regions 4 and 6 in the schematic in Fig. S17D) compared with the center (Fig. S17C). The ROCKi decreased displacement (Fig. S17B) but did not appear to interfere with directionality of the cells (Fig. S17C). In point of origin plots, we confirmed that the majority of cranial and caudal cells were diverging from the center (Fig. S17D). There was slightly less polarity to the data from ROCKi-treated cells (Fig. S17E). Thus, ROCKi had the effect of slowing cell movement, especially in the cranial and caudal regions.

### Oscillating patterns of convergence-divergence across time

We next wanted to know whether the changes in divergence and convergence patterns correlated with the switch between the order-disorder phases. To answer this, we let the computer algorithm define a band of maximum convergence-divergence and computed the average divergence within that band over time (Fig. 8A-C; see supplementary Materials and Methods). We observed that all control cultures started with a divergence band that lasted for around 60 min of culture, followed by another hour during which a convergent band was observed across the frontonasal mass, and, lastly, a culture period in which the divergent band returned (Fig. 8A-C). Interestingly, this dynamic switching occurred in a 10-20 min time interval, suggesting rapid oscillation between two opposing states. We also observed that dips in order correlate with changes from convergence to divergences (or vice versa). There was more order present when cultures were switching from one direction to the other, a trend seen in all three cultures in the middle of the culture period (Fig. 8A-C).

## DISCUSSION

**Fig. 9.**
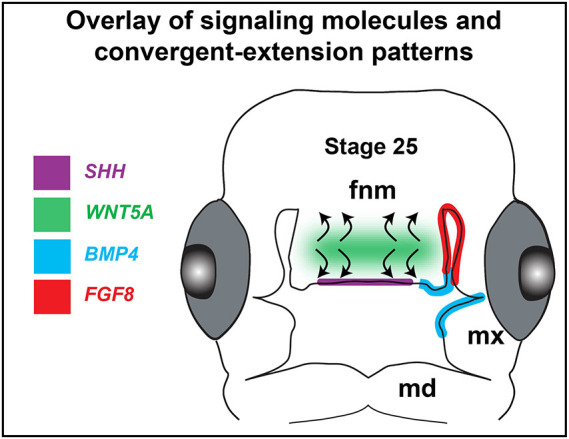
**Overlay of gene expression with patterns of divergence and convergence.** Overview of gene expression and proliferation in the frontonasal mass at stage 25. *BMP4*, *SHH* and *FGF8* are expressed in the epithelium whereas *WNT5A* is expressed in the mesenchyme in a medio-lateral band overlapping the region that oscillates between convergence and divergence patterns of cell movements (curved arrows). Fnm, frontonasal mass; md, mandibular prominence; mxp, maxillary prominence.

Although we have not yet tested specific cytokines or teratogens in this work, we have identified patterns of movements in cells that are likely to be affected by multiple signaling pathways. In mouse and chicken experiments, morphogenesis of the frontonasal mass or the midface is regulated by growth factors, such as WNT5A (Kaucka et al., 2017), FGF8 near the nasal slits (Szabo-Rogers et al., 2008), SHH (Marcucio et al., 2015) and BMPs (Ashique et al., 2002; Hu et al., 2015) (Fig. 9). It is possible that FGFs could promote cell movements near the nasal slits, although directional information may not be provided as shown in the limb bud (Gros et al., 2010). BMPs regulate proliferation, lip fusion and patterning as shown by Noggin bead implants (Ashique et al., 2002; Celá et al., 2016). SHH regulates outgrowth of the beak as shown by experiments blocking signaling (Hu et al., 2015). Of all of these networks, the prime candidate for cell movements observed here is the non-canonical, JNK-PCP WNT pathway. The JNK signal results in cytoskeletal changes that affect cell shape, orientation, polarity and directed cell migration (Butler and Wallingford, 2017). WNT5A protein induces JNK-PCP signaling activity as shown in facial and limb mesenchyme (Geetha-Loganathan et al., 2014; Gignac et al., 2019; Hosseini-Farahabadi et al., 2013, 2017). *WNT5A* is expressed in a horizontal band across the frontonasal mass at the stages we imaged here (Geetha-Loganathan et al., 2009) (Fig. 9). This band of expression lines up with the computed band of convergent-extension. More direct evidence is needed to determine whether the *WNT5A* signal waxes and wanes; however, we have learned from experiments with the ROCK antagonist that the downstream signaling pathways must be operational in order to see organized fluctuations in cell behavior in the frontonasal mass.

Mouse gene-targeting experiments also support a specific requirement for Rho GTPase signaling in morphogenesis of the midface. Three tissue-specific knockouts of Cdc42 using P0-cre (a neural crest cell cre driver) (Oshima-Nakayama et al., 2016) or Wnt1Cre (neural crest cells) (Fuchs et al., 2009; Liu et al., 2013) or Prrx-Cre (facial mesenchyme) (Aizawa et al., 2012) cause a wide midfacial cleft. *Rac1* was also knocked out conditionally with Wnt1-Cre (Thomas et al., 2010) and a transgene expressing a dominant-negative form of Rho kinase (Phillips et al., 2012) also gave rise to the same phenotype. Rho GTPase signaling is therefore required for midfacial morphogenesis as shown by mouse genetic studies and our chicken cultures treated with a ROCK antagonist.

### Oscillation between convergence/divergence, order/disorder and symmetry is evidence of fluctuating asymmetry

Our study yielded several novel results that were not anticipated. We found that the frontonasal mass reaches a stage at which development proceeds independently of signals from the brain and face. This coincides with the time when lip fusion begins. The positions of mesenchymal cells do change in a dynamic fashion across the whole organ and these movements appear to be coordinated over defined regions. In addition, we did not expect that within the imaging period we would encounter some longer cycles, such as the convergent-extension movements that lasted about an hour each (Fig. 8A-C). Underneath these convergent-extension movements there are smaller scale fluctuations as seen with order/disorder, K-means clustering and symmetry. All of these changes in cell behavior are suggestive of an intrinsic developmental instability that exists as part of normal frontonasal mass development. Morphogenesis continues despite the oscillating cell behaviors.

The cell movements that we observed here are on a much smaller scale (estimated to be 2.5 μm over 4 h) than the several millimeters of travel seen in the collective migration of lateral line epithelial cells in the zebrafish or in migrating neural crest cells (Schumacher, 2019). Even though the movements are less than the size of a cell, the mathematical analysis has revealed that they are reproducible across our replicates. Our symmetry analyses are sensitive enough to have ruled out uneven effects of Matrigel on the culture. We have also shown that these cell movements are dependent on GTPase signaling and that blocking this pathway causes a flattening and widening of the culture.

We were surprised to see rapid switching of cell directions from divergence to convergence and back, taking place over a time interval of 10-20 min. These switches in direction were correlated with transitions from order to disorder. In addition, symmetry became weaker during the period of disorder. The biochemical mechanisms are still unclear, but a signal such as Rho GTPase fits the remit. It is well-known that Rho GTPases are prime regulators of the cytoskeleton, forming central hubs in many of the intracellular signaling cascades. Those hubs receive feedback from many sources, whether local (e.g. cell-cell and cell-extracellular matrix adhesion), long-range (chemical signaling via WNT, FGF, etc., or mechanical tension from neighbors or from distant cells pulling on the extracellular matrix). In recent years, a combination of experimental observations and mathematical modeling (Kholodenko, 2006; Park et al., 2017) has demonstrated that the interplay of feedback loops in such signaling networks can give rise to rich sets of dynamics, including cycles of activity, and even spatiotemporal patterns. Disruptions of nodes in such pathways (e.g. the ROCKi inhibition of Rho) can then abrogate or change the wild-type pattern, as we have seen here. In short, the reversal of directions of cells and the break in left-right tissue symmetry is undoubtedly not merely genetically encoded, but also directly dictated by intracellular signaling, shaped by cell-cell interactions in the tissue. A rapid signal such as GEP/GDP is one possible moderator that could be easily susceptible to external stresses leading to such congenital anomalies as cleft lip.

### Direction of movement is correlated with anatomical location in the frontonasal mass

We applied a K-means clustering algorithm to identify regions where cells move in the same direction. The maintenance of clear boundaries between neighboring cell populations is required for proper frontonasal development, based on the appearance of control cultures. It does appear that there is a type of coherent repositioning of mesenchymal cells in the face that can be predicted by anatomical location. Certainly, the cranial-caudal divergence from the center was a trend repeated in all the control cultures at all magnifications. However, in ROCKi-treated samples, the clusters of cells were poorly delimited. In treated cultures, there was no evidence of a midline separating the right and left migration patterns. Mapping of clusters of cells will allow us to decipher regional cell responses – genetic and epigenetic – and then ultimately reshaping of the organ during normal and abnormal development.

With these imaging advances, we are seeing the face in a new light, in parallel with other innovations used to see developing embryos in real time (Wallingford, 2019). We first report that cell movements are necessary for frontonasal morphogenesis. Second, an unbiased mathematical clustering of the changes in cell position found surprisingly coordinated and periodic movements that were present across an entire organ. Third, the positional changes were largely symmetrical, thus providing the earliest mechanistic understanding of how facial symmetry is established.

Our results represent an overview of the collective cell motility that await the identification of mechanistic causes. Other factors, such as cell elongation, cell-cell or passive movement due to mechanical pulling by other parts of the tissue, may contribute to our findings. In the future, it will be fascinating to see how cell repositioning is affected in the presence of low levels of teratogens. This low-dose experiment will test the extent of buffering capacity in the tissue. If buffering is in place, then coordination and symmetry would be restored when those agents are removed. Finally, we expect that similar types of fluctuating cell behaviors are likely to be present in the other facial prominences and other organs. Our individual cell movement analysis may demonstrate that small fluctuations in cell position are in fact a widespread phenomenon.

## MATERIALS AND METHODS

### Animals and morphometric analysis of the face

Fertilized white leghorn chicken eggs (*Gallus gallus*) were purchased from the University of Alberta, Edmonton. Eggs were incubated for 3-5 days covering stages 20, 24, 28 and 29 (Hamburger and Hamilton, 1951). Embryos were fixed in 10% formaldehyde and processed into clearing solution for fluorescence-based, optical projection tomography (OPT) as published (Abramyan et al., 2015). Embryos were scanned on a wavelength that excites autofluorescence in formalin-fixed tissues. A total of eight embryos per stage (*n*=24) were scanned with OPT and reconstructed using NRECON software. Image stacks were visualized using Amira software (FEI v2019.2, Thermo Fisher). Linear measurements were made using the 3D measurement tool, which places marks directly on scanned surfaces. Each frontonasal mass was segmented manually so that volume could be calculated and so that landmarks could be applied on the ventral surfaces facing the brain. Segmented objects were exported as ply files to Landmark and five landmarks were applied as shown in Fig. 1. The landmarks were then exported in NTSYS format to MorphoJ and Procrustes superimposition was carried out. Wireframes were created and discriminant function analysis between two representative stages was carried out in order to visualize the major shape changes in 3D.

### Immunofluorescence and TUNEL staining

Paraffin sections were stained with a pH3 antibody (rabbit polyclonal, Cell Signaling Technology, 9701, 1:400), anti SOX9 (Sigma-Aldrich, HPA001758, 1:200), anti Col2A1 (Developmental Studies Hybridoma Bank, CIIC1-b, 1:250) and anti-Myosin II (Developmental Studies Hybridoma Bank, CM1123, 5 µg/ml). Standard antigen retrieval with DIVA Decloaker (BioCare Medical) was carried out. Species-specific secondary antibodies tagged with Alexa Fluor 488 or Cy5 were used (goat anti-mouse 488, A11029; goat anti-rabbit 488, A11034; goat anti-mouse Cy5, A10524, Life Technologies; all at 1:250).

Apoptosis was assessed by TUNEL analysis using the ApopTag Apoptosis Kit (Millipore, S7111) and was detected using fluorescein-tagged anti-digoxygenin antibody as described by Hosseini-Farahabadi et al. (2013). A qualitative assessment of presence of TUNEL-positive cells was carried out.

Nuclei were counterstained in Hoechst 33258 (Sigma-Aldrich, 10 µg/ml in PBS) for 30 min. Slides were mounted with Prolong Gold without DAPI (Invitrogen, P36934). Fluorescence imaging was conducted using a 20× objective on a Panoramic MIDI II slide scanner with 488, Cy5 and DAPI filter sets (3D Histech). High-magnification images were captured with CaseViewer software v.2.4.0.119028 (3D Histech).

### Oriented cell division, cell proliferation and cell density analysis

The proportion of cells in metaphase, anaphase, telophase or cytokinesis out of the total number of mitotic figures was determined in frontal and coronal sections. Three biological replicates were counted at stage 24, 26 and 28 in each plane of section. To gather enough cells, the values across four or five sections per animal were summed.

We fixed cultures at 4.5 h after incubation in the microscope chamber and used sections for cell density and proliferation analysis. The total number of pH3^+^ cells and total number of cells in the frontonasal mass were calculated for one section from each of eight specimens (biological replicates). To estimate total cell number, we used the cell density measurements and calculated the number of cells in the total area of the frontonasal mass. For density, we counted numbers of cells in seven 100 μm^2^ areas within each biological replicate. The mean value was calculated for each culture and then this number was used to calculate the average cell density in control and treated cultures. Unpaired, two-tailed, equal variance *t*-tests were used to compare means in the proliferation and cell density analyses.

### Facial organ cultures for static imaging

Chicken eggs were incubated until they reached stage 24-25. In full-face organ cultures, the frontonasal masses including/excluding the eyes and the brain were dissected in 1× Hanks's Balanced Salt Solution with 10% fetal calf serum on ice. The epithelium was left intact for all experiments. Embryonic faces were placed on top of Nucleopore membrane, epithelium side up and the membrane was supported by a wire mesh. Dissections either included all the facial prominences (Fig. 3A, dashed line) or just the frontonasal mass and nasal slits (Fig. 4A, dashed line). Cultures were grown at the air-liquid interface as described (Hu and Helms, 2001). Media was added to each well [900 µl; Dulbecco's Modified Eagle Medium (DMEM)/Ham's F-12, 1:1, with 15 mM HEPES buffer, L-glutamine, 10% fetal bovine serum, 1:100 penicillin/streptomycin/amphotericin B].

We performed a dose-response experiment using 2, 5, 10 or 100 µM ROCK inhibitor (Y27632, Calbiochem, 688000), which is a cell-permeable, highly potent and selective inhibitor of Rho-associated, coiled-coil containing protein kinase (ROCK). We tested a variety of doses as published (Wei et al., 2001) and determined that 10 µM dissolved in DMSO led to detectable changes while not inducing cell death. Lower concentrations had mild effects on cell speed but were too small in magnitude to measure differences in our initial studies. Control cultures were treated with 1 µl of DMSO to 250 µl of media, the same volume used to deliver the ROCKi.

### Live imaging of organ cultures

For live imaging, frontonasal mass and lateral nasal prominence explants at stage 25 were transferred to Ibidi microslides (80821, Ibidi) and flipped so that the epithelial surface was contacting the glass. Matrigel (100%, 80 µl, Thermo Fisher) was added on top of the cultures and forceps were used to keep the explant in contact with the dish while the gel set. Media was added to each well (150 µl DMEM:F12 buffered with fresh 20 µM HEPES to equilibrate with room air). This method prevented drift while still allowing 3D morphogenesis to take place. To visualize the nuclei in live cultures, 1 μM Hoechst 33258 was added to the culture media 1 h prior to imaging.

Confocal time-lapse microscopy with 10× and 20× objectives (1.5 zoom on the 20× objective) was carried out (Fig. 4B) using a Leica SP5 inverted confocal microscope with an environmental chamber (37°C) and motorized stage. Image stacks were collected every 10 min for 4-6 h (Table 1). Images were captured at 1024×1024 or 2048×2048 resolution (for 10×), 400 Hz, UV 405 laser (11% power). We imaged ten independent cultures at 10× magnification and six of these were of high enough quality throughout the experiment to be used for manual tracking (Table 1). Another eight cultures were imaged at 30× (Table 1).

### Post-processing of images

LIF files produced from Leica software were imported into Fiji (Fiji is ImageJ with additional plugins) using the Bioformats plug-in. Individual organ cultures were separated into stacks and saved as TIF files. Images were edited by using Maximum *z* Projection on four or five slices of 1 μm thickness in the stack to produce clear images. Images were drift corrected using the Linear Stack Alignment with SIFT plug-in (https://imagej.net/Linear_Stack_Alignment_with_SIFT). The plugin selects multiple points and follows them through the image to correct for drift due to extrinsic forces.

### Cell tracking

Two observers, blinded to the experiments, tracked the nuclei. The manual tracking plug-in on Fiji was used to follow nuclei. Dots were placed over the center of the nucleus for each time point. Any nuclei that could not be followed for the entire 24 frames (240 min) were omitted. The entire frontonasal mass was blanketed with tracks at 10×. The tracked images were exported with the options ‘Dots & Lines’ and ‘Overlay Dots’ into TIF files. The raw *xy* positional data were used for mathematical clustering and symmetry analysis.

### Mathematical analysis of cell tracking data using MatLab

See supplementary Materials and Methods for a detailed description of the mathematical analysis.

## Supplementary Material

Supplementary information

Reviewer comments
