# Supplementary material for: Symmetry and fluctuation of cell movements in neural crest-derived facial mesenchyme
Source: Development. 2021 May 7;148(9):dev193755. doi: 10.1242/dev.193755 (PMC8126411; doi:10.1242/dev.193755)
Supplement: Supplementary information [file develop-148-193755-s1.pdf]

Supplementary Figures

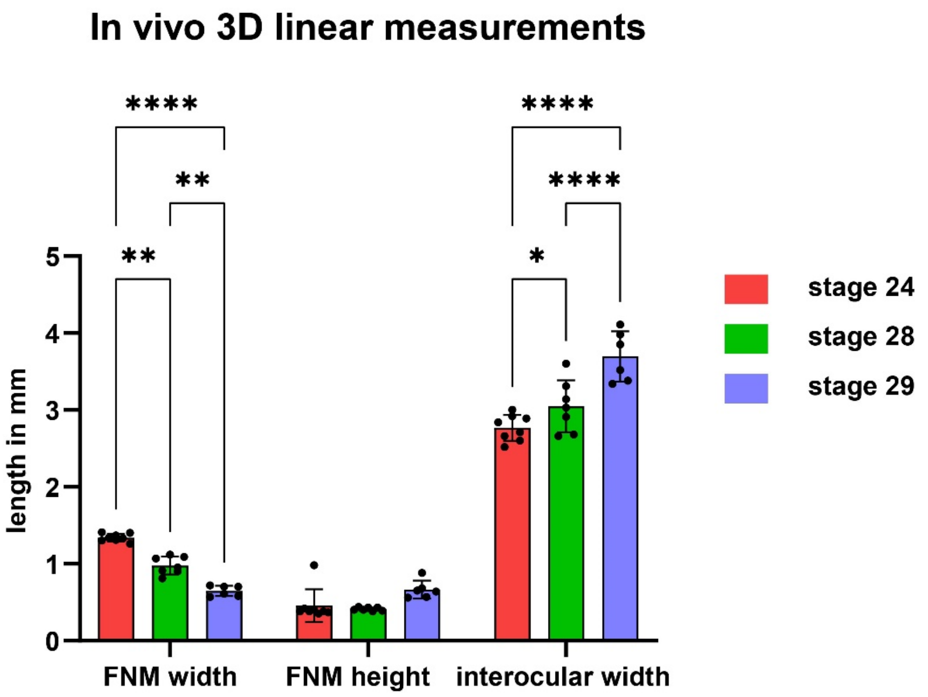

Figure S1. 3D linear measurements from reconstructed OPT scans of chicken embryo heads. Scans were imported into Amira software for visualization and measurements. Frontonasal mass width was measured at the midpoint between the cranial and caudal edges of the nasal slit. Frontonasal mass height was measured in the midline from a line connecting the top of the nasal slits to the caudal tip of the frontonsal mass. Interocular width was measured through the head between the two eyes. Significant decreases in absolute frontonasal mass width occurred between all stages. No significant increase in frontonasal mass height was detected however interocular with increased by approximately 38% when comparing stage 24 and 29. Thus the overall head size increases while the midface narrows. The frontonasal mass width values correspond very closely to the organ cultures started at stage 24 and grown for 48h (Fig. 3G). Key: FNM – frontonasal mass

Development • Supplementary information

Geometric morphometrics analysis of the frontonasal mass

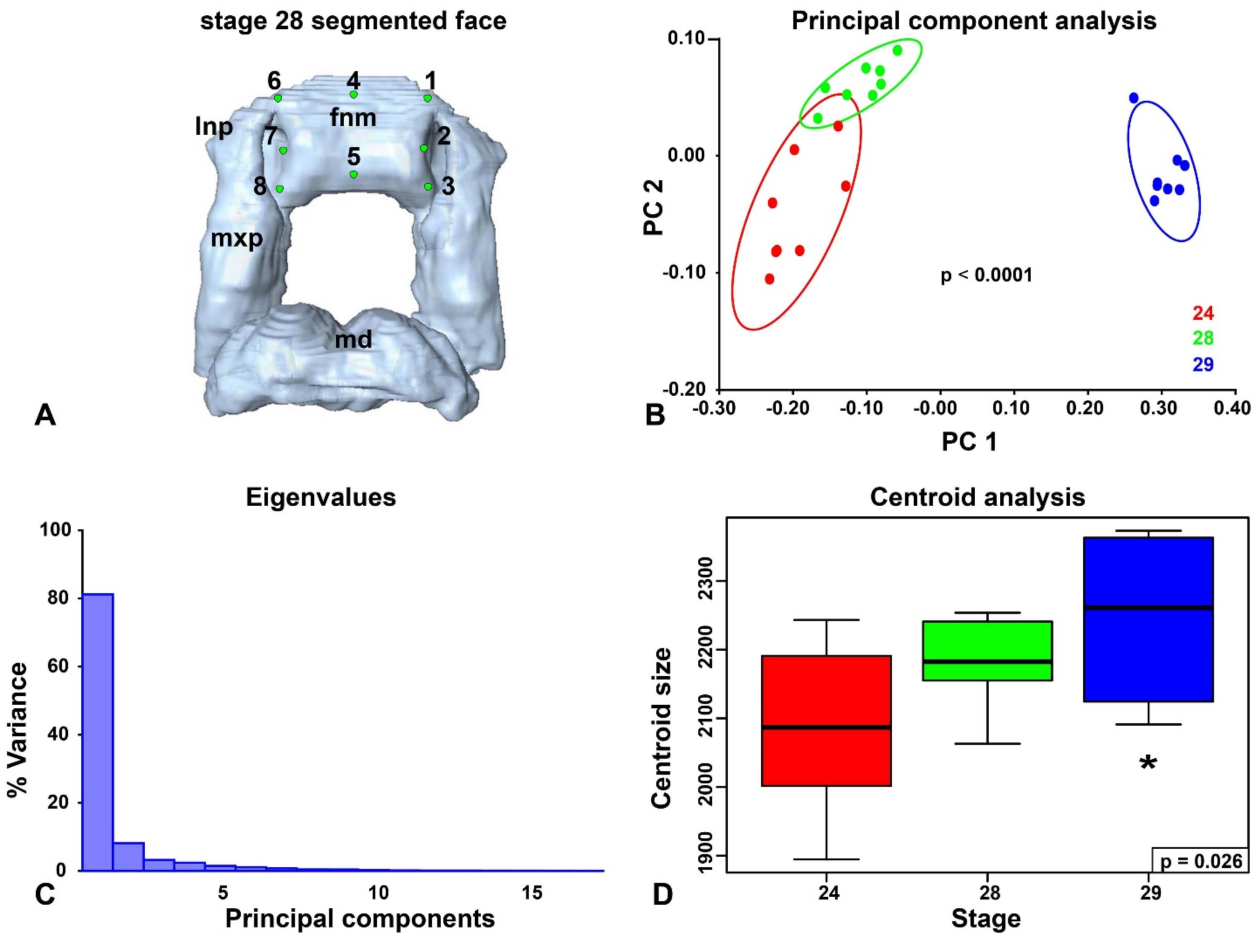

Figure S2. 3D Geometric Morphometrics analysis of the frontonasal mass at 3 stages of development. **A)** Placement of landmarks on a segmented face. By isolated the face from the head we were able to capture the thickness of the frontonasal mass with landmarks 1,6,7 and 8. B) The PC analysis revealed significant separation based on shape between stage 24 ,28 and 29 frontonasal mass. The low P value is due to the stage 29 shape being so different than the younger stages. C) The majority of the variance (more than 80%) is captured in PC1 which probably captures the extension of the cranial-caudal axis. D) The centroid sizes are similar at stage 24 and 28 but significantly larger in stage 29 embryos. Landmarks are more spread out at stage 29 which relates to increased growth. P value determined with Tukey's post hoc test for multiple comparisons.

Development • Supplementary information

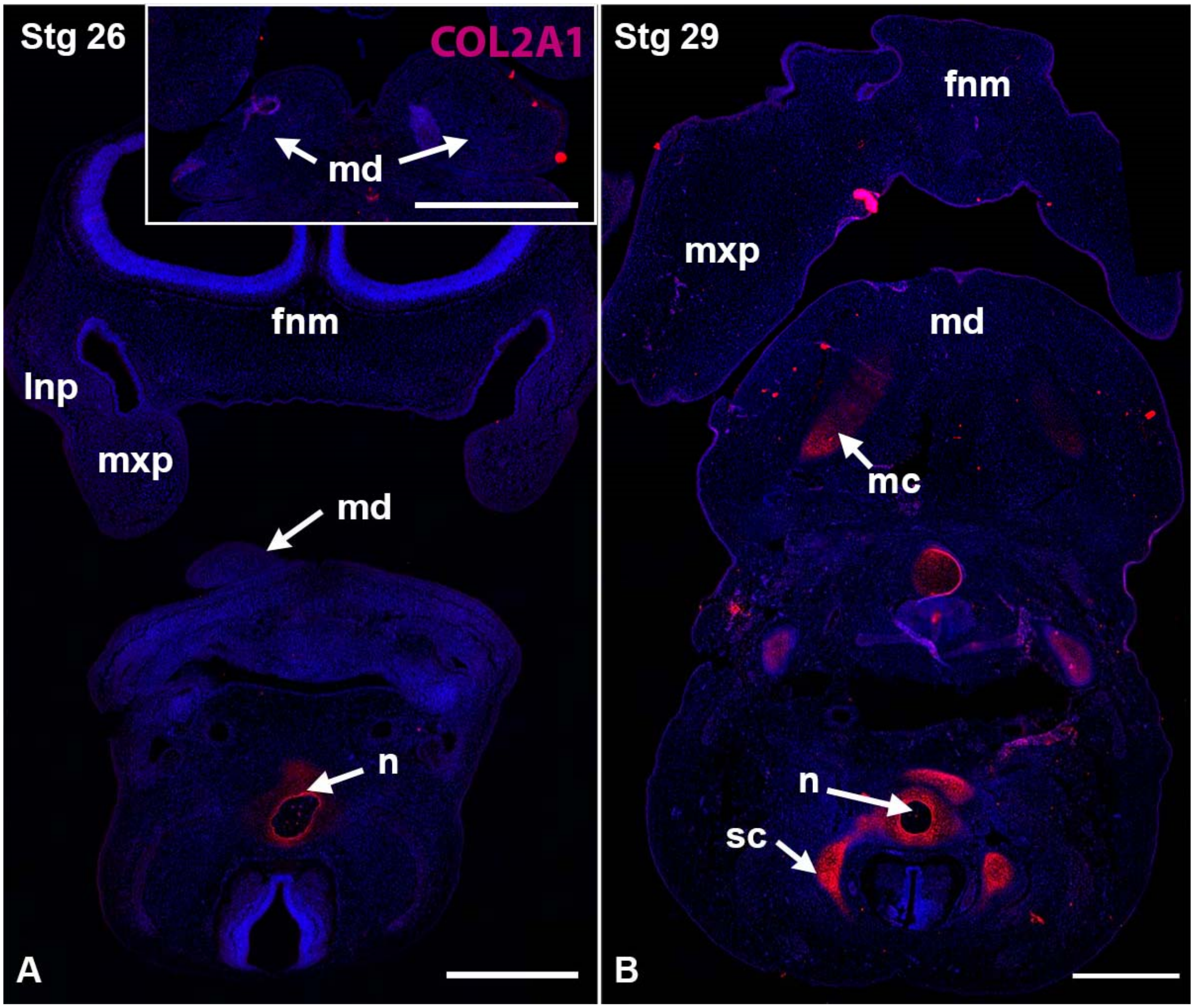

Figure S3. Immunostaining of the head of chicken embryos. Frontal sections cut through the frontonasal mass and mandible. A) At stage 26, midway between stage 24 and 28, there is no expression of COL2A1 (type II collagen) in the frontonasal mass or mandibular prominence (inset). The inset has some folds in the tissue but there is no signal for COL2A1. The only expression is seen in the notochord and surrounding mesenchyme. B) at stage 29 there is expression detected in Meckel's cartilage of the mandible, however there is no staining in the frontonasal mass. The mandible is differentiating prior to the frontonasal mass.
Key: fnm – frontonasal mass, lnp – lateral nasal prominence, mc – Meckel's cartilage, md – mandibular prominence, n – notochord, sc – sclerotome. Scale bar = 500 microns.

Development • Supplementary information

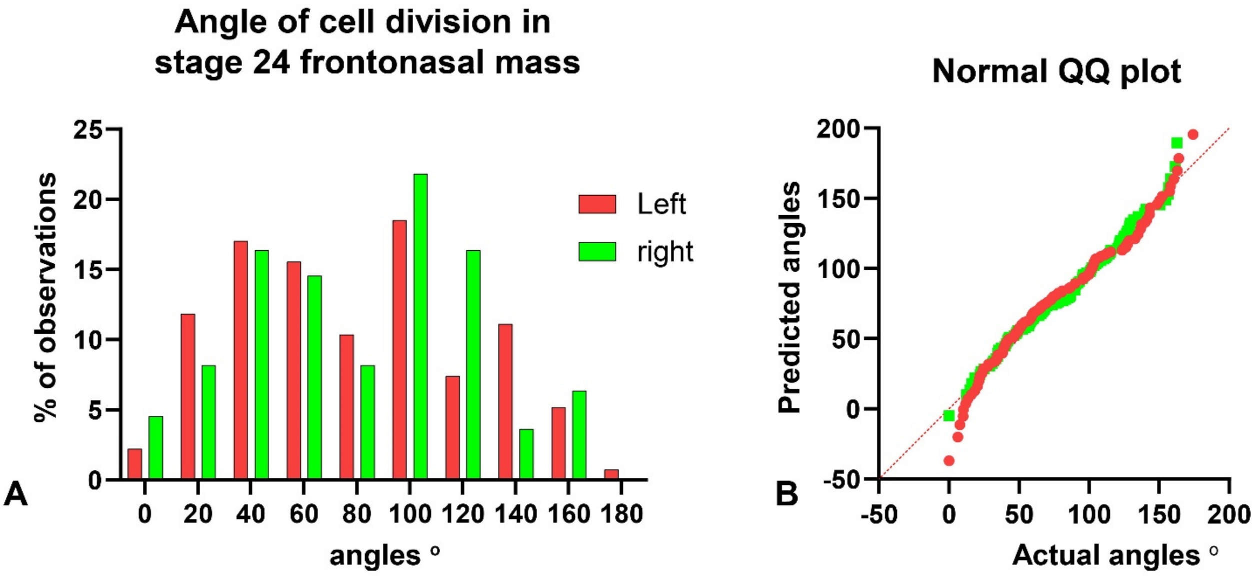

Figure S4. Frequency of observations of the angle of cell division when cells are in telophase. Cells were stained with pH3 antibody and angle of division was measured relative to the nasal slit. Zero and 180 degrees are parallel to the nasal slit. A) The observations in the left and right side are plotted separately. Generally there is no difference in the proportions of observations seen in the R and L sides. The exceptions are at 120 and 140 degrees. B) The overall normality of the data is not significantly different from predicted values.

Development • Supplementary information

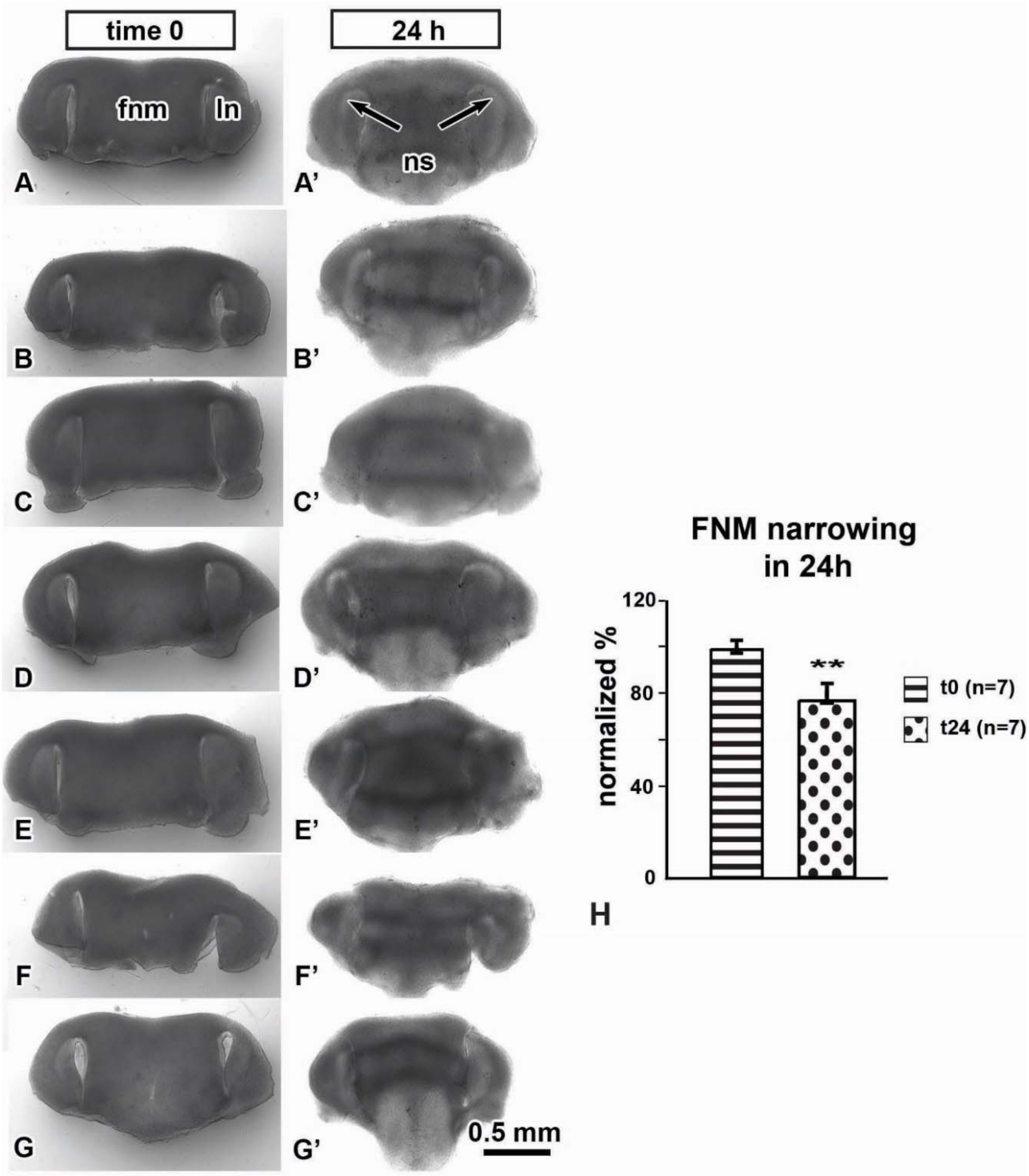

Figure S5. Images of frontonasal mass cultures before and after 24h of growth. A-G') The left column shows 7 individual cultures at the start of the growth period. The right column are the same cultures grown for 24h. H) Measurements of frontonasal mass width, midway through the nasal slit. Cultures were 20% narrower than at time zero.

Development • Supplementary information

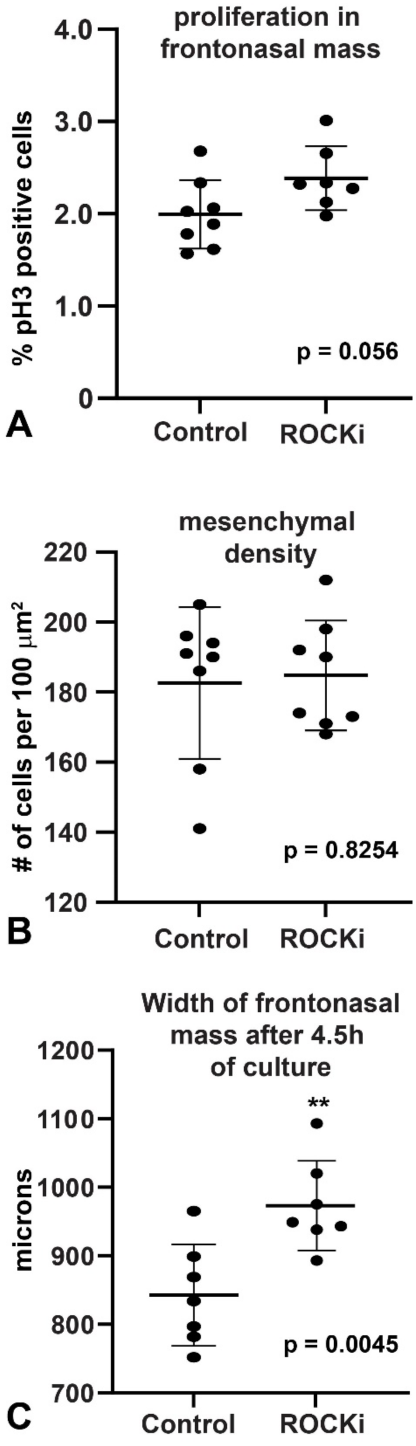

Figure S6. Analysis of frontonasal mass mesenchyme at the end of the imaging period. Cultures were grown in multi-well chamber slides on a layer of Matrigel for 4.5h, the period used for imaging. A) proliferation was assessed by counting the percentage of mitotic cells between the nasal slits. There was no significant difference between conditions. B) Cell density is unchanged by ROCKi treatment. C) Width of the frontonasal mass was measured at the midpoint of the nasal slits. There was a significant increase in width in the ROCKi treated cultures, similar to the results in 4G where cultures were measured after plating in Matrigel (took several hours). **=p< 0.01, determined by T-tests.

Development • Supplementary information

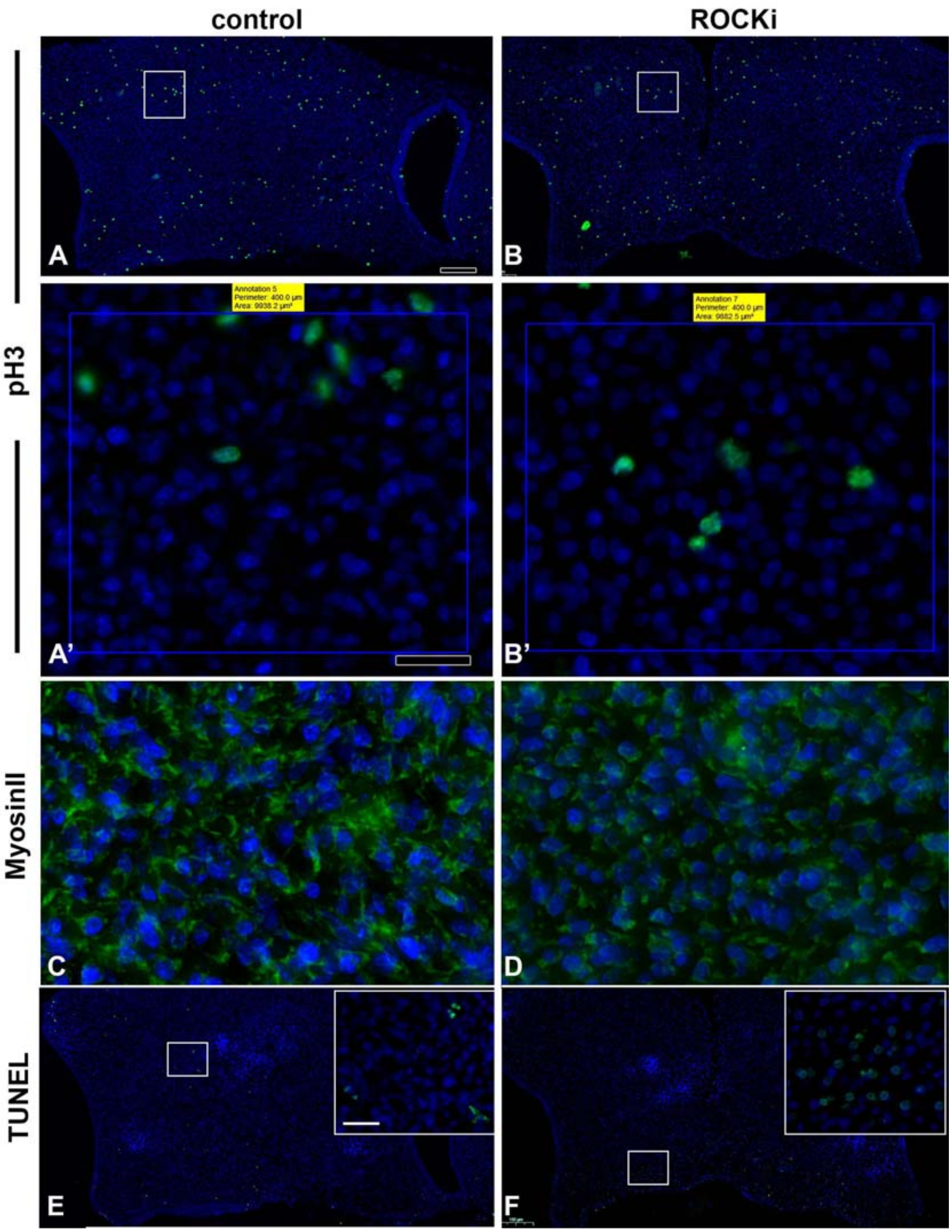

Figure S7. Frontonasal mass histology at the end of the imaging period. A, B) Staining of mitotic figures with pH3 was similar in control and treated cultures. A'B') an example of a 100 micron^2 area used to count cell density (inside the white box in A, B). C,D) Cytoskeletal staining with antibody to MyosinII non-muscle myosin. Cells are in contact with neighbouring cells and the matrix. E,F) Minimal detection of apoptotic cells in the control and treated cultures. Most of the signal is seen close the cut edges of the dissected frontonasl mass. Scale bars = 100 microns for A,B,E,F. Bar in A' = 20 microns and applies to B',C,D and insets in E, F.

Development • Supplementary information

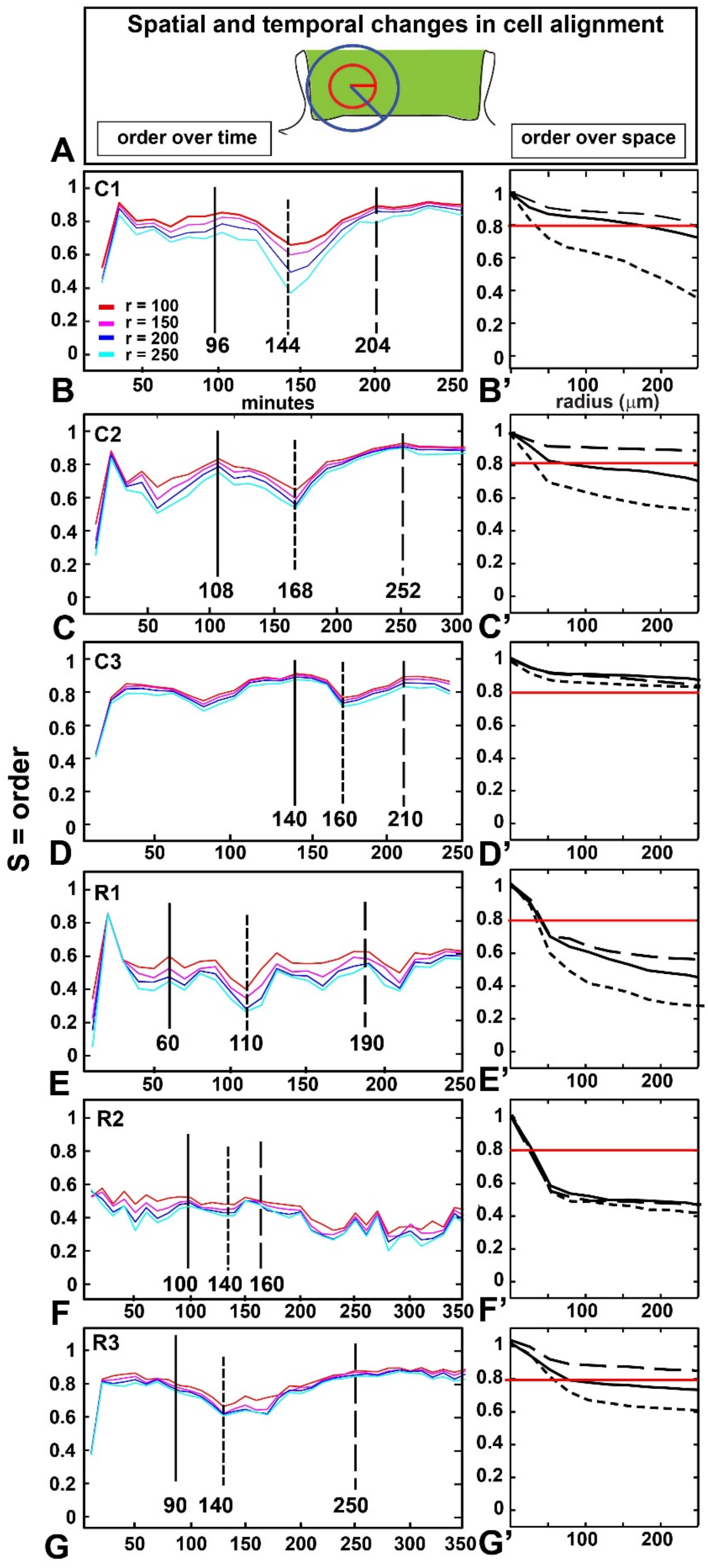

Figure S8. Spatial and temporal changes in cell alignment for all specimens at 10X: As in Fig 4H-I' but for the entire dataset. The direction of vectors at fixed time intervals was used to determine *S* as a function of time *t*. Vertical lines represent times of high directional order, disorder and order. Red circle is 100 μm in radius and blue circle is 200 μm radius. C1-C3 are controls and R1-R3 are ROCKi treated cultures.

Development • Supplementary information

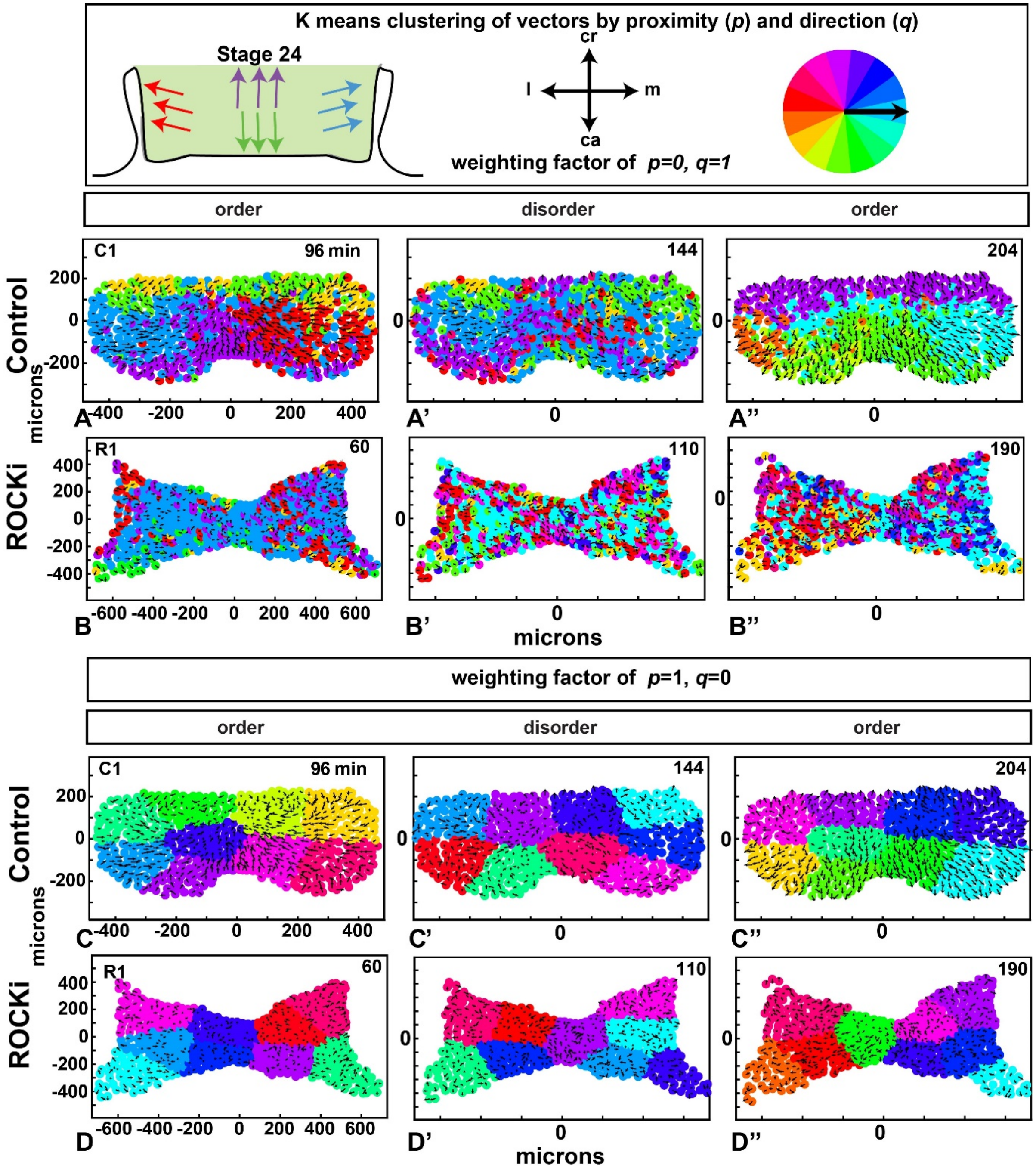

Figure S9. K-means clustering of vectors using Method 1 weighting on position and vector. A-B'') K-means clustering with relative weights position *p*=0, *q*=1) at the three time points of high order, disorder and order. The black arrows are cell velocity vectors. The colour wheel indicates the average direction of each cluster. When position is omitted and only vectors are considered the clusters are not distinct in controls. The ROCKi treated cultures have even less organization. **C,D''**) Here weighting is entirely on position and not on vector. The clusters are in blocks because proximity is prioritized.

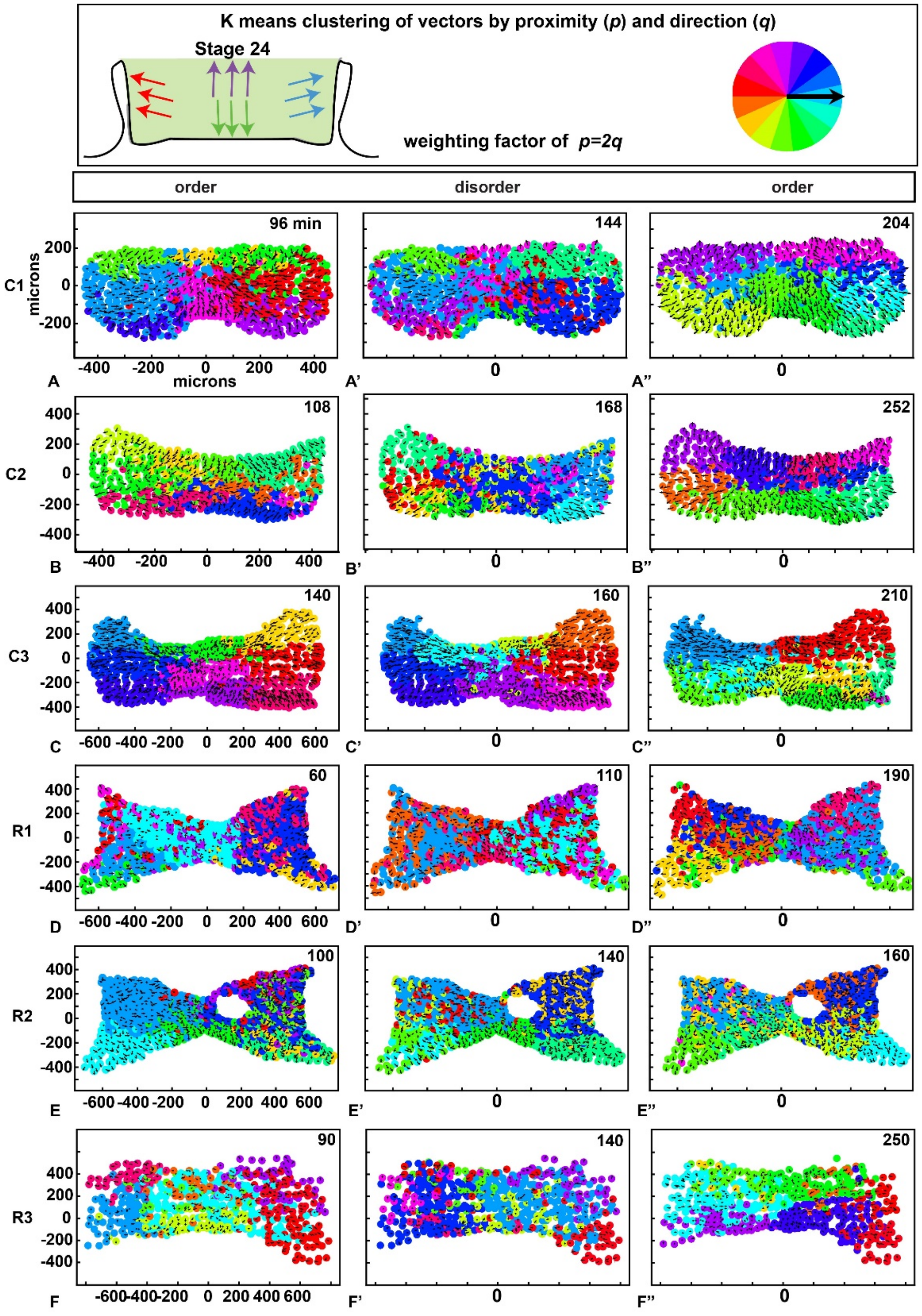

Figure S10. K-means clustering of vectors by Method 1. As in Fig 5A-B'', but for all specimens at 10X. K-means clustering with double the weighting for position compared to vector, p=2, q=1) at the three time points of high order, disorder and order (as in Supplementary Figure S5) The black arrows are cell velocity vectors. The colour wheel indicates the average direction of each cluster. **A-C'')** Clearer clusters are seen than if position was not considered. **D-F'')** Very few clear clusters can be seen in the ROCKi treated cultures.

Development • Supplementary information

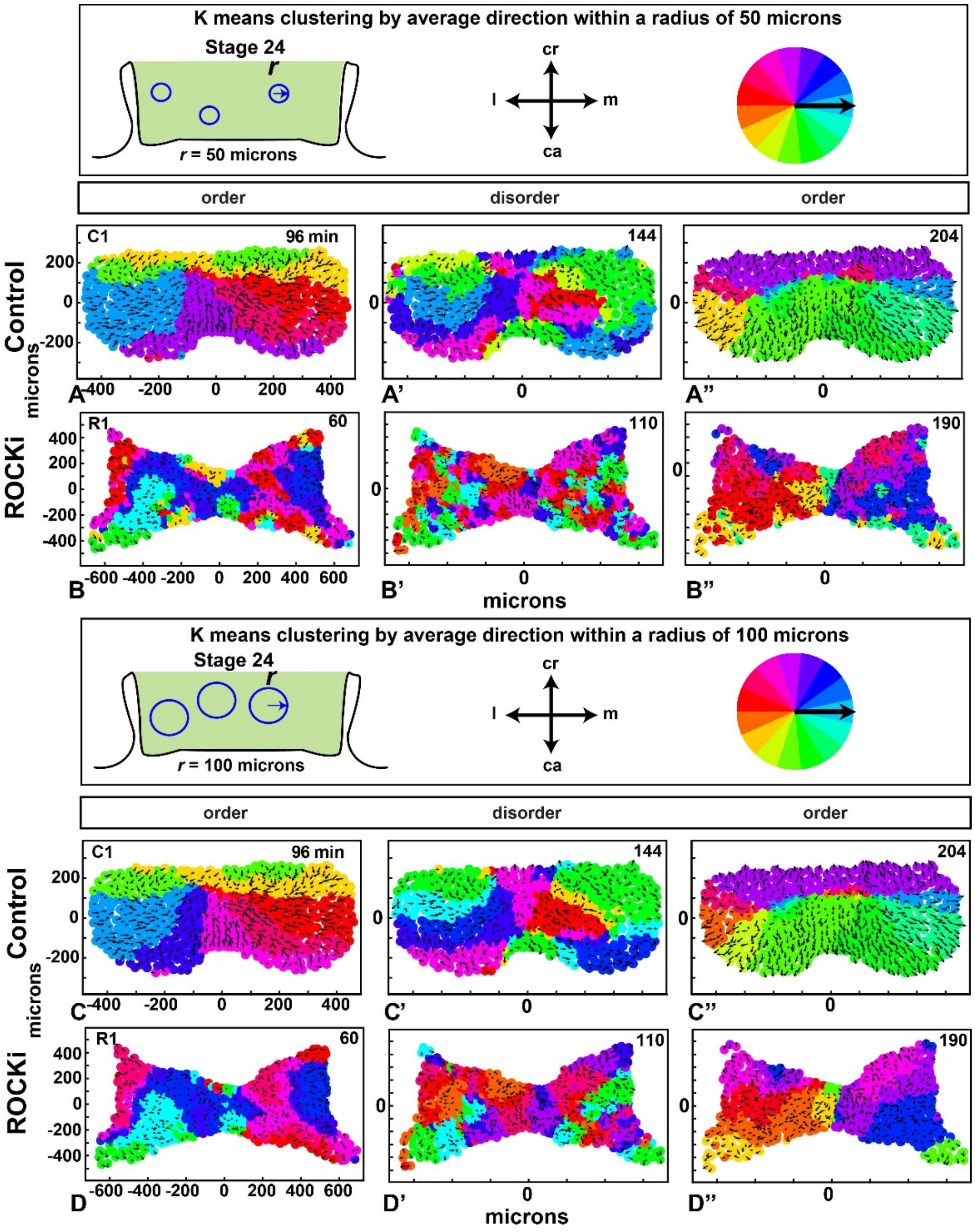

Figure S11. K-means clustering of vectors by Method 2 (cells positioned within the same circle of specific diameter). A-B'') Multiple circles of 50 micron radius were place across the frontonasal mass and clusters built on those regions. Clear clusters are seen in th first time point (A) and last (A''). No clear clusters are seen in the ROCKi treated cultures. C-C'') with a larger diameter circle clear clusters are seen in controls. D-D'') The ROCKi treated cultures have a patchwork of cells with the same angle that are positioned on opposite sides of the frontonasal mass. For example see fuchsia cells on both right and left sides of the frontonasal mass in D and D''.

Development • Supplementary information

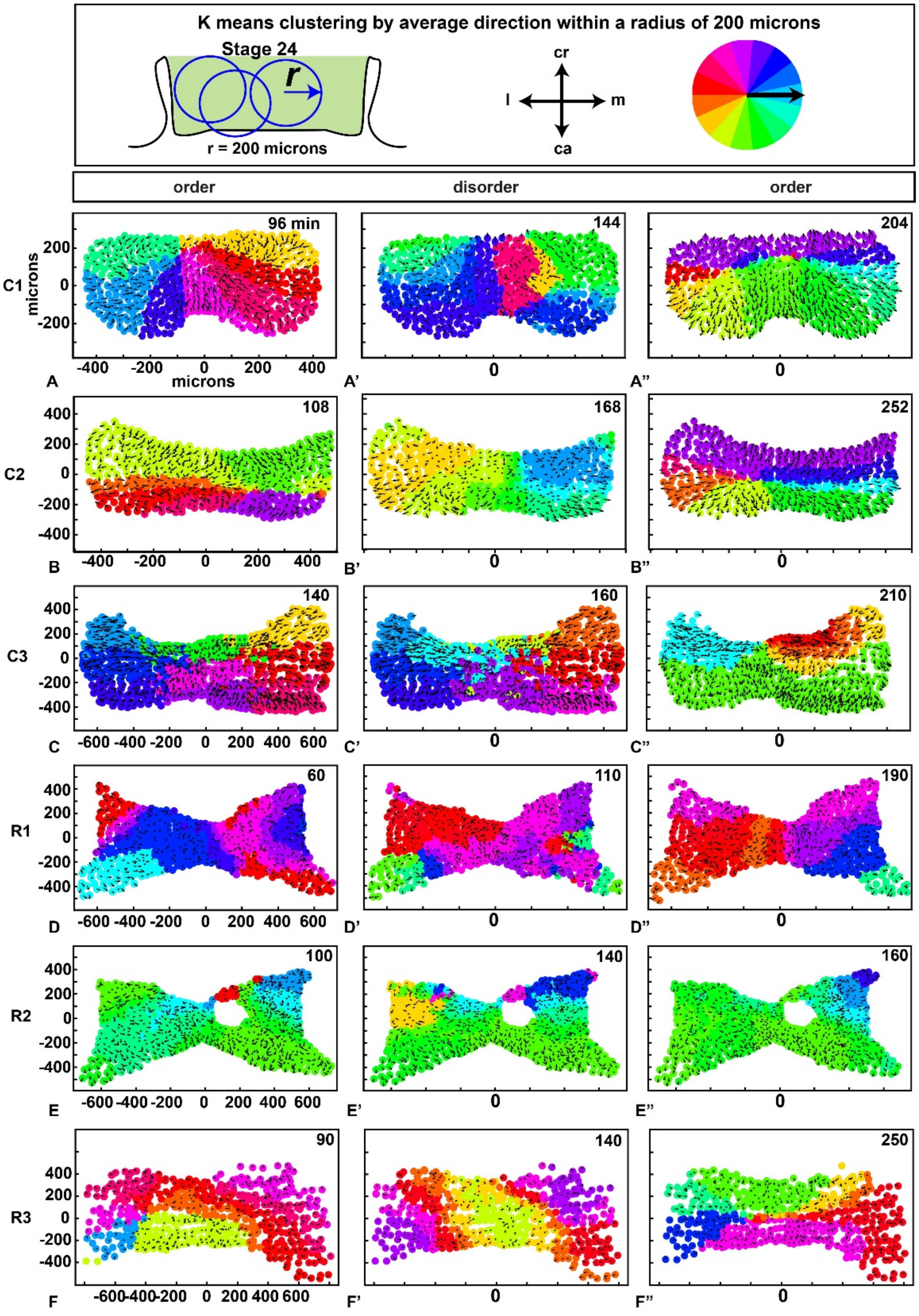

Figure S12. K-means clustering of vectors by Method 2. As in Fig 5C-D'', but for all specimens at 10X. K-means clustering by average direction (Method 2 with r = 200 µm) at the three time points of high order, disorder and order (as in Supplementary Figure S5) The black arrows are cell velocity vectors. Note that the colour wheel indicates the average direction of each cluster.

Development • Supplementary information

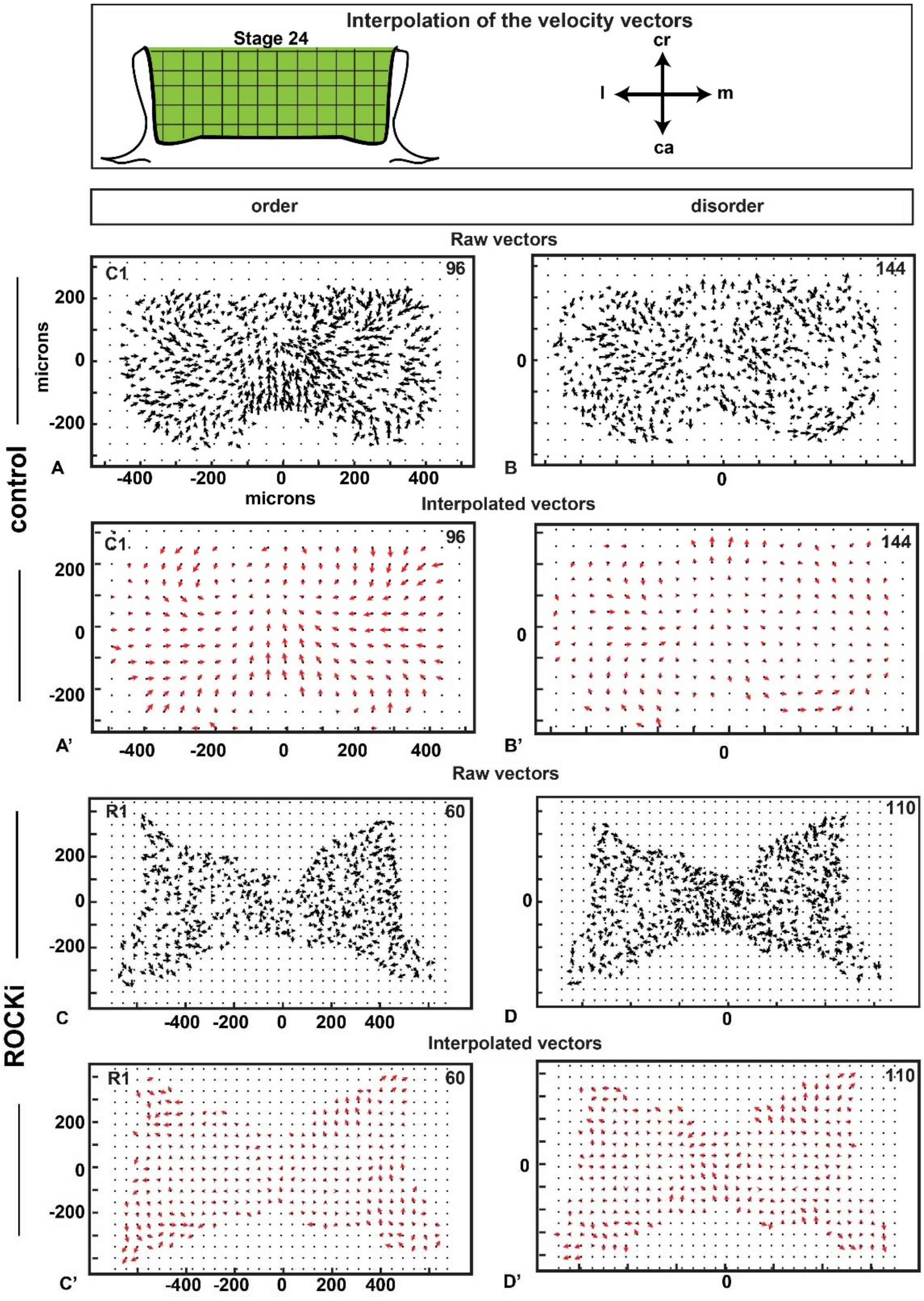

Figure S13. Interpolation or smoothing of data. Raw cell velocity vectors data (A,B,C,D in black) were interpolated by locally averaging data vectors over 50 µm radius neighborhoods. (Interpolation shown in red in A', B', C', D'). One control specimen (A-B') and one ROCKi treated specimen (C-D') are shown here, for ordered (A,A',C,C') and disordered states (B,B',D,D') Time-points in upper right corner, as in Fig 4H-I. The center of the frontonasal mass in controls tends to have smaller cellular velocities (shorter arrows). The ROCKi appears to have smaller velocities everywhere (slower cell motion).

Development • Supplementary information

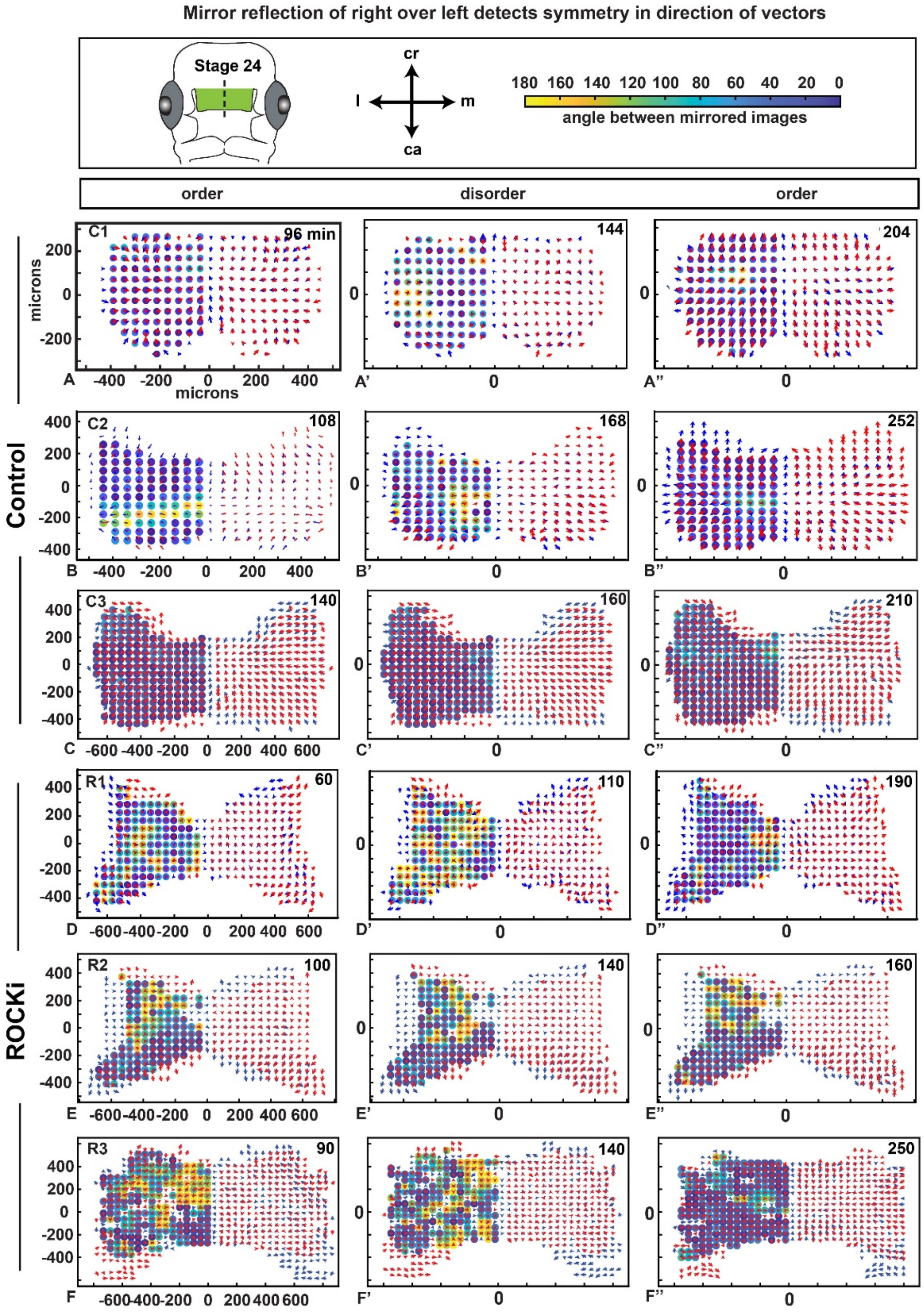

Figure S14. Right-left symmetry for all specimens.

Interpolated data (neighborhood averaging of 50 µm) at the three representative timepoints (Fig. S5) was used for the symmetry analyses. A-C'') Quantification of left-right symmetry in control cultures. There is high symmetry since the angle between mirrored images is very similar. D-F'') In ROCKi treated cultures there are mare areas with yellow circles or low symmetry, especially in the period of disorder.

Development • Supplementary information

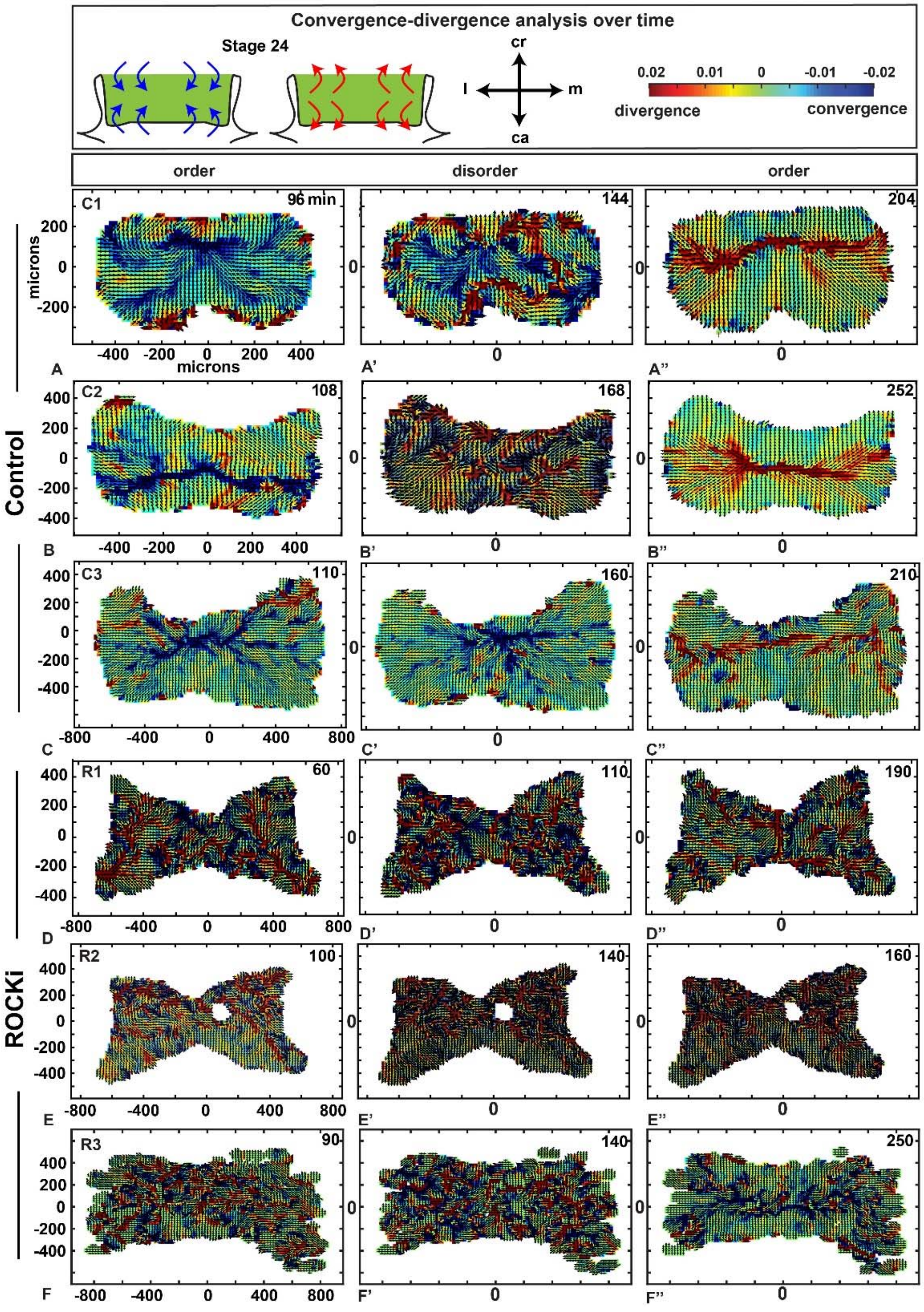

Figure S15. Convergence-divergence analysis for all specimens

A-C'') Control cultures have tendency for convergence towards the midline of the culture at the first period of order, but this changes to divergence by the end of the culture period. D-F'') In ROCKI treated cultures there is no clear pattern of divergence and convergence at any of the time points.

Development • Supplementary information

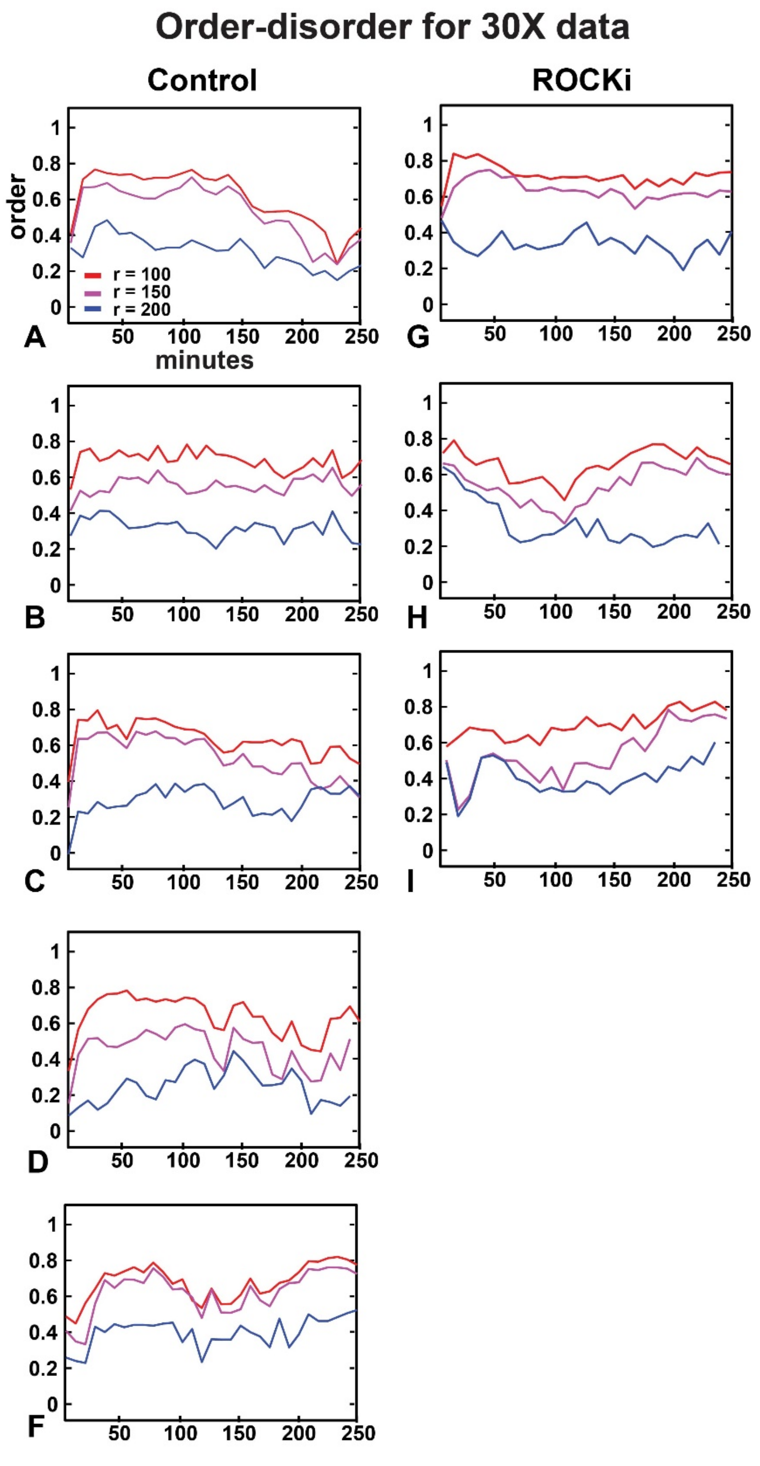

Figure S16. Order parameter over time at 30X. Time plots of the order parameter for the 30X data computed over various neighbourhoods (r = 100 - 500 µm). In contrast to Fig S5, no clear periodicity is seen in these graphs, demonstrating the fact that 10X data reveals trends not seen at higher magnification.

Development • Supplementary information

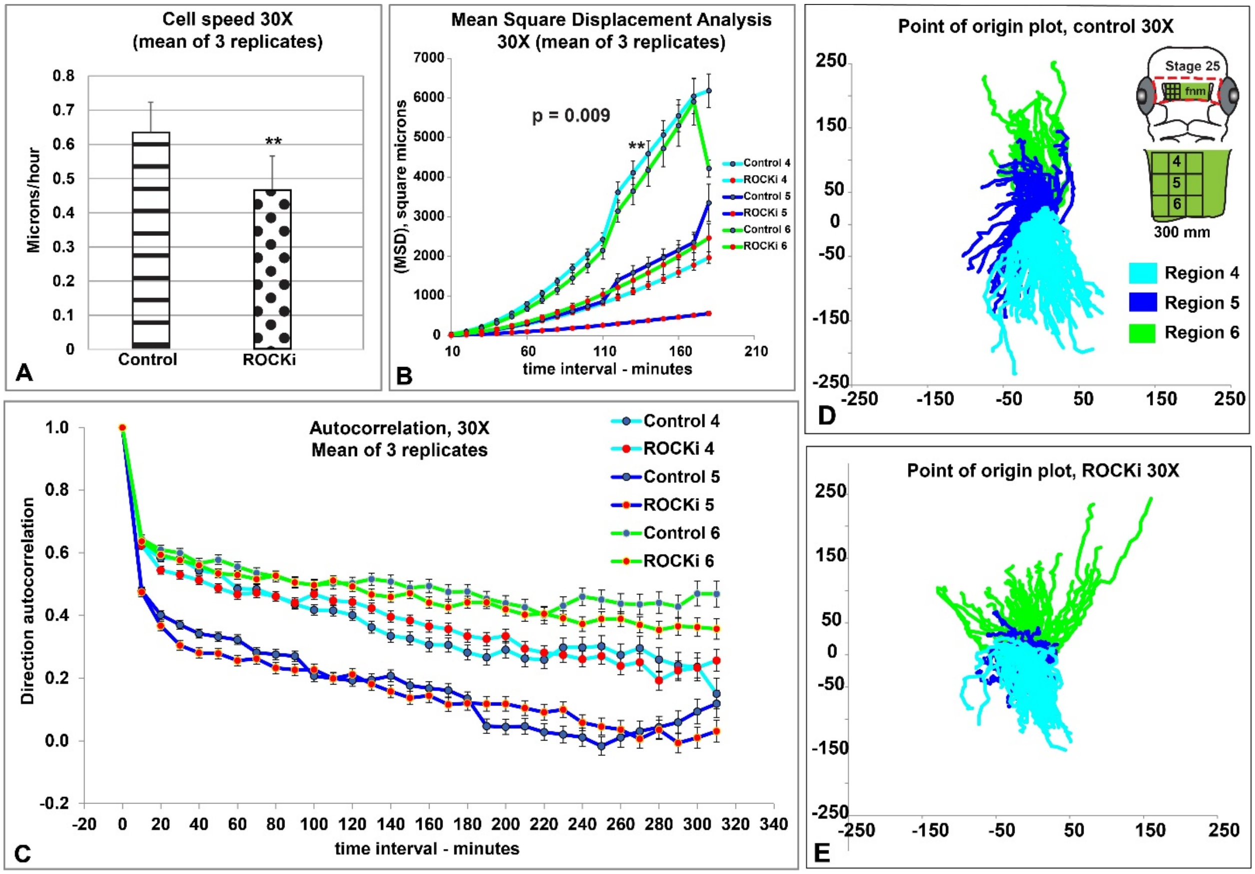

Figure S17. Individual cell measurements in the frontonasal mass.
The lateral third of the frontonasal mass was imaged. A grid was placed over the region and divided into 9 regions. Data from regions 4,5,6 is depicted and this data is representative of the other regions. Region 4 is at the cranial edge of the frontonasal mass, Region 5 is in the centre of the frontonasal mass, region 6 is close to the caudal edge. **A)** Instantaneous cell speed across all timepoints. Speeds for each culture were averaged and were considered 1 replicate. ROCKi treatment significantly decreased average cell speed. **B)** Displacement of cells in the controls was greater than for the equivalent region of the frontonasal mass in ROCKi treated cultures. P value is the result of ANOVA analysis comparing treated and control data. Regions are depicted in panel D. **D,E**) All cell tracks from the three replicates placed on a single point of origin. Axes are arbitrary. **D)** The tendency is for polarization of the cranial and caudal tracks (4,6) whether the centre has tracks that go cranial and caudally. **E)** The ROCK treatment causes more mediolateral spread. Also the tracks do not extend as far on the Y axis. Region 5 shows radial tracks that are relatively short.

Development • Supplementary information

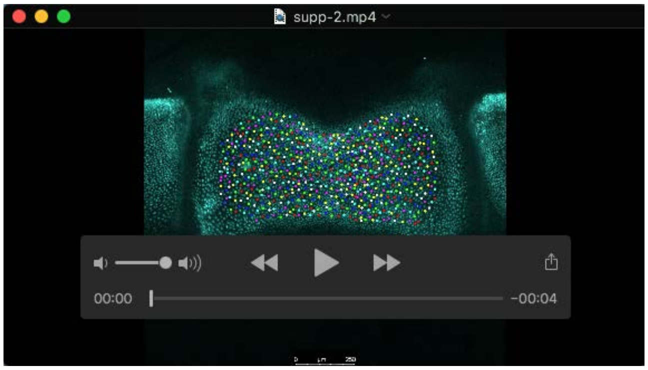

Movie 1. Time-lapse of stage 25 frontonasal mass dissected and cultured in control media. Nuclei were stained with Hoechst dye and imaged with confocal microscope, at 10X magnification (C1, control organ culture). Individual nuclei were tracked with manual tracking software. Dot is the end of the track and the tail is the trajectory of the movement. Cultures are oriented with the cranial edge uppermost in the image. The nasal slit is on the lateral sides of the tissue. The center of the frontonasal mass is relatively stationary in the center where cartilage will form at later stages.

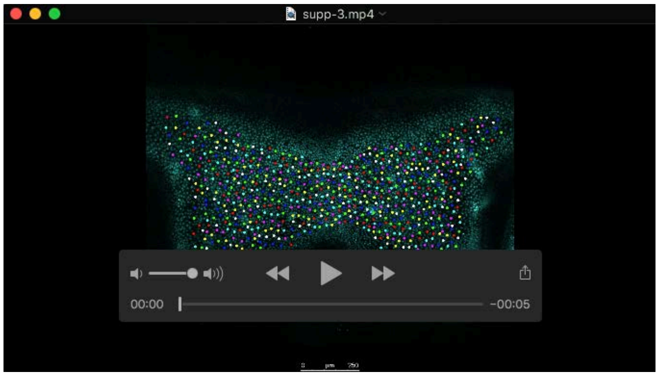

Movie 2. Time-lapse of stage 25 frontonasal mass dissected and cultured in ROCKi treated media. Nuclei were stained with Hoechst dye and imaged with confocal microscope, at 10X magnification (R1, ROCK inhibitor treatment organ culture). Tracks appear more disorganized and tissue failed to narrow in the mediolateral axis. The center of the frontonasal mass is relatively stationary in the center where cartilage will form at later stages, similar to control tissues.

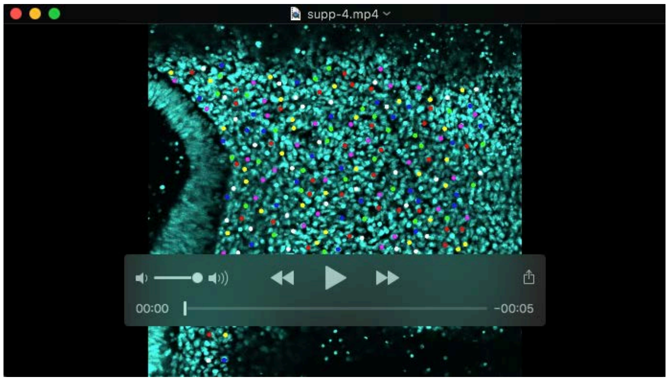

Movie 3. Time-lapse of stage 25 frontonasal mass dissected and cultured in control media. Nuclei were stained with Hoechst dye and imaged with confocal microscope, at 20X magnification with 1.5 optical zoom (C4, control organ culture).

Development • Supplementary information

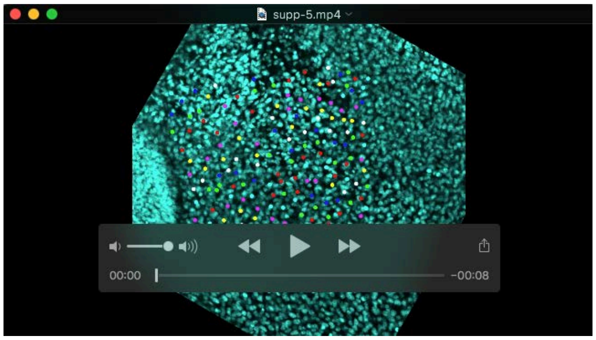

Movie 4. Time-lapse of stage 25 frontonasal mass dissected and cultured in ROCKi-treated media. Nuclei were stained with Hoechst dye and imaged with confocal microscope, at 20X magnification with 1.5 optical zoom (R4, organ culture).

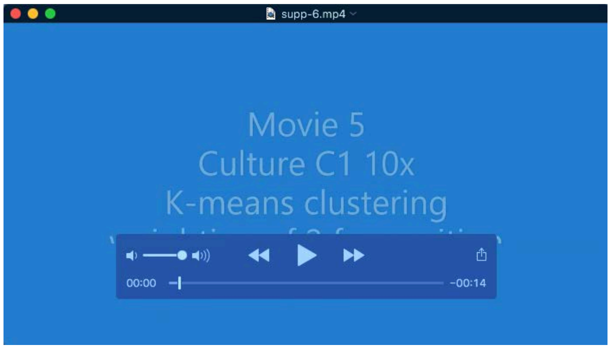

Movie 5. Time-lapse of K-means clustering by Method 1 (by cell position and direction, relative weights p =2, q =1) for C1, control organ culture. Clusters are spatially diffuse.

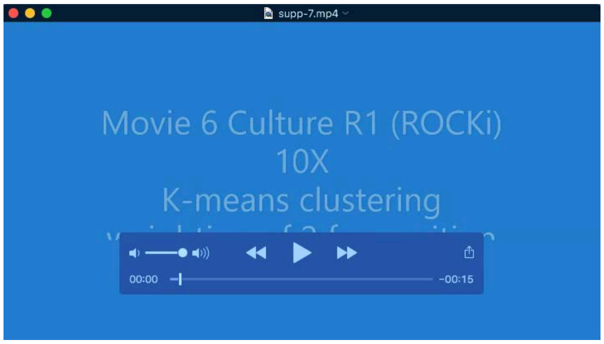

Movie 6. As in Movie 5 but for the R1, ROCK inhibitor treatment organ culture. Clusters are even more spatially diffuse than in the controls.

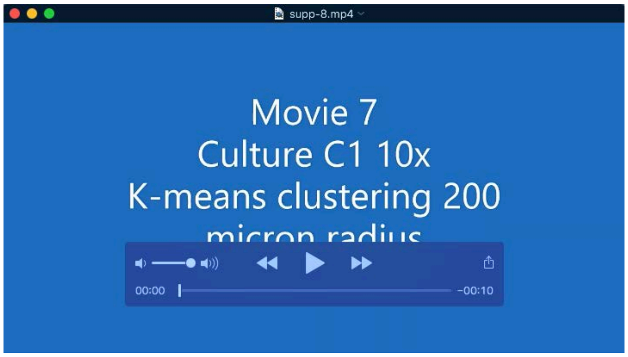

Movie 7. Time-lapse of K-means clustering by Method 2 (considering only mean cell directions over a 200 µm radius) for C1 control organ culture. The maximum number of clusters is 7. The black arrows are the raw data for a given cell. Clusters of cells on the right and left sides and cranial-caudal axes are moving in opposite directions (see colour wheel) during the ordered times.

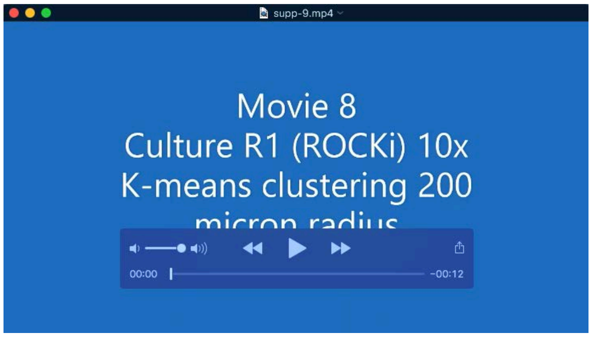

Movie 8. As in Movie 7. But for R1, ROCK inhibitor treatment organ culture. The maximum number of clusters is 7. The black arrows are the raw data for a given cell. In contrast to control samples, the ROCKi clusters are heterogeneous, lack neighbour similarity and are not organized into distinct regions. Clusters of cells with the same vector are not located in contiguous regions.

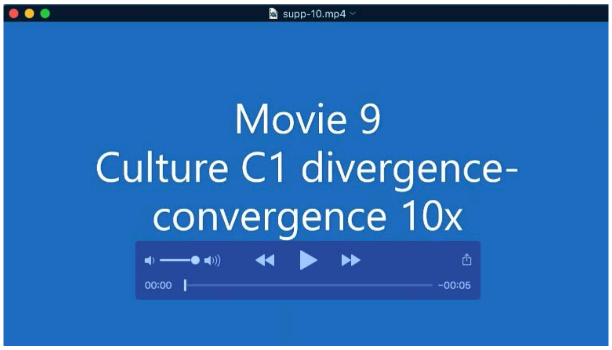

Movie 9. Time-lapse for divergence and convergence in the frontonasal mass for C1, control organ culture. Raw vector data was interpolated (averaged over 20 µm radius neighborhood) to facilitate the observation of complex patterns of divergence and convergence. We observed an initial band of convergence with several prominent branches at the lateral edges of the frontonasal mass followed by an overall direction change to divergence.

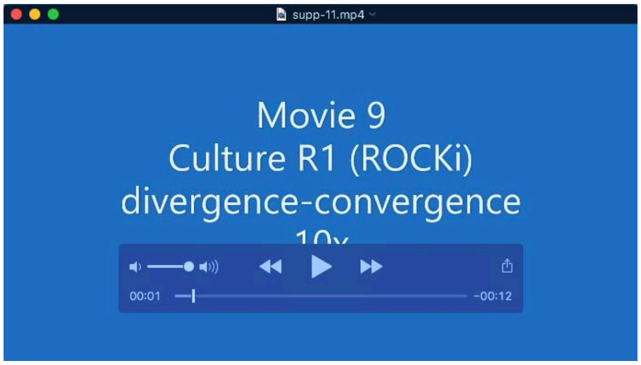

Movie 10. As in Movie 9 but for R1, ROCK inhibitor treatment organ culture. ROCKi treatment disrupted the patterns of convergence and divergence throughout the observations.

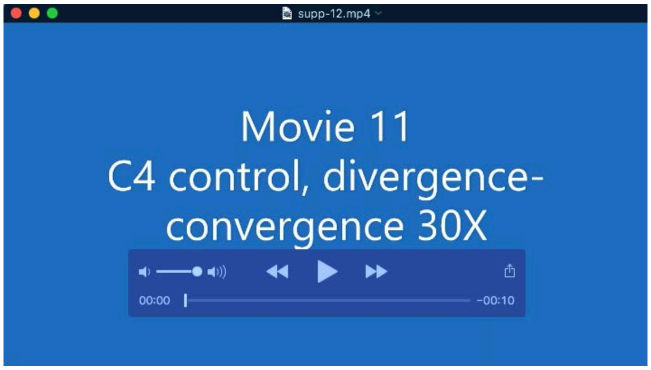

Movie 11.

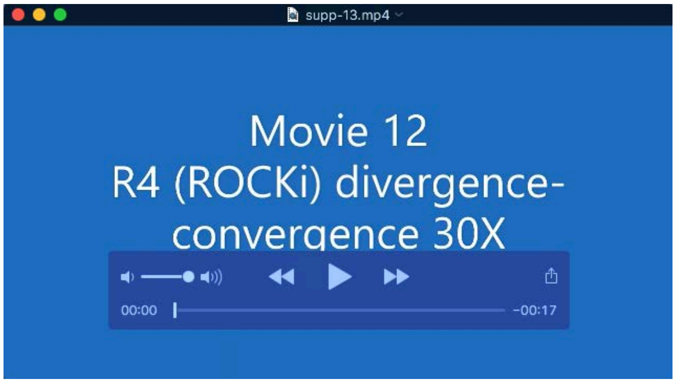

Movie 12.

Development • Supplementary information

Supplementary Materials and Methods

OVERVIEW OF CELL TRACKING DATA ANALYSIS

ELISABETH RENS

Department of Mathematics, University of British Columbia, Vancouver, Canada

LEAH EDELSTEIN-KESHET

Department of Mathematics, University of British Columbia, Vancouver, Canada

Calculating velocity. The tracking data provides us with an x and y coordinate for each cell. Let i denote the cell number and $\mathbf{x}_i(t) = (x_i(t), y_i(t))$ its coordinates at time t. Then we approximate the velocity vector $\mathbf{v}_i(t) = (u_i(t), v_i(t))$ as follows:

$$u_i(t) = \frac{x_i(t) - x_i(t - \Delta t)}{\Delta t} \quad (1)$$

$$v_i(t) = \frac{y_i(t) - y_i(t - \Delta t)}{\Delta t} \quad (2)$$

where Δt=10 minutes, the time between each image.

Right-left symmetry. Here we examine the left-right (reflective) symmetry in the velocity field across the vertical midline of the samples (defined below). To better quantify symmetry, we first interpolate the velocity vectors to a fixed grid. This allows us to directly compare velocities at corresponding gridpoints. We defined a fixed grid with spacing $\Delta x = \Delta y = 50\mu$m. For each grid point, we then calculate the average ($\mathbf{v}_a$) of the n velocity vectors ($\mathbf{v}_i$) that are within a radius of 50μm.

These interpolated velocity vectors were then mirrored along the vertical midline. We define the mirroring axis for each specimen as $x = \frac{x_{\max}+x_{\min}}{2}$. Here, $x_{\max} = \max_{i,t} x_i(t)$ is the maximum coordinate of all cells over all time points in that specimen and similarly for the minimum coordinate. The mirrored velocities are defined as

$$\mathbf{v}_i^{\mathrm{mirr}}(t) = (-u_i(t), v_i(t))$$

Now at each gridpoint, we calculate the difference in angle between $\mathbf{v}_i(t)$ and $\mathbf{v}_i^{\mathrm{mirr}}(t)$ as $\alpha_i - \alpha_i^{\mathrm{mirr}}$.

Development • Supplementary information

Development: doi:10.1242/dev.193755: Supplementary information

Divergence fields. To identify sources and sinks of movement, we calculate the divergence field. The divergence is defined as

$$\text{div } \mathbf{v}_i(t) = \nabla \cdot \mathbf{v}_i(t) = \frac{\partial u_i(t)}{\partial x} + \frac{\partial v_i(t)}{\partial y}$$

Negative divergence indicate a sink and positive divergence indicates a source. To approximate the spatial derivatives, we use a centered finite difference approximation. For cells at the boundary of the tissue, we use a forward/backward finite difference approximation. To be able to calculate the spatial derivatives of the velocity vectors sufficiently accurate, we first interpolated the velocity vectors on a much finer grid with spacing $\Delta x = \Delta y = 20\mu m$. At each gridpoint, we calculate the average velocity of the velocity vectors within a radius of 50 μ m of this gridpoint, again using equation **??**. We then calculated the divergence for this interpolated velocity field. Next, we also calculated the divergence for the normalized interpolated velocities. In the normalized case, all velocity vectors have length one, so that only the direction of motion is taken into account in calculating the divergence. For clarity, we show both the divergence of the interpolated velocity field and of the normalized field (unit vectors in the directions of the velocities).

The divergence field of the normalized vectors shows clear bands of divergence/convergence, that are usually more or less horizontal. We analyzed the divergence over time, by quantifying the divergence in these bands. First, we quantitatively identified the position of these bands as follows. We looked for a band of width r=60μm that had the most negative compared to positive divergence values, or the most positive compared to negative divergence values. Let $N_d(y)$ and $N_c(y)$ be defined as the number of grid points in the band around y that are either negative (diverging) or positive (converging):

$$N_d(y) = \#(x, y) : div(x, y - r \leq y \leq y + r) < 0$$

$$N_c(y) = \#(x, y) : div(x, y - r \leq y \leq y + r) > 0$$

Now the center of the band is found as

$$Y = \max_y \frac{\max\left(N_c(y), N_d(y)\right)}{\min\left(N_c(y), N_d(y)\right)}.$$

We imposed that the band should have a sufficient number of data points. This ensures that the band will not fall in the complete top or bottom of the tissue where not many data points are present. Let N be defined as

$$N = \max_y \#\text{gridpoints}(y)$$

the maximum number of data points existing in a row of the interpolated grid. We imposed that the number of data points within the band should be at least $0.7 \cdot N \cdot (\frac{2r}{20} + 1)$. A visual check confirmed that this simple algorithm identifies the location of the bands well. Next, we computed the average divergence over all data points in the located band:

$$\overline{div} =< div(x, Y - r \leq Y \leq Y + r) >,$$

Development • Supplementary information

where <> denotes the average. We used this measure to evaluate divergence over time.

Range of velocity alignment. We borrow the concept of an 'order parameter' from physics of liquid crystals [3], that has also been used in computational biology to quantify the alignment of cell shapes [5, 6, 8]. Here, we use this concept to quantify the extent of local alignment (of velocity vectors). We calculate the average velocity vector $\mathbf{v}_a^r(t)$ in a circular region with radius r around each cell i to obtain $\mathbf{n}_i^r(t)$ called the 'local director', which describes a local average direction within a range r of the cell i. The angle between this local director and cell i is denoted $\alpha_i^r(t)$. Now our 2D order parameter is calculated as

$$S(r,t) = \langle \cos(\alpha_i^r(t)) \rangle_i$$

If all cells are moving in exactly the same direction, then S will be close to 1. If cells move in various random directions, S will be close to zero. For a small r, S quantifies velocity alignment for cells close to each other, while for very large $r \approx 500\mu$m, S quantifies velocity alignment over the whole tissue. This 2D order parameter used here is slightly adapted from its original form ($S(r) = \langle \cos(2\alpha_i^r) \rangle_i$). In the alignment of molecules (physics) or cell shape (biology), a factor 2 is used because the agents do not have a direction, only an orientation (i.e. a direction of 0° is equivalent to 180°). Since we are studying velocities, direction is relevant to us, so we omit the factor 2. This can lead to negative values of S, because if a cell is moving in the opposite direction to all neighbouring cells, its $\alpha_i^r(t)$ will be 180° and thus $\cos(\alpha_i^r(t)) = -1$. Note that this order parameter, has been used to quantify velocity correlations over space before in [9, 7, 2].

Clustering. We use a weighted k-means clustering algorithm [1] to find regions of the tissue in which cells move similarly. The k-means clustering algorithm clusters objects based on the similarity between the variables of the objects, while selecting an optimal number of clusters. The algorithm clusters the objects by minimizing the 'distances' from objects to the 'centroid' of the clusters. The distance between an object and the centroid is defined as the sum over the Euclidean distance between their variables. In a weighted k-means clustering, the centroid of a cluster is weighted, i.e. the centroids of the cluster i are given by

$$Cij = \frac{\sum_k w_k V_{jk}}{\sum_k w_k}, \tag{3}$$

where k runs over all objects in the cluster, Vjk is the jth variable of the kth object and w_k the weight of object k. So, objects with higher weights are given more importance to the identity of the cluster.

In our case, the objects are cells and we want to cluster the cells based on similarity in velocity and position. We experimented with the following choices for variables and weights: 1) variables: direction of velocity and

Development • Supplementary information

Development: doi:10.1242/dev.193755: Supplementary information

positions of cells, weight: velocity of cells, and 2) variable: the 'local director' as described above with weight given by the local order. The details of the two different methods are described below. To avoid effects of noise and small artifacts, we restruct the number of clusters to no 9 or fewer. The algorithm usually gave an optimal number of clusters between 6 and 9.

Clustering angles and positions. To cluster cells based on their directions and positions, we define the following four variables V_1, V_2, V_3, V_4:

$$V_1 = q \cdot \frac{1+\cos(\alpha)}{2} \quad \text{(normalized velocity in x)} \tag{4}$$

$$V_2 = q \cdot \frac{1+\sin(\alpha)}{2} \quad \text{(normalized velocity in y)} \tag{5}$$

$$V_3 = p \cdot \frac{x - \min_i x_i(t)}{\max_i x_i(t) - \min_i x_i(t)} \quad \text{(normalized x coordinate)} \tag{6}$$

$$V_4 = p \cdot \frac{y - \min_i y_i(t)}{\max_i y_i(t) - \min_i y_i(t)} \quad \text{(normalized y coordinate)} \tag{7}$$

describing the position and directions of cells. The normalization ensures equal weighting of all variables ($0 \leq C1, C2, C3, C4 \leq 1$) if $p = 1$ and $q = 1$. We set $q = 1$. By varying p, we can assign more or less weight to the positions in comparison to the directions of the cells. For small p, the clustering will be mainly done on the angles, so that cells far apart can be clustered together. For larger p, cells are less likely to become clustered together if they are spatially far apart. Finally, the weights are given by $w_i = |\mathbf{v}_i(t)|$. This ensures that cells with higher velocities are assigned more confidence (data of cells with low velocities is more prone to errors).

We experimented with a variety of clustering options, by (1) varying p, (2) clustering only V_1 and V_2 (equivalent to $p = 0$), (3) clustering only V_3 and V_4 (q=0,p=1). We compared our clusters of large p ($p = 2, p = 5$) to case (3), to ensure that V_1, V_2 are at least given some weight and the clustering in not too biased towards positions. We found that $p = 2$ gives the best results. Directions are well clustered and, at the same time, position is taken into account, so that the clusters are connected in space.

Clustering the local director. Instead of clustering the direction and positions, here we cluster the local average orientation of cells given by the local director $\mathbf{n}_i^r(t)$ as described before. Let's call this local orientation γ. Now we cluster two variables V_1 and V_2 given by

$$V_1 = \frac{1+\cos(\gamma)}{2} \quad \text{(normalized velocity in x of local director)} \tag{8}$$

$$V_2 = \frac{1+\sin(\gamma)}{2} \quad \text{(normalized velocity in y of local director)} \tag{9}$$

Development • Supplementary information

Here the weights are given by

$$w_i = \frac{1 + \cos(\alpha_i^r(t))}{2}$$

where $0 \leq w_i \leq 1$ and $\alpha_i^r(t)$ is the angle between the local director and cell i. If w_i is large, this means that the direction of the cell is similar to the average orientation and thus will be given a higher confidence.

By varying r in this clustering method, we basically smooth the velocity vector field over a range r. In contrast to the first method, this clustering method does not take the positions of cells into account explicitely. However, this 'smoothing' ensures that two cells far apart, but moving in a similar direction, will not be clustered together, because the average direction within these two different regions are different. We performed this clustering method for $r = 0, 50, 100, 200, 300, 400\mu$m. If $r = 0$, then the local director is the same as the direction of the cell and $w = 1$ for all cells. This means we are clustering the original directions of the cell, and this case is equivalent to our first clustering method with $p = 0$. We found that $r = 200$ gives good results, as it is a biologically relevant [4, 10] and the clusters are connected in space.

References

[1] Margareta Ackerman, Shai Ben-David, Simina Brânzei, and David Loker. Weighted clustering. In *AAAI*, 2012.

[2] Thomas E Angelini, Edouard Hannezo, Xavier Trepat, Jeffrey J Fredberg, and David A Weitz. Cell migration driven by cooperative substrate deformation patterns. *Physical review letters*, 104(16):168104, 2010.

[3] PG de Gennes. J. prost, the physics of. *Liquid Crystals*, 1993.

[4] Norihisa Higashihori, Marcela Buchtová, and Joy M Richman. The function and regulation of tbx22 in avian frontonasal morphogenesis. *Developmental Dynamics*, 239(2):458–473, 2010.

[5] Ralf Kemkemer, Simon Jungbauer, Dieter Kaufmann, and Hans Gruler. Cell orientation by a microgrooved substrate can be predicted by automatic control theory. *Biophysical journal*, 90(12):4701–4711, 2006.

[6] Margriet M Palm and Roeland MH Merks. Vascular networks due to dynamically arrested crystalline ordering of elongated cells. *Physical Review E*, 87(1):012725, 2013.

[7] Laurence Petitjean, Myriam Reffay, Erwan Grasland-Mongrain, Mathieu Poujade, Benoıt Ladoux, Axel Buguin, and Pascal Silberzan. Velocity fields in a collectively migrating epithelium. *Biophysical journal*, 98(9):1790–1800, 2010.

[8] Elisabeth G Rens and Roeland M H Merks. Cell contractility facilitates alignment of cells and tissues to static uniaxial stretch. *Biophysical Journal*, 112(4):755–766, 2017.

[9] Benjamin Slater, Camila Londono, and Alison P McGuigan. An algorithm to quantify correlated collective cell migration behavior. *Biotechniques*, 54(2):87–92, 2013.

[10] Heather L Szabo-Rogers, Poongodi Geetha-Loganathan, Suresh Nimmagadda, Kathy K Fu, and Joy M Richman. Fgf signals from the nasal pit are necessary for normal facial morphogenesis. *Developmental biology*, 318(2):289–302, 2008.

Development • Supplementary information
