# Supplementary material for: Symmetry and fluctuation of cell movements in neural crest-derived facial mesenchyme
Source: Development. 2021 May 7;148(9):dev193755. doi: 10.1242/dev.193755 (PMC8126411; doi:10.1242/dev.193755)
Supplement: Reviewer comments [file develop-148-193755_review_history.pdf]

Symmetry and fluctuation of cell movements in neural crest-derived facial mesenchyme

Adrian Danescu, Elisabeth G. Rens, Jaspreet Rekhi, Johnathan Woo, Takashi Akazawa, Katherine Fu, Leah Edelstein-Keshet and Joy M. Richman
DOI: 10.1242/dev.193755

Editor: Paul François

Review timeline
Original submission: 12 June 2020
Editorial decision: 25 January 2021
First revision received: 3 February 2021
Accepted: 5 March 2021

Original submission

<u>First decision letter</u>

MS ID#: DEVELOP/2020/193755

MS TITLE: Symmetry and coordination of cell movements in neural crest-derived facial mesenchyme

AUTHORS: Adrian Danescu, Elisabeth G. Rens, Jaspreet Rekhi, Johnathan Woo, Takashi Akazawa, Katherine Fu, Leah Edelstein-Keshet, and Joy Richman

I have now received all the referees' reports on the above manuscript, and have reached a decision. The referees' comments are appended below, or you can access them online: please go to BenchPress and click on the 'Manuscripts with Decisions' queue in the Author Area.

As you will see, the referees express considerable interest in your work, but have some significant criticisms and recommend a substantial revision of your manuscript before we can consider publication. If you are able to revise the manuscript along the lines suggested, which may involve further experiments, I will be happy receive a revised version of the manuscript. Your revised paper will be re-reviewed by one or more of the original referees, and acceptance of your manuscript will depend on your addressing satisfactorily the reviewers' major concerns. Please also note that Development will normally permit only one round of major revision.

We are aware that you may currently be unable to access the lab to undertake experimental revisions. If it would be helpful, we encourage you to contact us to discuss your revision in greater detail. Please send us a point-by-point response indicating where you are able to address concerns raised (either experimentally or by changes to the text) and where you will not be able to do so within the normal timeframe of a revision. We will then provide further guidance. Please also note that we are happy to extend revision timeframes as necessary.

Please attend to all of the reviewers' comments and ensure that you clearly highlight all changes made in the revised manuscript. Please avoid using 'Tracked changes' in Word files as these are lost in PDF conversion. I should be grateful if you would also provide a point-by-point response detailing how you have dealt with the points raised by the reviewers in the 'Response to Reviewers' box. If you do not agree with any of their criticisms or suggestions please explain clearly why this is so.

© 2021. Published by The Company of Biologists under the terms of the Creative Commons Attribution License (https://creativecommons.org/licenses/by/4.0/).

Reviewer 1

Advance summary and potential significance to field

Danescu et al present work investigating morphogenetic patterns responsible for morphogenesis of mesenchymal cells within the chicken frontonasal mass (FMN). Morphometrics are used to determine tissue-level shape changes in the FNM prior to outgrowth of the beak, which show mediolateral narrowing to be an important feature. Analysis of cartilage differentiation and orientation of proliferation are used to establish a developmental time window appropriate for testing how cell motility might be coordinated in vivo. The authors explore explant culture for studying FNM morphogenesis, finding that FNM morphogenesis is likely intrinsic to the tissue and is regulated, in part, by Rho kinase signaling. Tracking of nuclear motion within the FNM from live imaging experiments in WT and ROCK inhibitor treated explants is used to calculate how ordered cell motion is across the tissue. Interesting the authors find a spatiotemporal pattern of directional order during development. Further statistical methods are used to define additional spatiotemporal patterns of cell movement, calculate symmetry of directional order and correlation of that order with patterns of cell motion, all of which are disrupted by inhibition of ROCK.

Danescu et al cleverly identify the chicken frontonasal mass as a tractable model to study how movements of mesenchymal cells are coordinated across tissues to robustly generate shape change. This problem is outstanding because the cell-cell and cell-environment interactions that are known to regulate coordinated cell movements in well-studied epithelial structures, are very poorly understood in mesenchyme where cellular geometries are much more complex and live imaging is challenging. The tools implemented here uncover surprising mirror-symmetric spatiotemporal patterns of cell motion that explain characteristic and essential shape changes adopted by the FMN. These novel data suggest that mesenchyme can be coordinated over relatively long-distances by the activities of Rho kinases. This report is one of few quantitative studies of movements in a mesenchymal organ and could be a foundational study that, for the first time, offers the resolution to clarify hypotheses about midline/lip morphogenesis and facial symmetry.

Comments for the author

While the findings of this study are exciting and highly suitable for the readership of Development, there are quite significant problems that must be addressed and clarified before it would be appropriate for publication. However the number of comments here should also reflect an enthusiasm for the potential of the work.

1. Introduction paragraph 5 sets up the FNM as the midline which later is said to be ‘critical for lip fusion and general symmetry of the face’. However there is no reference to support this. What might these data suggest are the most important cellular behaviours in FNM morphogenesis?

2. It is not quite clear why data from Tao et al. 2019 is discussed in Introduction paragraph 5. Is it because physical properties of the tissue serve as a possible environmental regulator of cellular coordination? Or that physical properties shape the dynamics of morphogenesis, important for justifying experiments in fig. 2?

3. It is not immediately clear whether panels 1A-C are of the same scale. Could a scale bar be included? Alternatively this could be indicated in the text.

4. While the insets in Fig. 1A’-C’ showing sectional plane are helpful for experienced readers, it may be difficult for trainees to relate the frontal isosurface rendering to insets as orientation of their rostrocaudal axis are not equivalent. This could be remedied by rotating insets so that they represents a lateral view of the same orientation as A-C, section plane could indicated in at least one isosurface rendering or lateral view isosurface renderings could be added to the figure. In either case, the axis used to orient the reader throughout the text should be indicated for 1A-C.

5. The figure legend suggests that the arrows in 1A’-C’ indicate the narrowest and widest points within the FNM but it is not clear whether these arrows might also refer to those points measured in D and E. As such, It is important to clarify what is meant by superior and inferior to nasal slits relative to 1A’-C’.

© 2021. Published by The Company of Biologists under the terms of the Creative Commons Attribution License (https://creativecommons.org/licenses/by/4.0/).

6. The descriptions of Fig. 1D and E in the results text are insufficient.
While Fig1D may represent a complex change (getting wider, then narrower), 1E is more simple as it only decreases in width. Thus, the authors could be more specific about where shape change is more complex in the FNM which would help the reader conceptualise the system.

7. Fig.1 G fails to demonstrate that volume increase of the FNM is attributable to thickening in the dorsoventral axis, as reference image/measurement of an earlier stage has not been included. Further, no scale bar is included. These should be added to the figure.

8. It is not clear what the red line indicates in Fig.1G. Does it indicate D-V growth?

9. Section plane of Fig1G has not been indicated. This is important as the rostro-caudal orientation of this section is rotated counter-clockwise relative to the insets in 1A'-C', corresponding more to the orientation represented in 1A-C.
Furthermore, the mediolateral position of this slice is not indicated.

10. It is not clear whether Fig.1G is a slice view from the 3D acquisitions or an image of a sectioned embryo.

11. The use of coronal and frontal to denote section plane/orientation in Fig. H-M is not ideal. In many contexts, these are used synonymously and could confuse many readers. Horizontal could serve as an alternative.

12. Figure legend 1 indicates that most divergence in shape between st28 and 29 is in the cr-ca and M-L axes however the text indicates significant change in all an axes. This should be made consistent or removed from the legend.

13. PCA is used to assess shape change and the authors suggest that this change is best represented with PC1 but as these data are not shown, it is not clear, first, whether these data recapitulate the difference in shape changes in the first 24hrs compared to the second that are described later in the text. Including at least PC1 in supplemental data could be helpful.

14. While merited, the justification for assessing cartilage initiation in the FNM requires further clarification. Why might the dynamics before this event be important/more relevant to their question? Exploring this might better justify further experiments.

15. The authors suggest that although Sox9 expression is present in the FNM from st 24, Col2 is not present even at st 29. These data should be included if this point is to be made.

16. The justification for assaying cartilage differentiation is to avoid looking at stages where ECM for example might change the behaviour. However, the authors do not find significant Col2 throughout the stages previously analysed suggesting this stage could be used to address the authors questions. Alternately to my previous comment, Col2 localisation could be removed.

17. Col2 assembly in the cartilage is a relatively late addition to the milieu of ECM generated by differentiating cartilages, e.g. Fibronectin and Tenascin c which peak during early chondrogenic differentiation (briefly reviewed in Bobick et al 2009). This could link to the previous two points.

18. It is not entirely explained why it is also important to analyse growth before lip fusion. Also, a reference for the onset of lip fusion was not included.

19. The distribution of division angles in Fig2G appears to show fewer cells that divide 90 to the cr-ca axis compared to 2H but it is suggested that are oriented at 90 in both metaphase and telophase because the mean angle is the same.
Further statistics may be needed to confirm this claim. Alternatively, as telophase correction of division angle is well documented 2G could be omitted as 2H is the better indication of division angle.

© 2021. Published by The Company of Biologists under the terms of the Creative Commons Attribution License (https://creativecommons.org/licenses/by/4.0/).

20. While not essential to address, the question of whether division could drive shape change posed here could additionally be estimated by calculating what volume the observed proliferation rate might add.

21. The justification/transition for assessing the role of surrounding structures in FNM morphogenesis could be improved by explaining why they are important to rule out.

22. It is not immediately clear how the percentage decrease in FNM width in culture compares to the percentage decrease in whole embryos? This should be plotted alongside quantification of cultured FNMs e.g. in Fig3F. The deviation of this decrease should also be indicated where percentage decrease is referenced in the text. This is necessary to support the claim that morphogenesis retained in culture approximates that of the in vivo condition. Equivalent images of undissected faces could also be included but is not essential.

23. It is not immediately clear whether the no eyes condition plotted in Fig3F corresponds to no eyes no brain pictured in D-D' or a different condition.

24. Figure legend 3 suggests that ROCKi even increased FNM width but this is not strongly reflected in the graph. This could be toned down.

25. Fig legend 3 does not describe the measurements presented in Fig3F.

26. The data demonstrating that the maxillary and mandibular prominences are also not required for morphogenesis should be included as this is the condition used in following experiments.

27. The authors ask whether FNM 'cells themselves are providing shape information or whether it was mainly the extracellular matrix' suggesting that ROCK inhibition distinguishes these possibilities. While the cytoskeleton can be controlled cell autonomously, cellular interactions with the ECM can also modulate cytoskeletal regulators such as the GTPases inhibited by ROCKi. Further experiments in other contexts suggest that ROCKi may influence ECM synthesis.
Further experiments would be needed to make this argument. Alternatively, this claim could be toned down.

28. The figure callout for ROCKi treatment in Fig. 3 is mistakenly indicates Fig. 2.

29. While results presented in Fig. 3 suggest that FMN growth grows normally in culture, the conditions for cultures analysed in the remaining experiments differ in the physical forces exerted on the tissue (i.e. embedded in 100% Matrigel). Therefore, the percentage narrowing observed should also be shown, at least in supplemental data.

30. Further to the above point, does the shape defect in ROCKi treated cultures in Matrigel compare to the shape generated without? An image which shows more clearly the representative shape of the FMN in that culture system, or average width/Cr-Ca length measurement could be included to address this concern.

31. ROCKi may also influence cell adhesion patterns, which can influence motility and tissue shape/packing. While not essential, a metric of cell density across FMN in control or treated cultures could address whether the long range signal predicted to be important here, is not a particular morphogen but rather physical coupling of cells across the tissue.

32. Proliferation can also be promoted by ROCKi. As it is possible that FMN narrowing could be the result of an increased cell numbers, a metric of cell division fromexisting live imaging data or fixed ROCKi treated samples would be important to exclude this possibility.

33. Why the 'edges of clusters of cell vectors are potentially locations where stability could be perturbed by unknown environmental factors' is not fully explained.

© 2021. Published by The Company of Biologists under the terms of the Creative Commons Attribution License (https://creativecommons.org/licenses/by/4.0/).

34. It is not entirely clear why diffuse clusters of ROCKi treated cultures might indicate disturbed signalling as oppose to the ability of the cell to execute a response to the signal is disrupted (end of results referring to fig 5).

35. In the results referring presented in fig 6, convergence and divergence are hypothesised to serve as a proxy for identifying the presence of signalling source and sink. The reasoning behind this hypothesis is not well explained although Cai ad Montell 2014 are cited. This 2014 review, describes source and sink signalling systems used by migrating cell populations. While an interesting idea for this tissue, source and sink systems are typically utilised in cell collectives which migrate over much longer distances than seen in the FMN. As such, other mechanisms might be sufficient to promote directionality of cell movement. Further, how might cells shift the axis of a gradient by 90 between convergence and divergence? How does the time scale of gradient generation, for example in the lateral line, compare to the pause before switching direction in this context? How does the size of the clusters of cell movements compare to the size of cell collectives that use this system? The need for a source or sink is not expressly required by the data presented here and could be removed to avoid confusion. Alternatively, further discussion and potentially more experiments would be required, especially as it is not intuitive that ROCKi might interfere with processes involved in local gradient formation, such as secretion, signal reception and gene expression in this system.

36. An M-L band of divergence in the first phase of order calculated from 10x data is not immediately obvious in fig 6. Thus the claim that this band is replicated in both data sets is a little strong and hides the importance of the higher magnification. It could be helpful to emphasise how Fig7 adds and is different to fig 6 (e.g. the interpolation length).

37. It is not entirely clear how the 10-20 min interval for dynamic switching is derived and, therefore, not obvious why the signal would be rapidly oscillating when a divergent band can reside for up to an hour?

38. Further explanation is required in the first paragraph of the discussion to make clear why the data presented here implicate signalling systems in the FNM. (see also comment 35).

39. Does the average distance of medial motion of mesenchyme correspond to the extent of narrowing of the FMN in the first 4 hours of treatment? Not essential but would help clarify to what extent cell motion regulated morphogenesis here.

40. Why might GTPase activity in particular is likely to be responsible for buffering against environmental signals is not well justified in both results and discussion. Further explanation and possibly data would be important to make this point as a buffering effect would only be apparent in the context of an additional perturbation. Alternatively this point could be toned down/clarified further.

41. Many of the concepts used in the results and discussion, such as the buffering suggested here, and length cell of cell coordination across the tissue should be explored in the introduction to make it easier for the reader to link the inferences made later in the manuscript.

42. Fig. 9 is not referred to in the text, therefore, it is not clear whether this figure is needed. Although WNT5A expression is discussed, the other molecules and their potential to mediate the cell behaviours observed in this report are not discussed. Further, the black arrows indicating morphogenetic motion do not highlight the

<u>Reviewer 2</u>

Advance summary and potential significance to field

In this manuscript Danescu et al study the movement of facial mesenchymal cells. They describe a new technique of explant culture that allow for time-lapse imaging of these cells. First, they describe the morphology of the region of interest using 3D reconstruction of chick embryo head, then show that this experimental system allows for reproduction of the narrowing of the tissue

© 2021. Published by The Company of Biologists under the terms of the Creative Commons Attribution License (https://creativecommons.org/licenses/by/4.0/).

linked to normal morphogenesis. By manual tracking and using computational methods they show that motions of cells alternate between ordered and disordered phases therefore displaying a high degree of coordination of cell movements over large distances. Then, they show that these global movements are symmetric between the left and right sides and that they are defined by a succession of divergence and convergence events. Finally, they show that these divergence and convergence events are oscillating. They show that coordination, symmetry and periodicity of cellular movements are affected by RhoGTPase inhibitor drug treatment suggesting that they involve cytoskeletal reorganization. The manuscript is particularly interesting because it describes a new culture / time-lapse: image analysis techniques that are suitable to model and therefore study late relatively morphogenesis and pathologies such as cleft palate. The use of new computational tools allowing for probing the coordination of cell movements over large distances is very powerful and potentially useful for a broad scientific community. Experiments are well executed, deeply analyzed and conclusions sound appropriate. Overall, long distance coordination of cellular movements is understudied although this process is very likely to have critical roles in morphogenesis. Therefore, I believe that this type of work can be a very good fit for Development. However, in its current form the work has some major flaws (particularly concerning the biological relevance of the experimental system) that would need to be addressed by the authors. Particularly it is not clear if the cellular dynamics observed are due to the experimental set up or are a reflect of a genuine biological process taking place in the embryo. I do think that if most of my concerns are addressed by appropriate experiments and analysis this manuscript should be accepted for publication in Development.

Comments for the author

Major comments :

- It is still possible that the global movements observed are a consequence of global contractions of the explants related to the culture conditions. Studying cellular neighbor's relations over time is therefore of interest to show that there are cellular rearrangements (such as intercalation) linked to the morphogenetic process taking place in the culture.

- Along the same lines, the authors present a new experimental culture conditions, I believe it is necessary to show sections (to document the thickness/shape of the explant), proliferation and cell death data for both control and experimental conditions.

- Rock inhibitor has quite a general and drastic effect on developing tissues; it is therefore not surprising that it affects all the properties that has been observed in control tissues. The analysis of the effect of treatments inhibiting signaling pathways implicated in the morphogenesis of the region (such as Shh or Wnt would be very convincing to show that 1) the experimental system is suitable for further mechanistic studies, 2) that the global patterns observed are part of the morphogenetic program at work in the tissue.

Minor comments :

- I was confused about the convergence / divergence data 30 x (Fig7). I don't understand why we already see the major band of divergence and we don't see convergence at 100 min as it is documented on the 10 x (Fig. 6)
- I have not find in which solvent has been dissolved the ROCKi. Have the control experiments been made with the same percentage of solvent?

<u>Reviewer 3</u>

Advance summary and potential significance to field

The authors present an analytical approach to defining mesenchymal cell movements in the frontonasal mass of the chicken embryo. They identify unexpected cell movements that are symmetrical on the left and right sides and oscillate between divergent and convergent patterns. These observations have not previously been made and the analysis is original in the way it combines spatial clustering of cells with patterns of movement on either side of the midline. Perturbation with ROCKi disrupts symmetry and the midline as a structural barrier that distinguishes right and left morphogenetic character. The implication is that, although left-right

© 2021. Published by The Company of Biologists under the terms of the Creative Commons Attribution License (https://creativecommons.org/licenses/by/4.0/).

facial symmetry is robust under wild-type conditions, genetic or other perturbations can readily disrupt that symmetry to cause facial dysmorphism. The unique approach to analysis of cell movements in this manuscript is valuable as are the observations concerning spatially-defined order between cells and unexpected phases of convergence and divergence across the midline. There are some assumptions made in the manuscript about the nature of cell movements and conceptual challenges in following the logic of the second half of the results and discussion that require clarification.

Comments for the author

It's not clear in the introduction why left-right symmetry necessarily implies genetic control. Could biophysical constraints (beyond the brain and eye) account for those observations?

Postnatal facial asymmetry is greater among UCLP patients. Is this morphogenetic effect necessarily related to cell movements?

In Fig. 1, consider whether the addition of sagittal OPT views would optimally illustrate convergent extension. Also consider whether the coronal plane would be better labelled as axial, and frontal as coronal.

Would it be appropriate to note that changes in the SOX9 domain shown in Fig. 2A-C support convergent extension?

I didn't understand why mediolaterally separating daughter cells would not be visualised equally from the frontal and the (top down, or axial?) coronal plane. I do expect they wouldn't be seen from the sagittal plane.

The arrowheads in Fig. 2E, D are not pointing at PH3 +ve cells and are not very necessary.

The polar plots in Fig. 2G, H are fine, but I wonder if they would be more immediately clear if they were shown as a full circle with symmetrical wedges about the centre so that the reader does not mistakenly wonder whether daughter cells preferentially emerge toward one side.

Since the orientation of cell division does not correspond to convergent extension, could daughter cell intercalation be restore the widening caused by their separation?

In the Dynamic cell movements section of the results, it would be helpful to add a sentence describing the imaging method.

Please briefly define radius and the purpose of averaging cosine where those concepts are introduced in the results.

Please provide a legend for the solid and dashed lines in Fig. 4 H', I'.

It may be useful to add that ROCKi disrupts individual cell behaviours in addition or as an alternative to disruption of cell-cell and cell-matrix communication.

Please define k-mean clustering where it is introduced in the results.

It would be helpful to explain a little more how p and q weighting was derived in the results.

Does "50 µm area" refer to radius?
In addition to signalling, preprogramming and genetic control, long range coordination of cell movements and symmetry might also be achieved by physical constraints.
Please elaborate on the reason that order corresponds to switches between divergence and convergenc Although it makes sense that Rho GTPases serve as hubs for signalling cascades that can be disrupted by ROCKi, it wasn't very clear how such a hub might mediate physiological reversal of cell movements. In citing Gros et al, 2010, it may be more appropriate to note they showed FGF promotes, rather than orients mesenchymal cell movements.

© 2021. Published by The Company of Biologists under the terms of the Creative Commons Attribution License (https://creativecommons.org/licenses/by/4.0/).

It wasn't clear how instructive source and sink cues might correspond to oscillation between convergence and divergence of cell movements. In the case of Wnt5a as a potential source leading to cell convergence why would the duration be brief and intermittent?

First revision

Author response to reviewers' comments

Dear Editor,

This revision has now addressed all the concerns of the reviewers. New data and analysis has been added to strengthen the conclusions.

I will first summarize all of the changes:
1. Text of the main manuscript has been extensively rewritten.
2. Figures in the main paper have been edited including Figure 1, 2,3 (new graph in 3G), 7A- E.
3. Eight new supplemental figures have been included: Figs. S1-S7 and S15.

Point by point rebuttal for DEVELOP/2020/193755

Reviewer 1

Comments for the Author... While the findings of this study are exciting and highly suitable for the readership of Development, there are quite significant problems that must be addressed and clarified before it would be appropriate for publication. However, the number of comments here should also reflect an enthusiasm for the potential of the work.

1. Introduction paragraph 5 sets up the FNM as the midline which later is said to be 'critical for lip fusion and general symmetry of the face'. However, there is no reference to support this. What might these data suggest are the most important cellular behaviours in FNM morphogenesis?
We have rewritten the introduction to take the reader through the concept of developmental instability and evidence that orofacial clefting is an example of how a further destabilization of a naturally occurring set of fluctuations could lead to a cleft lip. The most common type of orofacial cleft is unilateral and there are no other organs affected, thus non-syndromic. The role of genetics is relatively minor compared to environmental factors that increase the risk of clefting. I have cited papers by Richmond, Weinberg, Starbuck and others that measure developmental instability as fluctuations in symmetry across individuals related to those born with clefts. We stress that there is evidence of developmental instability in the face shown in mouse studies in which genetic background is carefully controlled. Even when there are no genetic differences between littermates, unilateral clefts occur in many gene targeting experiments as well as from teratogen exposures. However no one understands precisely the embryological basis of the developmental instability. In the results section we have rephrased the rational for examining symmetry. "The degree of symmetry is thought to be a readout of developmental instability. For this reason we measured fluctuating asymmetry of individual cell vectors in our samples. "
2. It is not quite clear why data from Tao et al. 2019 is discussed in Introduction paragraph 5. Is it because physical properties of the tissue serve as a possible environmental regulator of cellular coordination? Or that physical properties shape the dynamics of morphogenesis, important for justifying experiments in fig. 2?
We have moved the discussion of this paper to the end of the MS where we mention other possible mechanisms that could explain our results. This paper is mentioned only briefly in the introduction as the only study that has carried out live imaging on the face. In the discussion we explain that in their system the morphology is already present at the time they did their imaging so they assumed biophysical differences existed. In our system biophysical changes will occur in the midline but we focused our analysis before cartilage develops to keep the properties more uniform.
3. It is not immediately clear whether panels 1A-C are of the same scale. Could a scale bar be included? Alternatively this could be indicated in the text.

© 2021. Published by The Company of Biologists under the terms of the Creative Commons Attribution License (https://creativecommons.org/licenses/by/4.0/).

We added a scale bar for the isosurfaces based on new measurements of the reconstructions in Amira (Fig. S1). All scale bars from A-C' = 500 microns.

4. While the insets in Fig. 1A'-C' showing sectional plane are helpful for experienced readers, it may be difficult for trainees to relate the frontal isosurface rendering to insets as orientation of their rostrocaudal axis are not equivalent. This could be remedied by rotating insets so that they represents a lateral view of the same orientation as A-C, section plane could indicated in at least one isosurface rendering or lateral view isosurface renderings could be added to the figure. In either case, the axis used to orient the reader throughout the text should be indicated for 1A-C.

Lateral isosurfaces have replaced the previous insets to show planes of section

5. The figure legend suggests that the arrows in 1A'-C' indicate the narrowest and widest points within the FNM but it is not clear whether these arrows might also refer to those points measured in D and E. As such, It is important to clarify what is meant by superior and inferior to nasal slits relative to 1A'-C'.

We have edited the figure to label the nasal slit, added an axis key, changed terminology in D, E to cranial and caudal to be consistent with the wireframes

6. The descriptions of Fig. 1D and E in the results text are insufficient. While Fig1D may represent a complex change (getting wider, then narrower), 1E is more simple as it only decreases in width. Thus, the authors could be more specific about where shape change is more complex in the FNM which would help the reader conceptualise the system.

We have edited the results to say that different parts of the FNM as seen in 2D undergo expansion and others contract, therefore morphogenesis is complex. The likelihood that cell movements, if they are taking place will be different according to their anatomical location.

7. Fig.1 G fails to demonstrate that volume increase of the FNM is attributable to thickening in the dorsoventral axis, as reference image/measurement of an earlier stage has not been included. Further, no scale bar is included. These should be added to the figure.

The relative thickening with increased development is shown in the wireframes in 1I versus 1L. I have also added a scale bar in 1G as well as an inset showing the plane of section.

8. It is not clear what the red line indicates in Fig.1G. Does it indicate D- V growth?

9. Section plane of Fig1G has not been indicated. This is important as the rostro-caudal orientation of this section is rotated counter-clockwise relative to the insets in 1A'-C', corresponding more to the orientation represented in 1A-C. Furthermore, the mediolateral position of this slice is not indicated. It is not clear whether Fig.1G is a slice view from the 3D acquisitions or an image of a sectioned embryo

Yes the red line does show the DV thickness. This is now clearer because we have put in the plane of section as an inset. Yes this is a slice view and this has been added to the legend.

10. The use of coronal and frontal to denote section plane/orientation in Fig. H-M is not ideal. In many contexts, these are used synonymously and could confuse many readers. Horizontal could serve as an alternative.

Now that we have put in clearer insets of the plane of section of the wireframe in Fig 1 H-M, the reader will understand what is meant by coronal. We used coronal because it is a plane, parallel to the top of the head. The body axis of embryos is not straight hence the terminology is sometimes confusing. Note that the landmarks can be identified in the wireframes and in Fig. 1B which also provides a reference for the axis being shown.

11. Figure legend 1 indicates that most divergence in shape between st28 and 29 is in the cr-ca and M-L axes however the text indicates significant change in all an axes. This should be made consistent or removed from the legend.

Thanks for pointing this out. We have shown that there is significant medial displacement of the landmarks that can be seen in K and M, expansion in the cranial caudal axis shown in L and M. The legend has been rewritten

12. PCA is used to assess shape change and the authors suggest that this change is best represented with PC1 but as these data are not shown, it is not clear, first, whether these data recapitulate the difference in shape changes in the first 24hrs compared to the second that are described later in the text. Including at least PC1 in supplemental data could be helpful.

*We have added this geometric morphometrics analysis into a new supplementary figure S1. The original segmentation *.ply file is included, the PCA plot after Procrustes superimposition showing separation of the three stages, the Eigenvalues to show that PC1 explains over 80% of the variation and a graph showing centroid sizes are significantly larger in the stage 29 specimens*.

13. While merited, the justification for assessing cartilage initiation in the FNM requires further clarification. Why might the dynamics before this event be important/more relevant to their question? Exploring this might better justify further experiments.

© 2021. Published by The Company of Biologists under the terms of the Creative Commons Attribution License (https://creativecommons.org/licenses/by/4.0/).

We have added text in the results to say that the biophysical properties will change during cartilage condensation formation since ECM will be stiffer. Prior to this cells are tightly packed with little ECM between them (see new images in Fig. S7C,D with Myosin II staining for cytoskeleton plus cell density calculations, Fig. S6B).

14. The authors suggest that although Sox9 expression is present in the FNM from st 24, Col2 is not present even at st 29. These data should be included if this point is to be made.

The new Fig. S3 shows staining of embryos at stage 26 and 29 with Col2a1 antibody. While there is expression of Col2a1 in Meckel's cartilage at stage 29, the frontonasal mass has no expression. We have noted this relative delay of the frontonasal mass cartilage differentiation in our 2013 paper using chondroitin sulfate antibody staining (Hosseini-Farahabadi et al., 2013).

15. The justification for assaying cartilage differentiation is to avoid looking at stages where ECM for example might change the behaviour. However, the authors do not find significant Col2 throughout the stages previously analysed suggesting this stage could be used to address the authors questions. Alternately to my previous comment, Col2 localisation could be removed.

Although it is true that Col2a1 expression is starting around stage 29-30, using stage 28 embryos was not possible due to increased tissue thickness and poor translucency. We have justified our experimental design more fully on page 8.

16. Col2 assembly in the cartilage is a relatively late addition to the milieu of ECM generated by differentiating cartilages, e.g. Fibronectin and Tenascin C which peak during early chondrogenic differentiation (briefly reviewed in Bobick et al 2009). This could link to the previous two points.

Yes there are other ECM molecules that are secreted prior to chondrogenesis. The paper by Gluhak et al., 1996 from Mina Mina's lab examined Tenascin C RNA in the chicken mandible (no other parts of the head were studied). The RNA was visible at stage 26. The protein however was not visible until stage 30. Therefore since the mandible is ahead of the frontonasal mass it is very unlikely that Tenascin C is expressed in the stage 25 frontonasal mass organ cultures. It is most likely that type I collagen and fibronectin make up the bulk of the ECM at the stages we have chosen.

17. It is not entirely explained why it is also important to analyse growth before lip fusion. Also, a reference for the onset of lip fusion was not included.

Actually is it not the fusion event that we are studying. Our best paper on the exact timing of lip fusion is this one by Abramyan et al. 2015 (doi: 10.1111/joa.12291). We have removed the sentence saying that we are trying to target stages prior to lip fusion since this is tangential. Instead we have emphasized the delicate balance of growth, contact and adhesion between the frontonasal mass and the maxillary prominence needed for lip fusion to take place.

18. The distribution of division angles in Fig2G appears to show fewer cells that divide 90 to the cr-ca axis compared to 2H but it is suggested that are oriented at 90 in both metaphase and telophase because the mean angle is the same. Further statistics may be needed to confirm this claim. Alternatively, as telophase correction of division angle is well documented 2G could be omitted as 2H is the better indication of division angle.

At your suggestion we removed the metaphase data. Here are the raw data for telophase:

Parameter	Value
**Title:**	**Stage 24 Telophase**
**Set:**	**24-1**
Number of measurements:	61
Largest class size:	25%
Mean:	90.76°
Standard deviation:	±34.94°
Vector magnitude:	49.66
Consistency ratio:	0.81
**Set:**	**24-2**
Number of measurments:	60
Largest class size:	17%
Mean:	99.30°
Standard deviation:	±38.55°
Vector magnitude:	46.42
Consistency ratio:	0.77

© 2021. Published by The Company of Biologists under the terms of the Creative Commons Attribution License (https://creativecommons.org/licenses/by/4.0/).

**Set:**	**24-3**
Number of measurments:	64
Largest class size:	19%
Mean:	83.83°
Standard deviation:	±45.02°
Vector magnitude:	44.24
Consistency ratio:	0.69
**Average Telophase**	**91.3**
**SD**	**7.8**
**Total cell number**	**185**

19. While not essential to address, the question of whether division could drive shape change posed here could additionally be estimated by calculating what volume the observed proliferation rate might add.

We did not add new data on this because we would need to have an estimate of the volume of a cell. The methods used here do not allow us to make this calculation. However we have shown pH3 labeling in vivo in Figure 2D-F and in vitro in Figure S6A-B'. There is more proliferation near the nasal slits particularly in vivo which could conceivably lead to thickening in those regions. In vitro the proliferation seems a bit more even although it is hard to quantify this. I prefer not to comment on the role of cell division at these early stages. Clearly there is a role in older embryos when the upper beak has a distinct growth zone at the tip that is characteristic of the bird species (see work by Wu et al., 2006 and from the Schneider lab)

20. The justification/transition for assessing the role of surrounding structures in FNM morphogenesis could be improved by explaining why they are important to rule out.

We have added a full section to transition the reader from in vitro organ cultures to the imaging cultures. This is on most of page 8 in the results section and refers to new data Figs. S5 and S6. We bring the reader through the reasons for cutting down the cultures further and provide detailed results on the health of the cultures in the presence and absence of the ROCKi.

21. It is not immediately clear how the percentage decrease in FNM width in culture compares to the percentage decrease in whole embryos? This should be plotted alongside quantification of cultured FNMs e.g. in Fig3F. The deviation of this decrease should also be indicated where percentage decrease is referenced in the text. This is necessary to support the claim that morphogenesis retained in culture approximates that of the in vivo condition. Equivalent images of undissected faces could also be included but is not essential.

The whole head is growing in size at the same time as the midface is becoming narrower. We have added in new 3D linear measurements directly from the OPT scans imported into Amira 3D visualization software. The graph and statistical analysis of this data is Figure S1. The head increases in width by about 38% from stage 24-29. Yet the frontonasal mass narrows The eyes increase in volume dramatically as does the brain. However interestingly the centre of the face narrows. We can see this dramatically in the embryos that we work on daily. In Figure 3G we have added the absolute measurements of width in the facial cultures and they do correspond very closely to the in vivo measurements in Figure S1 (mentioned at the top of page 8). Therefore the cultures imaged at 24 and 48h are equivalent to are developing almost exactly as in vivo. The cultured face is initially equivalent to stage 24 in vivo dimensions. Then after 24h of culture, the frontonasal mass narrows to the same dimensions as a typical stage 28 embryo. After 48h of in vitro culture, the frontonasal mass narrows the equivalent width of a typical stage 29 embryo. We did the 3D measurements in Amira blind to the other results obtained earlier in organ culture.

22. It is not immediately clear whether the no eyes condition plotted in Fig3F corresponds to no eyes no brain pictured in D-D' or a different condition.

Sorry for this error. 3F has been corrected to say no eyes or brain.

23. Figure legend 3 suggests that ROCKi even increased FNM width but this is not strongly reflected in the graph. This could be toned down.

We have corrected the text describing Figure 3F accordingly "We found that the ROCK inhibitor completely blocked narrowing".

24. Fig legend 3 does not describe the measurements presented in Fig3F.

The legend has been corrected.

© 2021. Published by The Company of Biologists under the terms of the Creative Commons Attribution License (https://creativecommons.org/licenses/by/4.0/).

25. The data demonstrating that the maxillary and mandibular prominences are also not required for morphogenesis should be included as this is the condition used in following experiments.
New data showing morphogenesis occurring without the maxillary and mandibular prominences over 24h of culture is in Figure S4.
26. The authors ask whether FNM 'cells themselves are providing shape information or whether it was mainly the extracellular matrix' suggesting that ROCK inhibition distinguishes these possibilities. While the cytoskeleton can be controlled cell autonomously, cellular interactions with the ECM can also modulate cytoskeletal regulators such as the GTPases inhibited by ROCKi. Further, experiments in other contexts suggest that ROCKi may influence ECM synthesis. Further experiments would be needed to make this argument. Alternatively, this claim could be toned down.
We agree that we did not measure the integrin mediated interactions between the cytoskeleton and the ECM. We removed this sentence in the results and instead have written: "These ROCKi results suggested that the functioning cytoskeleton was essential for normal frontonasal morphogenesis."
27. The figure callout for ROCKi treatment in Fig. 3 is mistakenly indicates Fig. 2.
corrected
28. While results presented in Fig. 3 suggest that FMN growth grows normally in culture, the conditions for cultures analysed in the remaining experiments differ in the physical forces exerted on the tissue (i.e. embedded in 100% Matrigel). Therefore, the percentage narrowing observed should also be shown, at least in supplemental data.
Additional experiments were conducted for this comment. Unfortunately we were not able to see the nasal slits clearly enough to measure the narrowing between time 0 and 4.5h so we could not calculate the percentage of narrowing within each specimen. However we did confirm that there is a phenotype with the ROCKi compared to controls in width of the frontonasal mass grown in Matrigel under the exact conditions used for imaging (new Fig. S6,S7).
29. Further to the above point, does the shape defect in ROCKi treated cultures in Matrigel compare to the shape generated without? An image which shows more clearly the representative shape of the FMN in that culture system, or average width/Cr-Ca length measurement could be included to address this concern.
We did not do geometric morphometrics in the organ cultures, in part because there are distortions that come from the dissection borders and outgrowth of underlying mesenchyme as see in the images in Fig. S5A'-G'. We always see flattening and spreading of the cultures in the presence of the ROCKi as in Figure 3E-E''. All the facial prominences are affected as shown by loss of distinct borders in Fig. 3E''. For consistency with the live imaging data, we used the sections of the frontonasal mass at an equivalent plane to measure the width and yes there is an effect produced by ROCKi during the imaging period of 4.5h (Fig. S7 right column). However now after reanalyzing the data, the widening of the ROCKi cultures occurred almost immediately and did not further widen during subsequent culture times (Fig. 3G).
30. ROCKi may also influence cell adhesion patterns, which can influence motility and tissue shape/packing. While not essential, a metric of cell density across FMN in control or treated cultures could address whether the long range signal predicted to be important here, is not a particular morphogen but rather physical coupling of cells across the tissue.
The reviewer raised an interesting point. ROCKi could have decreased cell density, especially since the dimensions of the tissue are increased. However we measured density in the sections and did not find a significant difference (Fig. S6). In addition we showed staining with non-muscle myosin II which stains the majority of the cytoskeleton. This new data in Fig. S6, S7, shows that there is close packing of cells. We attempted to measure the space in between the cells and the cell size to see whether the cytoskeleton was more contracted in the presence of ROCKi but this was difficult to do in a reproducible manner. Nevertheless it is likely there is no difference since the number of nuclei in a 100 micron squared area is the same in controls and treated embryos (Fig. S6B, S7A', B'). The reason there is no decrease in cell density even though the FNM is wider is that cells are compressed into a thinner organ in the Z axis. Note there is no change in proliferation from ROCKi either (see next point).
31. Proliferation can also be promoted by ROCKi. As it is possible that FMN narrowing could be the result of an increased cell numbers, a metric of cell division from existing live imaging data or fixed ROCKi treated samples would be important to exclude this possibility.
We did pH3 antibody staining and counted proliferating cells across the entire frontonasal mass in the cultures (Fig. S6, S7). There was no significant difference between treated and control cultures. Therefore addition of new cells does not seem to be the reason for why the ROCKi cultures are

© 2021. Published by The Company of Biologists under the terms of the Creative Commons Attribution License (https://creativecommons.org/licenses/by/4.0/).

widening. The flattening may be due to relaxing of the cell cytoskeleton.

32. Why the 'edges of clusters of cell vectors are potentially locations where stability could be perturbed by unknown environmental factors' is not fully explained.

We rewrote this sentence on page 10 of the results as follows: "The edges of the clusters of cell vectors (areas where average direction of the cells differs from neighbouring cells) may not form as consistently in the presence of environmental stresses. For example any stress that affects the cytoskeleton could prevent regional clustering as we observed with the ROCKi (Fig. 5B-B'', Fig. S9D-F'')".

33. It is not entirely clear why diffuse clusters of ROCKi treated cultures might indicate disturbed signalling as oppose to the ability of the cell to execute a response to the signal is disrupted (end of results referring to fig 5).

You are right the most direct reason that having a static cytoskeleton would naturally affect the ability of cells to respond to signals. The text has been rewritten on page 10.

34. In the results referring presented in fig 6, convergence and divergence are hypothesised to serve as a proxy for identifying the presence of signalling source and sink. The reasoning behind this hypothesis is not well explained although Cai ad Montell 2014 are cited. This 2014 review, describes source and sink signalling systems used by migrating cell populations. While an interesting idea for this tissue, source and sink systems are typically utilised in cell collectives which migrate over much longer distances than seen in the FMN. As such, other mechanisms might be sufficient to promote directionality of cell movement. Further, how might cells shift the axis of a gradient by 90 between convergence and divergence? How does the time scale of gradient generation, for example in the lateral line, compare to the pause before switching direction in this context? How does the size of the clusters of cell movements compare to the size of cell collectives that use this system? The need for a source or sink is not expressly required by the data presented here and could be removed to avoid confusion. Alternatively, further discussion and potentially more experiments would be required, especially as it is not intuitive that ROCKi might interfere with processes involved in local gradient formation, such as secretion, signal reception and gene expression in this system.

First, the movements we observed are not collective migration. The cells are not migrating large distances to transport themselves hundreds of microns away but are instead moving locally. From our track analysis we saw that at each 10 minute interval that there was a main direction taken but that the direction could change in the next 10 minute interval. In order to represent these movements more clearly to the reader we are providing the 30X tracking. A T-test compared overall speed of cells and found the ROCKi significantly slowed down movements (Fig. S15A). We had suspected that the regions close to the centre of the frontonasal mass moved less than those at the cranial and caudal ends. We therefore broke down the imaged area into subregions. Using mean square displacement, (Fig. S15B) the cumulative distance covered is greater in regions 4 and 6 (cranial and caudal) compared to the centre, region 5. Also the ROCKi treatment decreased the distance covered. To measure the directness of the migration we used autocorrelation (Fig. S15C). Here there is no difference in the persistence of cells going in one direction between the treated and control data. However, the cranial and caudal regions moved more directly than the centre in which direction was very poorly correlated with time. A qualitative analysis where all tracks were represented and place on a single point of origin reveals very clearly the cranial-caudal polarization of cell movements (Fig. S15D). The ROCKi appeared to make the tracks diverge in a less organized manner (Fig. S15,E).

35. An M-L band of divergence in the first phase of order calculated from 10x data is not immediately obvious in fig 6. Thus the claim that this band is replicated in both data sets is a little strong and hides the importance of the higher magnification. It could be helpful to emphasise how Fig7 adds and is different to fig 6 (e.g. the interpolation length).

The 30X data did not have clear peaks and troughs (Fig. S14A-I) so we originally chose 100 and 200 minutes as representative timepoints. However, looking closely at the data for the 5 control and 3 ROCKi treated specimens (Fig. 7A-H) we found that earlier in the culture there was more convergence present. To better show this we have updated Fig. 7A-H with earlier timepoints. The ROCKi treated cultures imaged at 30X displayed branched, divergent vectors mimicking the patterns at the lateral edges of the cultures imaged at 10X (movie 12).

36. It is not entirely clear how the 10-20 min interval for dynamic switching is derived and, therefore, not obvious why the signal would be rapidly oscillating when a divergent band can reside for up to an hour?

There are actually three measurements that fluctuated in our data. The first was order-disorder, in which the analysis was carried out on the raw data. The second was symmetry which was carried out on normalized data and here we saw a degrading of symmetry that correlated with disorder.

© 2021. Published by The Company of Biologists under the terms of the Creative Commons Attribution License (https://creativecommons.org/licenses/by/4.0/).

Last we examined convergence and divergence on the normalized dataset and found fluctuation over time. These results are now being discussed in a single section of the discussion to better pull the data together. What all the data show is that there are fluctuations between timepoints. Figure 8 is designed to help the reader to see the correspondence between the data from the raw vectors (order-disorder) and the normalized data (convergence-divergence). It was only after overlaying the two datasets that we and hopefully the reader can see there are major and minor fluctuations. The order-disorder has a longer periodicity between major peaks and troughs. The fluctuation of convergence and divergence is more rapid but the biggest switches in divergence occurred at the time when cultures transitioned between state of order and disorder. There may be different types of signals that regulate order and disorder, those that can change rapidly within 10-20 minutes and do not require new protein synthesis. We have data for the GTPases here so this is why we can suggest that this is one of the signals. Clearly ROCKi completely disrupts order-disorder and all other fluctuations as shown in Figs. S8,S9,S10,S12, S13.

37. Further explanation is required in the first paragraph of the discussion to make clear why the data presented here implicate signalling systems in the FNM. (see also comment 35).
The discussion has been rewritten to emphasize that we are not expecting our data to only apply to the frontonasal mass. This is just the starting point for future work on other parts of the face and other regions of the embryo. The fluctuation of cellular movements is likely to be a widespread phenomenon
38. Does the average distance of medial motion of mesenchyme correspond to the extent of narrowing of the FMN in the first 4 hours of treatment? Not essential but would help clarify to what extent cell motion regulated morphogenesis here.
This is a great suggestion. In our 10X data the resolution is too low to map distance travelled over time. From our 30X data we did calculate instantaneous speed (0.6 microns/h). You can see that the ROCKi significantly decreased speed. However cells do not go in a straight line so we can't calculate whether the cell movements contribute to narrowing for the controls. We included this data in Fig. S15A.

39. Why might GTPase activity in particular is likely to be responsible for buffering against environmental signals is not well justified in both results and discussion. Further explanation and possibly data would be important to make this point as a buffering effect would only be apparent in the context of an additional perturbation. Alternatively this point could be toned down/clarified further.
I have edited the discussion to explain that in the case of developmental instability, a rapid signal such as GEP/GDP is one possible moderator that could be easily susceptible to external stresses. Anything that changes upstream signals like WNTs for example would affect the balance between active and inactive small GTPases. This could happen locally and make a region on the left or right side of the face behave differently in terms of cell migration and cell signaling. Then an asymmetry would arise leading to unilateral cleft lip.
40. Many of the concepts used in the results and discussion, such as the buffering suggested here, and length cell of cell coordination across the tissue should be explored in the introduction to make it easier for the reader to link the inferences made later in the manuscript.
As mentioned in response to point 1 I have completely rewritten the introduction so the ideas of developmental instability and fluctuating asymmetry as a measure of developmental instability are introduced. I also explicitly say that most of the time there is tolerance for some developmental instability since in the majority of cases facial development occurs normally. I then go on to explain that all embryos have some developmental instability and that this is inherited. The evidence for this is coming from recent studies on unaffected relatives of individuals born with clefts. The prediction of developmental instability is that everyone has this and that relatives show have more fluctuating asymmetry than control populations. Indeed, this is the case. Therefore, we should be able to measure developmental instability in any embryo by measuring fluctuating asymmetry.
41. Fig. 9 is not referred to in the text, therefore, it is not clear whether this figure is needed. Although WNT5A expression is discussed, the other molecules and their potential to mediate the cell behaviours observed in this report are not discussed. Further, the black arrows indicating morphogenetic motion do not highlight the
Fig. 9 has now been explained in the first paragraph of the discussion.

Reviewer 2 Advance Summary and Potential Significance to Field...
However, in its current form the work has some major flaws (particularly concerning the biological

© 2021. Published by The Company of Biologists under the terms of the Creative Commons Attribution License (https://creativecommons.org/licenses/by/4.0/).

relevance of the experimental system) that would need to be addressed by the authors. Particularly it is not clear if the cellular dynamics observed are due to the experimental set up or are a reflect of a genuine biological process taking place in the embryo. I do think that if most of my concerns are addressed by appropriate experiments and analysis this manuscript should be accepted for publication in Development.

Reviewer 2 Comments for the Author...
Major comments : - It is still possible that the global movements observed are a consequence of global contractions of the explants related to the culture conditions. Studying cellular neighbor's relations over time is therefore of interest to show that there are cellular rearrangements (such as intercalation) linked to the morphogenetic process taking place in the culture. - Along the same lines, the authors present a new experimental culture conditions, I believe it is necessary to show sections (to document the thickness/shape of the explant), proliferation and cell death data for both control and experimental conditions. -
We have added new data in Figure S6 and S7 to characterize proliferation, cell death, cell density and width of the frontonasal mass in the imaged cultures. We also added a scale bar to a stage 28 embryo sagittal slice in Fig. 1G. The entire thickness of the frontonasal mass is no more than 150 microns at stage 24 (estimated from our sections that are each 7 microns thick).
Rock inhibitor has quite a general and drastic effect on developing tissues; it is therefore not surprising that it affects all the properties that has been observed in control tissues. The analysis of the effect of treatments inhibiting signaling pathways implicated in the morphogenesis of the region (such as Shh or Wnt would be very convincing to show that 1) the experimental system is suitable for further mechanistic studies, 2) that the global patterns observed are part of the morphogenetic program at work in the tissue.
I absolutely agree but this is beyond the scope of this study. This is our next paper
Minor comments : - I was confused about the convergence / divergence data 30 x (Fig7). I don't understand why we already see the major band of divergence and we don't see convergence at 100 min as it is documented on the 10 x (Fig. 6)
Same answer as for point 35 in Review 1's review. We arbitrarily chose two timepoints for the 30X data because there were no major peaks and troughs of order and disorder (Fig. S14). However earlier timepoints do show some convergence (New earlier timepoints in Fig. 7A-H). We are imaging the lateral edges of the culture so we would not expect to see the concentrated central zone of convergence that can be seen when the whole organ is imaged and tracked.
I have not find in which solvent has been dissolved the ROCKi. Have the control experiments been made with the same percentage of solvent?
Concentrated stock was diluted 1:1000. Therefore we added about 1 ul of DMSO to the cultures. This is added to the methods.

Reviewer 3 Advance Summary and Potential Significance to Field...
There are some assumptions made in the manuscript about the nature of cell movements and conceptual challenges in following the logic of the second half of the results and discussion that require clarification.

Reviewer 3 Comments for the Author...

It's not clear in the introduction why left-right symmetry necessarily implies genetic control. Could biophysical constraints (beyond the brain and eye) account for those observations?
Stress again independent NC streams that make up FNM mesenchyme
Postnatal facial asymmetry is greater among UCLP patients. Is this morphogenetic effect necessarily related to cell movements?
I have devoted more of the introduction to the concept of developmental instability which is inherent in embryos. I explain that measuring developmental instability is done in one of two ways, either by comparing right to left sides of the face or skull bones to look for fluctuating symmetry. Or you can measure shapes of objects and measure the standard deviation of those measurements. Those with increased variability are thought to have more developmental instability. Actually there is a huge literature on this topic and even an journal entitled Symmetry. I have cited a few of these papers by Thornhill and van Dongen, who have studied fluctuating asymmetry in a variety of organisms. This introduction prepares the reader for the idea there is developmental instability in the face as detected by measuring fluctuating asymmetry. Also that increased levels of fluctuating asymmetry are correlated strongly to increased risk of orofacial clefting.

© 2021. Published by The Company of Biologists under the terms of the Creative Commons Attribution License (https://creativecommons.org/licenses/by/4.0/).

In Fig. 1, consider whether the addition of sagittal OPT views would optimally illustrate convergent extension.
We did not add in sagittal views except for the one image in Figure 1G. The wireframes (Fig. 1H-M) are the best way to see the extension in the dorsoventral, craniocaudal axes and convergence in the mediolateral axis. At the suggestions of reviewer #1 planes of section for the wireframes have been added as insets. Individual sections can't be used to show this in the craniocaudal axis because section plane can vary. On the other hand, mediolateral distance between the midpoint of the nasal slits can be measured in the frontal plane more consistently. This is mainly why we used this as our main readout for morphogenesis in Figures 3F,G, S1 and S5, S6C.

I didn't understand why mediolaterally separating daughter cells would not be visualised equally from the frontal and the (top down, or axial?) coronal plane. I do expect they wouldn't be seen from the sagittal plane.
This decrease in scoreable cells in the coronal plane may mean that cells are dividing in the cranio-caudal axis which we did capture in the frontal plane. This has been added to the results. Nevertheless even if there were a proportion of cells dividing in the cranio-caudal axis on average the angle was closer to 90 degrees.
The arrowheads in Fig. 2E, D are not pointing at PH3 +ve cells and are not very necessary.
They are there to show cells that are NOT pH positive. This has been changed in legend

The polar plots in Fig. 2G, H are fine, but I wonder if they would be more immediately clear if they were shown as a full circle with symmetrical wedges about the centre so that the reader does not mistakenly wonder whether daughter cells preferentially emerge toward one side.
There is no difference that we can see for most of the bins except for 120-140 degrees. Overall the right and left sides have similar normality plots. We added figure S4A,B with the left and right sided data displayed separately rather than combined.

Since the orientation of cell division does not correspond to convergent extension, could daughter cell intercalation be restore the widening caused by their separation?

Yes this is possible, there could be a balance between intercalation and oriented cell division. However we did not visualize intercalation at 10X as this was too low magnification and cells were too far apart to detect intercalation. We did see some convergence and intercalation at 30X near the nasal slit (Fig. 7A,B,E). Intercalation was also observed in other 63X data. We chose not to include the 63X dataset in the MS since so few cells were analyzed that it was hard to detect patterns of movement.

In the Dynamic cell movements section of the results, it would be helpful to add a sentence describing the imaging method. Please briefly define radius and the purpose of averaging cosine where those concepts are introduced in the results. Please provide a legend for the solid and dashed lines in Fig. 4 H', I'.
The imaging method has been briefly described in the results. The legend for Figure 4 has been updated. Vertical lines represent times of high directional order, disorder and order. The first time when peak order is seen is a solid line, the peak disorder is a line with short dashes, the second peak of order is a dashed line with long dashes.
It may be useful to add that ROCKi disrupts individual cell behaviours in addition or as an alternative to disruption of cell-cell and cell-matrix communication.
This has been edited
Please define k-mean clustering where it is introduced in the results.
An introductory sentence has been added.
It would be helpful to explain a little more how p and q weighting was derived in the results. Does "50 µm area" refer to radius?
Yes you are correct, this has been edited.
In addition to signalling, preprogramming and genetic control, long range coordination of cell movements and symmetry might also be achieved by physical constraints. Please elaborate on the reason that order corresponds to switches between divergence and convergence Although it makes sense that Rho GTPases serve as hubs for signalling cascades that can be disrupted by ROCKi, it wasn't very clear how such a hub might mediate physiological reversal of cell movements.
There are on and off signals depending on whether GEP or GDF is formed.

© 2021. Published by The Company of Biologists under the terms of the Creative Commons Attribution License (https://creativecommons.org/licenses/by/4.0/).

In citing Gros et al, 2010, it may be more appropriate to note they showed FGF promotes, rather than orients, mesenchymal cell movements. It wasn't clear how instructive source and sink cues might correspond to oscillation between convergence and divergence of cell movements. In the case of Wnt5a as a potential source leading to cell convergence, why would the duration be brief and intermittent?
The discussion of sources and sinks has been removed since this was too speculative.

Second decision letter

MS ID#: DEVELOP/2020/193755

MS TITLE: Symmetry and fluctuation of cell movements in neural crest-derived facial mesenchyme

AUTHORS: Adrian Danescu, Elisabeth G. Rens, Jaspreet Rekhi, Johnathan Woo, Takashi Akazawa, Katherine Fu, Leah Edelstein-Keshet, and Joy Richman
ARTICLE TYPE: Research Article

I am happy to tell you that your manuscript has been accepted for publication in Development, pending our standard ethics checks.

Reviewer 1

Advance summary and potential significance to field

The authors use live imaging to uncover intriguing patterns of coordinated cell movements during morphogenesis of the frontonasal mass. Through tracking cell behaviours and performing statistical analyses, the authors demonstrate oscillating patterns of order and disordered cell convergence and divergence.
Importantly, these patterns are disrupted by inhibition of Rho GTPases linking the cell cytoskeleton to coordination of cell movements and facial symmetry. These data underscore cell biological mechanisms that could underly human dysmorphology.
This article is appropriate for publication in Development as it establishes an important framework for further mechanistic cell biological studies of facial morphogenesis.

Comments for the author

Danescu et al. present a much-improved manuscript with significantly increased clarity. The article presented makes an important step toward understanding the cell biological mechanisms that underlie human morphogenesis and facial difference. Their data establish an important perspective to consider the etiology of human disease.

Reviewer 2

Advance summary and potential significance to field

In this manuscript, Danescu et al study the movement of facial mesenchymal cells. They describe a new technique of explant culture that allow for time-lapse imaging of these cells. First, they describe the morphology of the region of interest using 3D reconstruction of chick embryo head, then show that this experimental system allows for the reproduction of the narrowing of the tissue linked to normal morphogenesis. By manual tracking and using computational methods they show that motions of cells alternate between ordered and disordered phases, therefore, displaying a high degree of coordination of cell movements over large distances. Then, they show that these global

© 2021. Published by The Company of Biologists under the terms of the Creative Commons Attribution License (https://creativecommons.org/licenses/by/4.0/).

movements are symmetric between the left and right sides and that they are defined by a succession of divergence and convergence events.
Finally, they show that these divergence and convergence events are oscillating. They show that coordination, symmetry, and periodicity of cellular movements are affected by RhoGTPase inhibitor drug treatment suggesting that they involve cytoskeletal reorganization. The manuscript is particularly interesting because it describes a new culture / time-lapse: image analysis techniques that are suitable to model and therefore study late relatively morphogenesis and pathologies such as cleft palate. The use of new computational tools allowing for probing the coordination of cell movements over large distances is very powerful and potentially useful for a broad scientific community. Experiments are well executed, deeply analyzed and conclusions sound appropriate. Overall, long-distance coordination of cellular movements is understudied although this process is very likely to have critical roles in morphogenesis.

Comments for the author

Some of my concerns have been addressed in the new version of the manuscript. The proliferation rate, cell death, density of the cultured explants do not seem too different between control and treated conditions (and in a reasonable range compared to what we could expect in vivo). Although I still think that a more detailed description of intercalation behavior in control conditions would have considerably improved the manuscript by showing that it mimics in vivo situation I do believe that the manuscript is now suitable for publication in Development.

Reviewer 3

Advance summary and potential significance to field

The authors have answered all of my questions, and I have none further.

Comments for the author

I am looking forward to seeing it published.

© 2021. Published by The Company of Biologists under the terms of the Creative Commons Attribution License (https://creativecommons.org/licenses/by/4.0/).
